# Assessing abundance–suitability models to prioritize conservation areas for the dwarf caimans in South America

**DOI:** 10.1002/ece3.70235

**Published:** 2024-08-29

**Authors:** Andrés L. Rodriguez‐Cordero, Sergio A. Balaguera‐Reina, Brandon A. Gross, Margaret Munn, Llewellyn D. Densmore

**Affiliations:** ^1^ Department of Biological Sciences Texas Tech University Lubbock Texas USA; ^2^ Fort Lauderdale Research and Education Center University of Florida Fort Lauderdale Florida USA

**Keywords:** generalized additive model, generalized linear model, *Paleosuchus palpebrosus*, *Paleosuchus trigonatus*, spatial ecology, species abundance models, species distribution models

## Abstract

Species–environment relationships have been extensively explored through species distribution models (SDM) and species abundance models (SAM), which have become key components to understand the spatial ecology and population dynamics directed at biodiversity conservation. Nonetheless, within the internal structure of species' ranges, habitat suitability and species abundance do not always show similar patterns, and using information derived from either SDM or SAM could be incomplete and mislead conservation efforts. We gauged support for the abundance–suitability relationship and used the combined information to prioritize the conservation of South American dwarf caimans (*Paleosuchus palpebrosus* and *P. trigonatus*). We used 7 environmental predictor sets (surface water, human impact, topography, precipitation, temperature, dynamic habitat indices, soil temperature), 2 regressions methods (Generalized Linear Models—GLM, Generalized Additive Models—GAM), and 4 parametric distributions (Binomial, Poisson, Negative binomial, Gamma) to develop distribution and abundance models. We used the best predictive models to define four categories (low, medium, high, very high) to plan species conservation. The best distribution and abundance models for both *Paleosuchus* species included a combination of all predictor sets, except for the best abundance model for *P. trigonatus* which incorporated only temperature, precipitation, surface water, human impact, and topography. We found non‐consistent and low explanatory power of environmental suitability to predict abundance which aligns with previous studies relating SDM‐SAM. We extracted the most relevant information from each optimal SDM and SAM and created a consensus model (2,790,583 km^2^) that we categorized as low (39.6%), medium (42.7%), high (14.9%), and very high (2.8%) conservation priorities. We identified 279,338 km^2^ where conservation must be critically prioritized and only 29% of these areas are under protection. We concluded that optimal models from correlative methods can be used to provide a systematic prioritization scheme to promote conservation and as surrogates to generate insights for quantifying ecological patterns.

## Graphical abstract

This study identified conservation priorities for the South American dwarf caimans (*Paleosuchus palpebrosus* and *P. trigonatus*) relying on robust species distribution and abundance models. We support that environmental suitability and spatial abundance are not always congruent and cannot be considered reliable surrogates for one another, and independently incorporate relevant information to better understand the spatial ecology and population dynamics towards the improvement of systematic conservation planning of biodiversity.

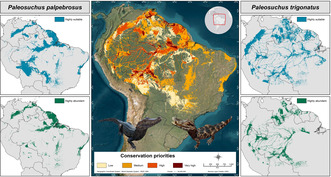

## 1 INTRODUCTION

The functioning of ecosystems depends on both species' presence and structure of local population dynamics within the species distribution range (Waldock et al., 2022). Thus, the incorporation of local abundance patterns provides fundamental information to understand and predict ecological processes aimed at biodiversity conservation (Cavalcante et al., 2022; Zurell et al., 2022). In spatial ecology, occurrence‐based methods (Species Distribution models (SDM)) have been extensively explored to predict environmental suitability and occupied distribution areas (Guisan et al., 2017; Guisan & Zimmermann, 2000; Peterson et al., 2011). In contrast, abundance‐based methods (Species Abundance Models [SAM]) are scarcer as abundance data, are more difficult to obtain due to economic and temporal cost‐effective challenges, and thus, remained limited or elusive for most taxa (Araújo & Williams, 2000; Carrascal et al., 2015; Sagarin et al., 2006; VanDerWal et al., 2009).

Although SAM are less common than SDM, the “abundance–suitability” relationship has been reviewed previously using the “niche” concept as the sets of environmental states inside a multidimensional hypervolume space, within which a species can survive (Hutchinson, 1957). Under this framework, environmental conditions are assumed to affect the species' habitat suitability and the probability of occurrence, with more favorable environmental conditions driving population dynamics to higher species abundance (Araújo & Williams, 2000; Osorio‐Olvera et al., 2020). However, empirical evidence showed that abundance data are not entirely constrained by the properties of niche theory and rather other ecological factors (e.g., transitory states of population, demographic stochasticity, suitability spatial heterogeneity, and Allee effects) can affect local population dynamics and interfere with the expected abundance–suitability relationship (Osorio‐Olvera et al., 2019; Waldock et al., 2022). Consequently, assessments of the abundance–suitability relationship have failed to detect consistent correlation, suggesting that these patterns are not constrained by the same underlying ecological processes (Dallas & Hastings, 2018; Johnston et al., 2015; Mi et al., 2017). In this context, efforts to identify areas to prioritize species conservation incorporating relevant information from both SDM and SAM must be done so that models are more reflective of the interaction between these two population attributes (Mi et al., 2017; Waldock et al., 2022).

Robust regression‐based methods such as Generalized Linear Models (GLM) and Generalized Additive Models (GAM) have been developed and improved to make ecological inferences based on species' environmental requirements and address some of the major biodiversity shortfalls (Guisan et al., 2002; Pollock et al., 2020; Qiao et al., 2015). GLM are model‐driven as the response variable is expected to follow a parametric distribution along the predictors (e.g., Binomial, Poisson, Negative binomial, Gamma), whereas GAM are data‐driven analysis that apply smaller non‐parametric smoothing functions (basis functions) to each predictor and additively estimates the component response (Guisan et al., 2017). GAM constitute semi‐parametric extensions of GLM, and both use link functions to establish a relationship between predictors and response variables (Wood, 2017; Zuur et al., 2009). Both GLM and GAM have been extensively utilized to predict habitat suitability and, to a lesser extent, abundance patterns, providing information on modeling capabilities (e.g., Guisan et al., 2002; Kosicki, 2020; Oppel et al., 2012; Potts & Elith, 2006) and quantifying general relationships between abundance and suitability patterns (Thuiller et al., 2014; VanDerWal et al., 2009; Weber et al., 2017).

Since protocols to estimate relative abundances have been well standardized for many crocodylian species (Balaguera‐Reina et al., 2017; Grigg & Kirshner, 2015; Rodriguez‐Cordero et al., 2019; Seijas & Chávez, 2000; Zucoloto et al., 2021), these taxa represent a promising group for implementing correlative modeling to assess the spatial ecology of each species. Among the smallest South American crocodylians, both Cuvier's dwarf caiman (*Paleosuchus palpebrosus*) and the Smooth fronted caiman (*Paleosuchus trigonatus*) have historically been given marginal attention since they have no commercial value (Ergueta & Pacheco, 1990; Pacheco & King, 1995). Consequently, even though these species are listed as “Least Concern” by the IUCN Red List of Threatened Species and local populations are reported as apparently healthy and abundant throughout their range, much of their spatial ecology, complex habitat use patterns, and quantitative population trends remain relatively unknown (Campos et al., 2019; Magnusson et al., 2019; Marioni et al., 2022).

To address these limitations and to improve our understanding of the spatial ecology of both dwarf caiman species, the main objectives of this research were to: (1) estimate optimal distribution and abundance models to categorize and prioritize conservation areas for the *Paleosuchus* species across their distribution range, (2) assess whether there is a consistent (either positive or negative) and significant relationship between habitat suitability and species abundance, and (3) define a method to reconcile distribution and abundance models so this information can be integrated to prioritize conservation areas for both *Paleosuchus* species. We hypothesized that optimized structures and specifications of GLM and GAM would yield a consistent spatial abundance–suitability correlation with a high explanatory capability of suitability to predict species abundance. Furthermore, we expected an agreement between the areas predicted as both highly suitable and abundant that may warrant protection and that can be used as surrogates to promote effective long‐term species conservation planning of the species.

## 2 METHODOLOGY

### 2.1 Distribution model data acquisition

We compiled an initial occurrences dataset for distribution modeling from published literature, non‐published databases, the most recent IUCN Red List assessments for the species (Campos et al., 2019; Magnusson et al., 2019), and the Global Biodiversity Information Facility (GBIF) (www.gbif.org; Appendices S1 and S2). GBIF records were obtained via *rgbif* package (Chamberlain et al., 2023) in R version 4.2.1 (R Core Team, 2022), selected based on record type (human observations, living specimen, preserved specimen, and material sample) and metadata completeness (country, administrative area, reference, year, event date, responsible of specimen record and identification, locality, latitude, longitude, accepted scientific name, uncertainty, counts, and dataset key). We improved the spatial and temporal reliability of our occurrence dataset by excluding duplicated occurrences, records older than 1979, records with an uncertainty higher than 10 km, erroneous coordinates, coordinates out of the natural range, and records with incongruences between specific locality and location coordinates (Anderson et al., 2016; Balaguera‐Reina et al., 2017; Panter et al., 2020; Zizka et al., 2020). Finally, we reduced spatial clustering by overlapping filtered records with a 1 km^2^ raster layer, selected pixels with at least one record, and estimated the centroid of each selected pixel (Anderson & Gonzalez, 2011; Beck et al., 2014; Boria et al., 2014; Veloz, 2009).

**FIGURE 1:**
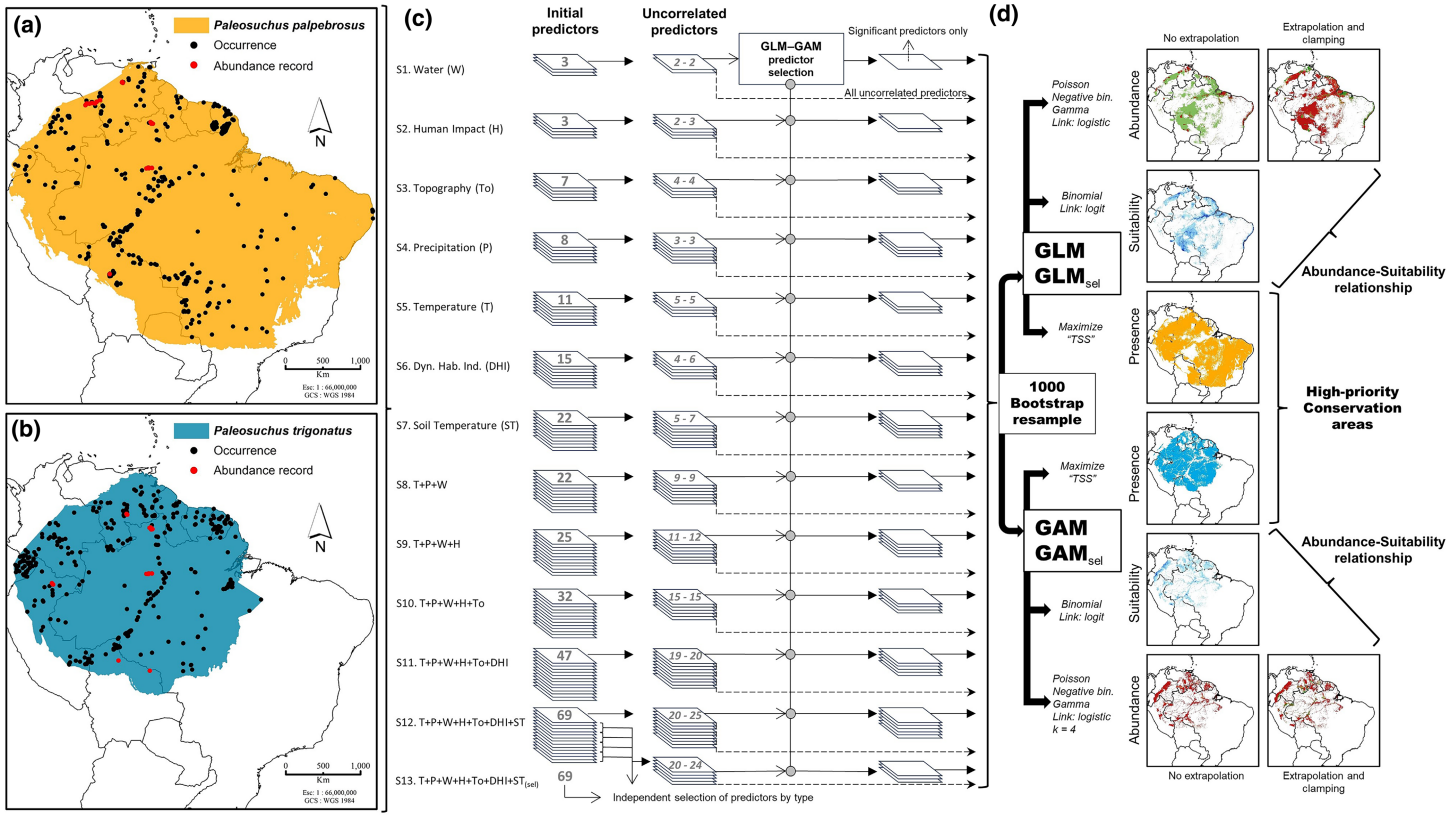
General framework implemented in this study to assess suitability‐abundance relationships and define high‐priority conservation areas for Cuvier's dwarf caiman (*Paleosuchus palpebrosus*) and Smooth fronted caiman (*P. trigonatus*). (a) and (B) abundance records, occurrences, and calibration area. (c) Definition of modeling “structure” by selecting different complexity sets of ecological predictors regarding their type and number of variants used during model fitting. The first number of uncorrelated predictors refers to *P. palpebrosus*, and the second number to *P. trigonatus*, both calculated within the calibration area, using a Pearson correlation coefficient greater than |.7| to define highly correlated predictors. (d) Definition of modeling “specification” using Generalized Linear Models (GLM) and Generalized Additive Models (GAM) to define distribution (suitability and presence/absence maps) and abundance patterns, establish abundance–suitability relationships, and estimate high‐priority conservation areas for both caiman species throughout their potential distribution range.

We defined the calibration area (geographical extension used for background pseudo‐absences sampling; Phillips et al., 2017) of each species by (1) calculating a convex hull based on peripheral occurrences that were within a distance of 800 km for *P. palpebrosus* and 700 km and for *P. trigonatus*, respectively, concentrating the background sampling area around occurrence locations (“background thickening”) and avoid substantial overestimate of the species distribution range (Burgman & Fox, 2003; Vollering et al., 2019), (2) buffering *P. palpebrosus* and *P. trigonatus* hulls 40 and 20 km, respectively, assumed as the maximum annual range movement of each species estimated from daily average movement reports (Marioni et al., 2022); and (3) combining previously buffered hulls with the current distribution extent defined reported by the IUCN Red List for each species (Campos et al., 2019; Magnusson et al., 2019; Figure 1a,b). Finally, we selected a modeling background based on 10,000 randomly defined pseudo‐absences within the species calibration area without including locations of species occurrences and without selecting the same pseudo‐absence location more than once. This pseudo‐absence selection method has been shown to produce the most accurate models when implemented within GLM and GAM and evaluated via the True Skill Statistics (TSS) (Barbet‐Massin et al., 2012).

### 2.2 Abundance model data acquisition

We conducted an initial peer‐review literature search via Google Scholar using the following search terms: “*Paleosuchus palpebrosus*”, “*Paleosuchus trigonatus*”, “distribution model”, and “relative abundance” (in English, Spanish, and Portuguese). Within the reference section of the previously selected publications, we further searched for unpublished reports and theses and attempted to find them online and through requisition from author/s or University repositories (Tables S1 and S2).

Within the available literature, we then searched for standardized spotlight surveys that were used to estimate the number of caimans observed per kilometer of surveyed transect (ind/km) (Bayliss, 1987; Chabreck, 1966; Magnusson, 1982; Messel et al., 1981; Wood et al., 1985). We compiled any available spatial information associated with abundance reports, such as maps or initial and final survey coordinates. Unrectified transects in maps were digitized and orthorectified based on initial and final survey coordinates, georeferenced imagery, and topographic base‐map layers available in ArcMap v10.8.2 (ESRI, 2021). We did this to reconstruct the potential sampling routes based on historical imagery available in Google Earth Pro v7.3.6.9345 (Google Earth Pro, 2022). We overlapped each digitized transect with a 1 km^2^ raster layer and assigned the corresponding abundance value to each intersecting pixel, as well as its longitudinal and latitudinal centroid coordinates. We excluded both animals classified as “Class I” (referring to hatchlings and neonates) as they vary highly across seasons and years due to the low survival rates (Da Silveira et al., 2008; Waddle et al., 2015), and “eyes only” to avoid species misidentification with other sympatric species within our study area (Marioni et al., 2013) (Appendix S2). Unlike distribution models that estimated the probability of species' occurrence conditioned to environmental predictors (Phillips et al., 2009), abundance models were calibrated without background points since they quantified the relationship between species abundances and environmental conditions (Potts & Elith, 2006).

### 2.3 Ecological predictors

We considered ecologically relevant and freely available continuous predictors that describe the primary physical environmental regimes (climate, terrain, soil factors, and nutrient availability) (Franklin, 2009) and terrestrial human footprint (Mu et al., 2022). The 69 selected predictors comprised variants of: (1) global surface water (Pekel et al., 2016), (2) human impact on the environment (Center for International Earth Science Information Network—CIESIN—Columbia University, 2018; Mu et al., 2022; Wildlife Conservation Society—WCS,, & Center for International Earth Science Information Network—CIESIN—Columbia University, 2005), (3) topography (Amatulli et al., 2018; Lehner et al., 2008), (4) precipitation, (5) temperature (Karger et al., 2017), (6) dynamic habitat indices (Hobi et al., 2017), and (7) soil temperature (Lembrechts et al., 2021; van den Hoogen et al., 2022) (Table S3). Predictors were used at ~1 km^2^ resolution and clipped based on the calibration area for each *Paleosuchus* species.

We estimated environmental heterogeneity within the species calibration area based on all 69 ecological predictors. We selected uncorrelated variants (Pearson correlation coefficient *r* < |.7|) within the calibration area of each *Paleosuchus* species via the “*vifcor*” function (using 1000,000 random raster cell values) within the “*usdm*” R package (Naimi et al., 2014) (Table S4). We spatially rarefied distribution data previously selected as pixel centroids at 1, 3, and 5 km based on high, medium, and low environmental heterogeneity to reduce geographic clustering (Rodriguez‐Cordero et al., 2022) via SDMtoolbox v2.0 (http://www.sdmtoolbox.org; Brown et al., 2017). We did not rarify abundance data due to the low number of records.

### 2.4 Modeling framework

Model complexity was defined by modifying two parameters: (1) structure (selection of ecological predictors) and (2) specification (selection of alternative modeling parameterization) (Potts & Elith, 2006). For the former, we constructed models using individual and a gradual combination of ecological predictors (Regos et al., 2019), resulting in 12 sets, each with a different number of predictors, and thus, increased the model calibration complexity as the number of predictors increased. We ran Pearson pairwise comparisons in each complexity set to avoid collinearity among predictors and identified predictor pairs with a linear correlation greater than |.7| (Dormann et al., 2013). We excluded the predictor with the highest Variation Inflation Factor (VIF) (Chatterjee & Hadi, 2006) from each highly correlated pair via the “*vifcor*” function within the “*usdm*” R package (Naimi et al., 2014). We built an extra ecological predictor set (S13) that initially included all 69 predictors and made two correlation analyses to avoid collinearity by first excluding predictors independently by type, and second, by excluding the highly correlated predictors from the previously selected set (Figure 1c). Considering the latter parameter, we used two regression methods (GLM and GAM) and four parametric distributions: binomial (for distribution models), Poisson, Negative binomial, and Gamma (for abundance models) to comparatively assess the effect of different model specifications (Potts & Elith, 2006). We incorporated the two‐step approach by first modeling distributions with a binary response (e.g., presence/absence), and then, modeling abundances conditional to the binary distribution models (Barry & Welsh, 2002), excluding predictions outside the regions classified as “presence” (Figure 1d).

### 2.5 Estimating distribution and abundance through GLM and GAM

Initially, GLM and GAM were constructed using all uncorrelated predictors with each of the 13 predictor sets. Then, GLM complexity was reduced by sequentially excluding predictors until reaching the minimum Akaike Information Criteria (AIC) (Zuur et al., 2009), using the “step” function from the “stats” package (version 4.2.2). For GAM, the selection of the smooth component and predictor selection was achieved using the “double penalty shrinkage approach” (Marra & Wood, 2011), by selecting the argument “*select = TRUE*” in the “*gam*” function from the “*mgcv*” package (version 1.8.2) (Wood, 2017).

We produced species distribution models (suitability and presence/absence) based on the outputs from the GLM using a binomial error distribution and a “logit” link, via “*glm*” function from the “*stats*” package. For GAM we used a binomial parametric distribution using the restricted maximum likelihood (REML), low‐rank thin‐plate splines (bs = “tp”) via the “*gam*” function from the “*mgcv*” package (Marra & Wood, 2011; Wood, 2017), and a basis complexity (*k*) = 4 as it approximates to a third‐degree polynomial as suggested by Guisan et al. (2017) and Hastie et al. (2017). For all our distribution models, we applied an internal iteratively 5‐fold cross‐validation analysis, randomly selecting 80% of occurrences for calibration and the remaining 20% for model evaluation, via the “*crossvalSDM*” function from the “*mecofun*” package (Zurell, 2020). We evaluated model performance using minimum reliable predictability values for the receiver operator characteristics (ROC) of the area under the curve (AUC = 0.7), and true skill statistics (TSS = 0.4) (Araújo et al., 2005; Ruete & Leynaud, 2015). Lastly, we estimated binary responses (presence/absence) using the threshold that maximized the model TSS (sensitivity + specificity −1) (Allouche et al., 2006; Fielding & Bell, 1997).

For species abundance models, we estimated regression coefficients for both GLM and GAM by bootstrapping the data 1000 times using the “boot” function from the “*boot*” package approach (Canty & Ripley, 2021; Efron & Tibshirani, 1998). The bootstrapping approach allowed us to resample the modeling data and provide the least biased estimates of regression coefficients (Potts & Elith, 2006). We did this because of the low number of abundance records, which limited the obtention of an independent evaluation dataset for species abundance models. For GLM, each resample was fitted with a “logistic” link with Poisson and Gamma parametric distributions, via the “*glm*” functions from “*stats*” package (Davison & Hinkley, 1997), and Negative Binomial parametric distribution via the “*glm.nb*” function from “*MASS*” package (Venables & Ripley, 2002). For GAM, each resample was fitted using Poisson, Negative Binomial, and Gamma distributions with a “logistic” link, via the “*gam*” functions from “*mgcv*” package (Wood, 2017). We used transect length as an “*offset*” parameter when fitting both GLM and GAM to account for differences in length. The average of the 1000 regression coefficients for each predictor was used to estimate GLM and GAM abundance values. We used Pearson's correlation coefficient (*r* > .7) as an indicator of agreement between observed (y‐axis) and predicted (x‐axis) values and fitted a simple linear regression to provide information on predictions' bias and consistency (Piñeiro et al., 2008; Potts & Elith, 2006).

We projected abundance estimations into the “presence” area defined by distribution models, conditioning the suitability response to the projected area where the species was predicted to be present (Barry & Welsh, 2002). Due to the skewed distribution of projected abundances as a result of the “logistic” link and extreme predictor values, we bootstrapped projected values 100 times to estimate the “inner fences”, defined as the minimum and maximum percentiles (25th percentile [Q1]‐(1.5*inter‐quartile range) and 75th percentile [Q3] + (1.5*inter‐quartile range), respectively) based on Adjusted Boxplot, which is a robust method to detect outliers within skewed datasets (Hubert & Vandervieren, 2008; Seo, 2006). Outlier detection was done via “*do*” (“*mosaic*” R package) and “*adjboxStat*” (“*robustbase*” R package) functions (Maechler et al., 2023; Pruim et al., 2017). To explore the implications of different model projections, we constrained our predicted abundances to remain within the range of the minimum and maximum percentiles via two methods: (1) “no extrapolation” by discarding values outside the range of the minimum and maximum percentiles and (2) “extrapolation and clamping” by replacing values that were greater or less than the maximum and minimum percentile with the respective limit values (Cobos et al., 2019; Elith et al., 2011).

Additionally, as suggested by Liu et al. (2019), the relative importance of ecological predictors driving species abundances was analyzed via the loss of predictive power excluding each ecological predictor at a time and calculating the mean explained deviance reduction. For distributional models, we used permutation‐based variable‐importance evaluation by applying the Pearson correlation between the original predictions and predictions where one variable has been 100 times randomly permutated (i.e., if the correlation is high, the variable is not important for the model), via the “*bm*_*VariablesImportance*” function of the “*biomod2*” (version 4.2–5‐2) R package (Thuiller et al., 2024).

### 2.6 Estimating abundance–suitability relationship

The abundance–suitability relationship was performed in two ways: (1) we related models that were constructed using the same ecological predictors and (2) we selected the best distribution and abundance models independently of the ecological predictors used as inputs. For the former step, we selected optimal distributional models (AUC > 0.7 and TSS > 0.4) restricting suitability estimations to the regions classified as “presence” by the binary predictions. For abundance, we selected models with high consistency between observed and predicted values (Pearson's coefficient *r* > .7), clipped predictions to the region previously classified as “presence”, and rescaled abundances to continuous values from 0 to 1 (representing low to high abundances, respectively). Finally, suitable and abundance outcome maps were used to test for spatial correlation (Pearson's correlation coefficient analysis) using 2,000,000 raster values for *P. palpebrosus* and 1,000,000 raster values for *P. trigonatus*, which correspond to ~10% of the total number of raster pixels in the “presence” extent. For the latter step, we used the region classified as “presence” by the distributional model to clip both suitability and abundance outcome maps. We rescaled the abundance predictions (0–1) and tested for spatial autocorrelation which provided the significance and abundance variation explained by suitability predictions.

### 2.7 Conservation areas prioritization

We binarized the best continuous distribution and abundance models for each of the *Paleosuchus* species using the threshold that maximized the model TSS (sensitivity + specificity −1) classifying areas as “1” or “highly suitable” as well as classified areas above the median threshold (2nd quartile) as “1” or “highly abundant”, respectively. We then overlapped and summed both suitability and abundance binarized maps (Cavalcante et al., 2022) to create a consensus SDM‐SAM model that we categorized as low (1 = single species present), medium (2 = one species present with high predicted abundances, or both species present), high (3 = both species present with one of them predicted to be abundant), and very high (4 = both species present with high predicted abundances) conservation priorities. Subsequently, we identified critical areas where the conservation of *Paleosuchus* species should be highly prioritized by (1) calculating a minimum convex hull that connected all fragmented areas classified as “very high”, (2) selecting areas classified as “high” within the extent of the hull, and (3) combining both previously selected classified “high” and “very high” priority areas. We used this rationale as we focused on preserving areas where species can be considered in good conditions (both present and abundant), and maintain connectivity to serve as refugia, as well as sources for sustaining viable subpopulations in other locations. Finally, we quantified how much of the critical areas are included within areas with any protection category defined by the United Nations Environment Programme and the International Union for Conservation of Nature categories system (www.protectedplanet.net; UNEP‐WCMC & IUCN, 2024).

## 3 RESULTS

### 3.1 Distribution and abundance through GLM and GAM

**FIGURE 2:**
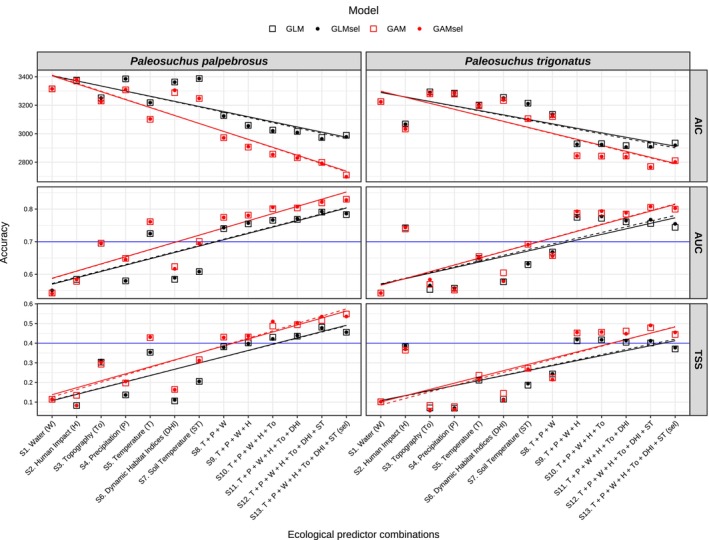
Evaluation of distribution model performances measured by the Akaike Information Criteria (AIC), receiver operator characteristics of the Area Under the Curve (AUC), and the True Skill Statistics (TSS). Sets of predictors (x‐axis) are ordered by increasing complexity regarding the number of predictor variants from left to right, accounting for individual predictor types (models S1–S7) and a gradual combination of different predictor types (models S8–S13). AIC shows how informative the model is based on the number of parameters and complexity. The lower the AIC, the better the model fits the data. AUC and TSS correspond to independent‐threshold and dependent‐threshold metrics, respectively. The blue line corresponds to the minimum levels of reliability for model predictability performance based on the AUC (0.7) and TSS (0.4). Black and red lines correspond to linear fits for Generalized Linear Models (GLM) and Generalized Additive Models (GAM), respectively. Solid lines correspond to models that used all uncorrelated predictors in each predictor set (denoted as “GLM” and “GAM”), whereas dashed lines correspond to models that used uncorrelated predictors, from which most significant were selected to be fitted in each model (denoted as “GLM_sel_” and “GAM_sel_”).

For distribution models, we initially collected 1697 occurrence records for *Paleosuchus palpebrosus* and 2055 for *P. trigonatus*, from which 405 and 386 were selected after data cleaning, respectively (Figure 1a,b). With these data, we estimated 52 distributional binomial models for each *Paleosuchus* species based on identical modeling structures and specifications. A total of 23 (44.2%) models showed minimally optimal predictive performance (both AUC > 0.7 and TSS > 0.4) for *P. palpebrosus*, with the best distributional model achieved via GAM including all predictor sets (GAM‐S13, AUC = 0.830, TSS = 0.548). For *P. trigonatus*, 18 (34.6%) models showed reliable AUC and TSS values with the highest predictive performance achieved via GAM and including all predictor sets (GAM_sel_‐S12, AUC = 0.808, TSS = 0.490). We also found that optimal models for both *Paleosuchus* species were yielded when using combinations of ecological predictor sets. The single exception was for *P. palpebrosus*, where model S5 (“Temperature” [T] variants only) achieved optimal performance via GAM and GAM_sel_. Regarding model specification, our results showed that optimal distributional GAM resulted in higher predictive performances than GLM, except for models S2 and S8 for *P. trigonatus*, where both GLM and GLM_sel_ had higher predictive accuracy than their counterpart GAM and GAM_sel_ (Figure 2; Tables S5 and S6).

**FIGURE 3:**
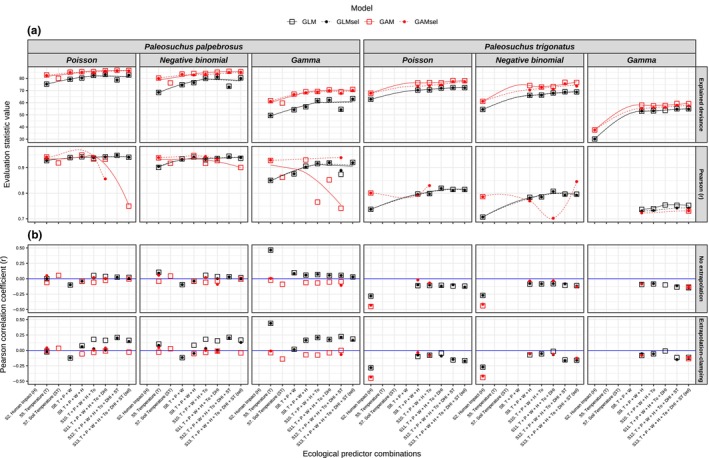
**(**a) Top facets correspond to the amount of variation (percentage) explained by different ecological predictor sets with different specifications during model calibration. The bottom facets indicate the consistency between observed and predicted abundances via Pearson's correlation coefficient (*r*). Models with *r* < .7 were excluded from the analysis. Black and red lines correspond to loess smoother fits for Generalized Linear Models (GLM) and Generalized Additive Models (GAM), respectively. Solid lines correspond to models that used all uncorrelated predictors in each predictor set (“GLM” and “GAM”), whereas dashed lines correspond to models that used uncorrelated and significant selected predictors that were fitted into each model (“GLM_sel_” and “GAM_sel_”). (b) Correlation coefficients between optimal abundance and suitability models that had significant correlations (*p* < .05). Top facets correspond to abundance projections via the “no extrapolation method”, whereas bottom facets correspond to projections via the “extrapolation and clamping” method. The blue line represents the non‐correlation value (0), above and below which correlations between suitability and abundance were significantly positive and negative, respectively. Sets of predictors (x‐axis) are ordered by increasing complexity regarding the number of predictor variants from left to right, accounting for individual predictor types (models S1–S7) and a gradual combination of different predictor types (models S8–S13). The set of predictors in models S1, S3, S4, and S6 were not included in the analysis since no distributional models achieved minimally optimal evaluation parameters (AUC > 0.7 and TSS > 0.4).

For abundance models, we gathered 44 and 43 counting transects for *P. palpebrosus* and *P. trigonatus*, respectively. After overlapping each digitized transect with a 1 km^2^ raster layer, we obtained 670 abundance records within 272 intersecting pixels for *P. palpebrosus* and 942 abundance records within 276 pixels for *P. trigonatus*. We estimated 87 abundance models (42 GLM and 45 GAM) for *P. palpebrosus* and 71 models (35 GLM and 36 GAM) for *P. trigonatus*, based on the sets of predictors combinations that we used to construct minimally optimal distribution models (AUC > 0.7 and TSS > 0.4). Our results showed higher levels of abundance variations being explained via GAM than GLM, with predictive performance increasing as the number of predictors increased in the model structure. For *P. palpebrosus*, the average explained deviance was 71.3% for GLM and 78% for GAM, whereas for *P. trigonatus*, the average explained deviance was 62.1% for GLM and 66.8% for GAM. Despite the higher levels of deviance explained by GAM than GLM, the former algorithm showed higher discrepancies between predicted and observed abundances (Pearson's correlation *r* < .7). Thus, 22 (48.9%) and 25 (69.4%) GAM models were excluded for *P. palpebrosus* and *P. trigonatus*, respectively, whereas only 2 GLM (5.7%) were excluded for *P. trigonatus*. Furthermore, GLM showed a higher average correlation between predicted and observed abundances (*r* = .92 for *P. palpebrosus* and *r* = .78 for *P. trigonatus*) than GAM (*r* = .89 for *P. palpebrosus* and *r* = .77 for *P. trigonatus*). The highest consistency between observed and predicted values was obtained via Poisson GLM using all predictors sets (GLM_sel_‐S12, *r* = .95) for *P. palpebrosus*, and via Poisson GAM using temperature, precipitation, water, human impact on the environment, and topography variants (GAM_sel_‐S10, *r* = .85) for *P. trigonatus* (Figure 3a; Tables S7 and S8).

### 3.2 Abundance–suitability relationship

The assessment of the relationship between distribution and abundance models that used the same ecological predictor inputs was restricted to 110 optimal models. Our results showed low Pearson correlation coefficients (|*r*| < .7) and yielded mixed evidence between positive and negative abundance–suitability relationships. Regarding the analysis using abundance predictions via the “no extrapolation” method, we found four non‐significant relationships, whereas 37 and 69 models had significantly positive and negative abundance–suitability relationships, respectively. Considering the “extrapolation and clamping” method, we found one non‐significant relationship, 42 had positive and 67 had negative correlations (Figure 3b).

We found similar results when restricting the analysis to the best predictive performance models in terms of non‐consistent and low explanatory power between abundance and suitability. For *P. palpebrosus*, the best distributional model (GAM‐S13; AUC = 0.830, TSS = 0.548) and abundance (Poisson GLM_sel_‐S12; *r* = .949) models were constructed using a combination of all predictor types. For the former model, we found that “elevation” and “precipitation seasonality” predictors were most critical, whereas for the latter, “mean monthly precipitation amount of the warmest quarter” and “soil temperature seasonality”, were the most important predictors that affected species occurrence. The correlation between the best distributional and abundance models (GAM‐S13 and Poisson GLM_sel_‐S12, respectively) was significantly positive for both “no extrapolation” (*r* = .009, *p* < .001, CI 95% = 0.007 and 0.011) and “extrapolation and clamping” (*r* = .094, *p* < .001, CI 95% = 0.091 and 0.095) methods (Figure S2).

For *P. trigonatus*, the best distributional model (GAM_sel_‐S12; AUC = 0.809, TSS = 0.493) included a combination of all predictor types, with “isothermality” and “human footprint” being the most important predictors. The best abundance model (Poisson GAM_sel_‐S10; *r* = .853) incorporated a combination of 5 predictor types (temperature, precipitation, global surface water [excluded after variable selection], human impact on the environment, and topography) with “temperature seasonality” and “mean monthly precipitation amount of the coldest quarter” being the most influential predictor variants. The abundance–suitability correlation yielded significantly negative outcomes for “no extrapolation” (*r* = −.013, *p* < .001, CI 95% = −0.015 and −0.011) and “extrapolation and clamping” (*r* = −.020, *p* < .001, CI 95% = −0.022 and −0.018) abundance predictive methods (Figure S2). These results suggested that high habitat suitability and species abundance are not always congruent, and suitability has low explanatory power of abundance patterns. Variable importance and partial response curves of the selected distribution and abundance models are found in Appendix S1 (Figures S3–S7).

### 3.3 Conservation areas prioritization

**FIGURE 4:**
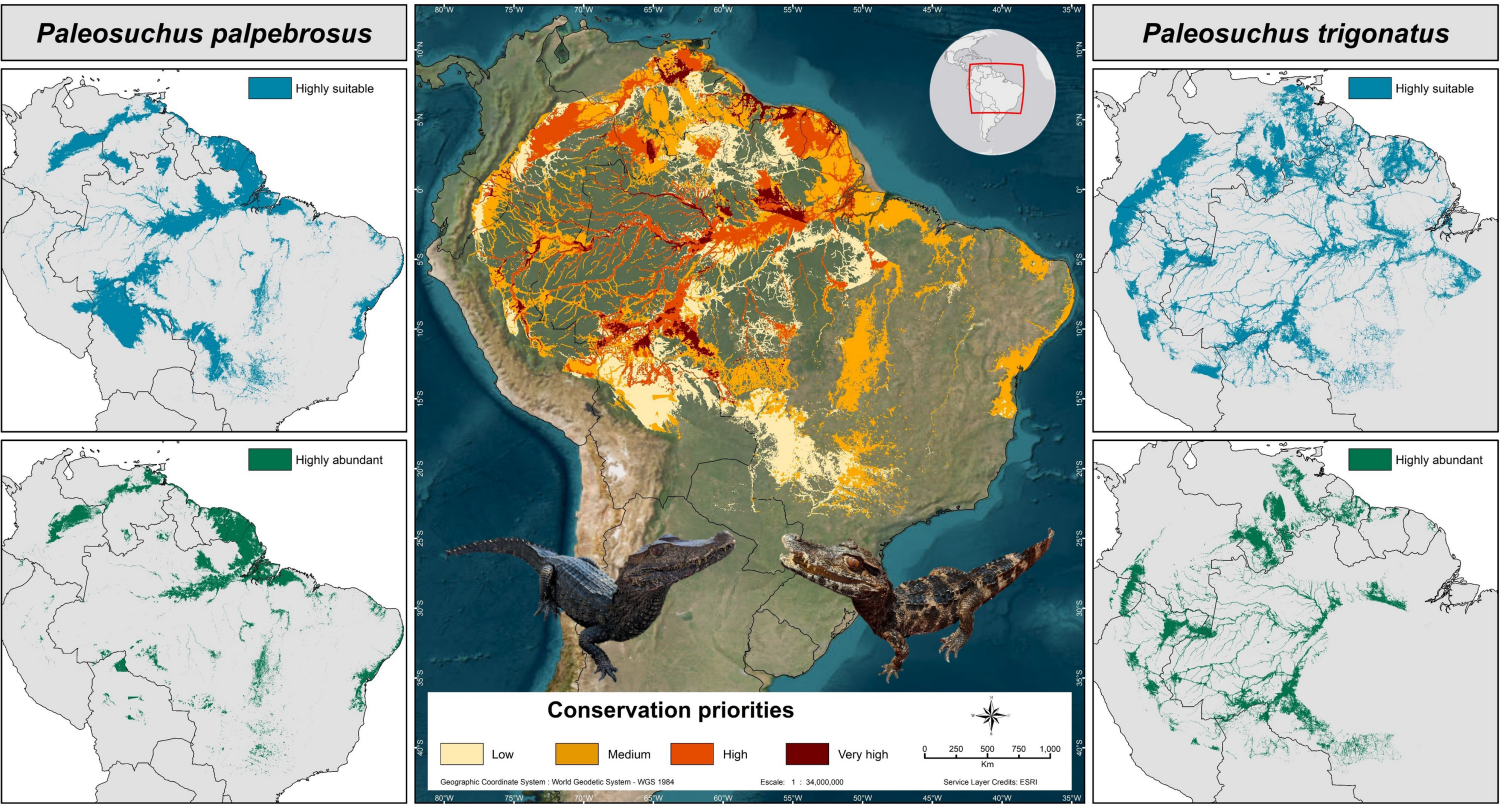
Geographic projection of the prioritization of conservation areas for *Paleosuchus palpebrosus* and *P. trigonatus* estimated via selecting and then combining the best predictive performance distributional and abundance models. Regions predicted as “Highly suitable” (in blue) correspond to areas classified as “presence” based on Binary dependent‐threshold models that maximized the True Skill Statistic (TSS). Regions where the species are predicted to be “highly abundant” (in green) were obtained from continuous species abundance models that were binarized using the median (2nd quartile) as a classifier threshold. Conservation priorities correspond to the number of binarized models that agreed on highly suitable habitats and where the species is predicted to be highly abundant, with values of low (single species present), medium (one species present with high predicted abundances or both species present only), “high” (both species present with one of them predicted to be highly abundant), and “very high” (both species present with high predicted abundances). Left: *P. palpebrosus*; Right: *P. trigonatus*. Photo credit: Andre Rocha.

**FIGURE 5:**
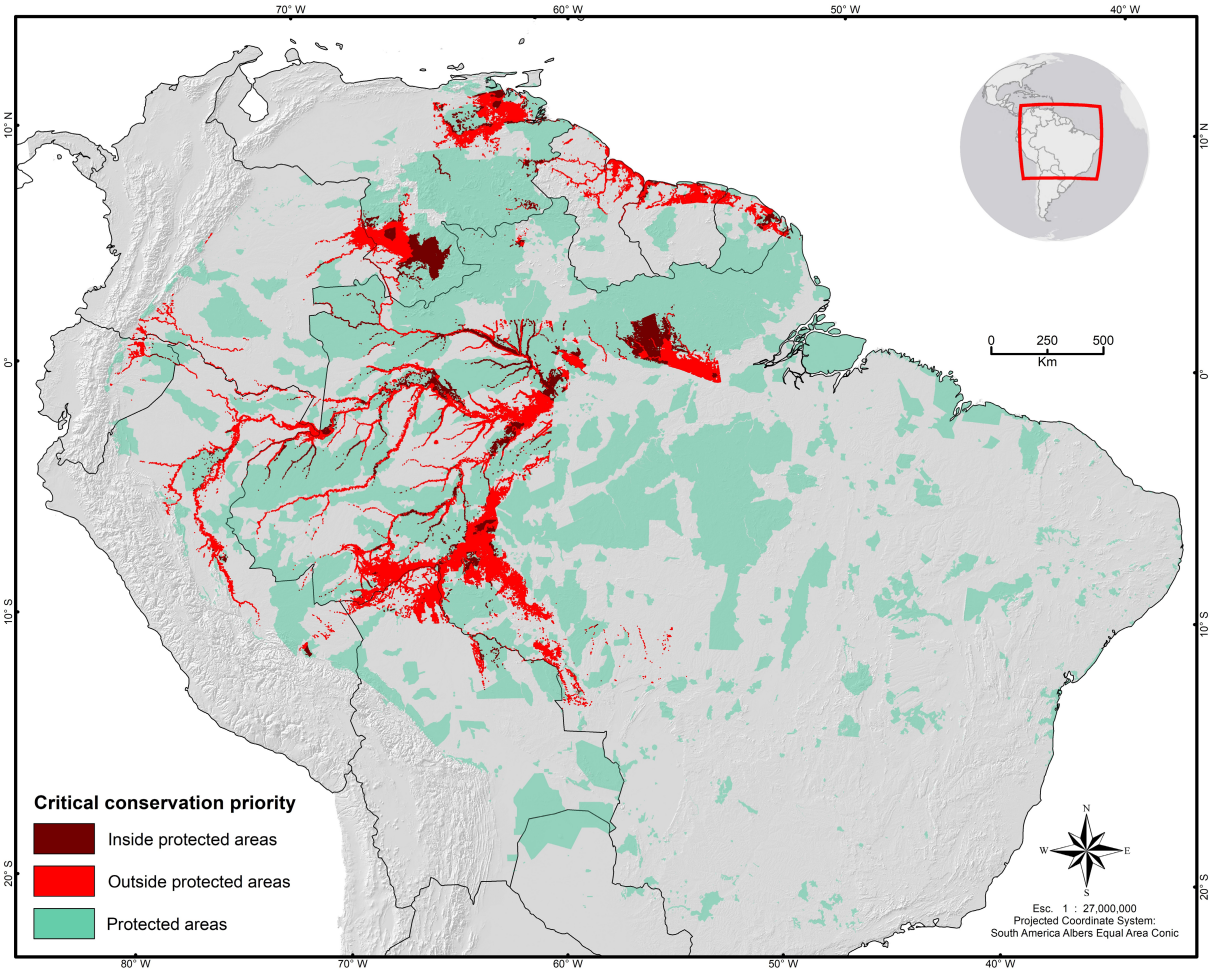
Geographic projection of critical conservation areas that are currently under protection (in brown) and that are found outside protected areas (in red) based on the United Nations Environment Programme and the International Union for Conservation of Nature Protected Areas categories system (in green) (www.protectedplanet.net; UNEP‐WCMC & IUCN, 2024).

Even though correlation analysis showed that distribution and abundance models are not correlated, we extracted and combined the most relevant information from them to create a consensus model that allowed us to estimate areas to be prioritized for species conservation. We estimated a potential distribution area of 1,993,865 km^2^ for *P. palpebrosus* in which at least 50.3% are areas with highly abundant populations of the species (1,003,578 km^2^). For *P. trigonatus*, the potential distribution was estimated at 1,368,089 km^2^ with a core area of 683,174 km^2^ (~49.9%) in which the species is predicted to be highly abundant. We identified 2,790,583 km^2^ to be categorized into different levels of conservation priorities for the targeted taxa, with 1,105,079 km^2^ (39.6%) classified as “low” (single species present), 1,192,809 km^2^ (42.7%) as “medium” (one species present and abundant or both species present), 414,674 km^2^ (14.9%) as “high” (both species present with one being abundant), and 78,021 km^2^ (2.8%) as “very high” (both species present and abundant) (Figure 4; Table 1). We delineated critical conservation areas encompassing 279,338 km^2^ where the conservation of *Paleosuchus* species should be highly prioritized, distributed within Brazil (165,622 km^2^, 59.3%), Venezuela (59,929 km^2^, 20.4%), Bolivia (14,721 km^2^, 5.3%), Peru (12,397 km^2^, 4.4%), Suriname (10,054 km^2^, 3.6%), Colombia (6548 km^2^, 2.3%), Guyana (6101 km^2^, 2.2%), French Guiana (5344 km^2^, 1.9%), and Ecuador (1623 km^2^, 0.6%). We showed that 29% (81,684 km^2^) of critical conservation areas are under protection, whereas 71% (197,653 km^2^) are found outside protected areas (Figure 5).

**TABLE 1:** Difference between the extent of the areas estimated for the conservation priorities of *Paleosuchus palpebrosus* and *Paleosuchus trigonatus* based on ecological suitability and species abundance.

Conservation priority	Area (km^2^)										
	Bolivia	Brazil	Colombia	Ecuador	French Guiana	Guyana	Peru	Suriname	Paraguay	Trinidad & Tobago	Venezuela
Low	269,861	566,140	92,837	27,005	2605	22,036	35,472	21,381	735	18	66,989
Medium	35,382	675,119	86,250	27,490	48,121	36,406	100,147	26,813	106	794	156,181
High	17,323	204,694	69,929	1366	26,481	1151	11,649	28,328	—	—	53,753
Very high	4672	43,804	868	330	1287	4976	3484	6957	—	—	11,643
TOTAL	327,238	1,489,757	249,884	56,191	78,494	64,569	150,752	83,479	843	814	288,566

## 4 DISCUSSION

Our study is the first attempt to identify conservation priorities for two crocodylian species with low knowledge of their spatial ecology, relying on robust distribution models and predicting abundances conditional to species presence. Due to the underdevelopment of abundance‐based models relative to occurrence‐based models, guidance on modeling abundance spatial variation (varying both model structure and regression analysis specifications) remains scarce (Waldock et al., 2022), and was not tested thoroughly for any crocodylian species besides this current study. Thus, the robustness of our approach constitutes a useful tool for managers and conservation practitioners that can guide future conservation planning and provide insights in spatial ecology and biogeography.

Our prioritization approach was restricted to the best distributional and abundance models. For *Paleosuchus palpebrosus*, we selected GAM‐S13 for distribution and Poisson GLMsel‐S12 for abundance. Both models used a combination of all predictor types as inputs (temperature, precipitation, global surface water, human impact on the environment, topography, dynamic habitat indices, and soil temperature). For *P. trigonatus*, GAM_sel_‐S12 optimized distribution predictions and included a combination of all predictor types, whereas Poisson GAMsel‐S10 was selected as the best abundance model, which incorporated a combination of 4 predictor types (temperature, precipitation, human impact on the environment, and topography). From this perspective, we estimated 2,790,583 km^2^ that were categorized as low (39.6%), medium (42.7%), high (14.9%), and very high (2.8%) conservation priorities, and identified 279,338 km^2^ where conservation effort for *Paleosuchus* species should be highly prioritized, distributed in Brazil (59.3%), Venezuela (20.4%), Bolivia, Peru, Suriname, Colombia, Guyana, French Guiana, and Ecuador (20.3%) where the conservation of the targeted taxa should be highly prioritized.

We argue that biodiversity conservation is best met by managing single species instead of entire ecosystems since this method provides explicit analytical and monitoring data to assess the success of conservation efforts (Mace et al., 2007). However, the information used for analyzing conservation efforts must be robust and descriptive of the species' natural conditions or at least the uncertainty of the metric must be understood. For instance, a study on prioritization models and assessment of protected areas' effectiveness in the conservation of worldwide crocodylians suggested that both *Paleosuchus* species are well represented within protected areas (Lourenço‐de‐Moraes et al., 2023), which implies that there is no need for conservation efforts in this regard for these species. However, our study showed that around 71% of the areas where the species can be considered in good condition based on habitat suitability and abundance modeling are outside of any category of protection and do not have any type of regularized protection to ensure their conservation. In this example, the broad differences between Lourenço‐de‐Moraes et al. (2023) and our analysis derived from the quality of the data used as the former study relied conclusions on IUCN Red List distribution polygons which are highly inaccurate to describe the actual species distribution and has no consideration of any other population attribute, whereas the latter used a robust method that accurately defined what could be described as the distribution of both species based on presence and abundance. Thus, since conservation efforts need evidence‐based planning, we emphasize the importance of the development of finer‐scale strategies and incorporating relevant species‐specific data in consensus models to prioritize the allocation of flexible funding to promote species conservation efforts and minimize biodiversity loss (Brooks et al., 2006). Furthermore, crocodylians are considered flagship and umbrella species, and thus, efficient conservation actions might protect a large number of coexisting species and their local environments (Lourenço‐de‐Moraes et al., 2023).

In our study, we also explored the capacity of suitability habitat models to predict abundance patterns within the species distributional range focused on developing a finer‐scale conservation strategies framework. However, we failed to detect a consistent relationship between abundance and suitability since regardless of the use of GLM, GAM, and the individual or gradual combination of ecological predictor types yielded mixed evidence between weak positive and negative relationships. These results were not affected by whether spatial correlation was assessed between single independent abundance and suitable models with the best predictive performance. Consequently, our findings support that environmental suitability and spatial abundance are not always congruent (Waldock et al., 2022), cannot be considered reliable surrogates for one another (Dallas & Hastings, 2018), and independently incorporate relevant information to better understand the spatial ecology and population dynamics towards the improvement of systematic conservation planning.

Similarly to most vertebrate taxa around the world, we were confronted with limited data and had a lower geographic representation of abundances compared with species occurrence (Araújo & Williams, 2000). Although abundance in crocodylian populations has been collected based on standardized methods (Bayliss, 1987; Chabreck, 1966; Magnusson, 1982; Messel et al., 1981; Wood et al., 1985) and have been improved based on new technologies, specific habitats, and ecological information required (e.g., Balaguera‐Reina et al., 2018; Fujisaki et al., 2011; Fukuda et al., 2013; Pacheco, 1996; Sai et al., 2016; Thorbjarnarson et al., 2000), non‐commercially valuable species such as the dwarf caimans have only been given marginal attention (Ergueta & Pacheco, 1990; Pacheco & King, 1995). Consequently, since small sample sizes may yield erroneous density estimations (Yañez‐Arenas et al., 2014) or estimate overconfident models (Waldock et al., 2022), the scarcity of abundance data for crocodylian species that are not subject to management and sustainable use programs constitutes a major constraint to filling knowledge gaps on species abundance distribution patterns.

To overcome this shortcoming, we implemented the method suggested by Welsh et al. (1996) and Barry and Welsh (2002) where distributional patterns were initially modeled, followed by abundance estimation conditional to the presence of the species. Although this strategy might be useful to reduce uncertainty, we emphasize the need to further explore the effect of sample size on abundance‐based models. For example, these caveats could be explored with future studies that include other species from the *Alligatoridae* family whose abundances have been better monitored, such as *Caiman yacare* (Campos et al., 2020), *Caiman crocodilus* (Balaguera‐Reina & Velasco, 2019), or *Alligator mississippiensis* (Elsey et al., 2019). We highlight the relevance of assessing entire assemblages (Hidasi‐Neto et al., 2020), to account for ecological constraints of estimation methods, and incorporate a thorough integrative analysis to obtain comparable data to understand species distribution and abundances patterns (Araújo & Williams, 2000; Balaguera‐Reina et al., 2018). Furthermore, we highlight the need to continue the collection of abundance data or explore potential methods to reduce bias when making density estimations (e.g., Yañez‐Arenas et al., 2014). Future research and conservation efforts could be improved by incorporating major threats not only to crocodylian species but also to other wildlife, such as climate change and habitat degradation. Nonetheless, this approach would only be useful if both current ecological predictors and threats could be accurately predicted into the future (Araújo et al., 2002).

Our findings showed that individual sets of predictors led to low predictive performances, whereas the gradual combination of different sets of predictors and the increase of predictor variants consistently increased the accuracy of distributional models (Figure 2). As expected, our results are consistent with studies that showed significant effects of model structure and the choice of explanatory variables on model performance and transferability (Pratt et al., 2022; Zarzo‐Arias et al., 2022). Interestingly, many studies that implemented distribution models used the same sets of predictors without assessing the effects of different combinations (Regos et al., 2019). To ensure the ecological significance and transferability success of Species Distribution Models (SDM) and Ecological Niche Models (ENM), the selection of predictors needs to avoid the assumption of a fitted equilibrium (Guisan & Theurillat, 2000; Jones et al., 2016), and account for both direct and indirect contingent nature of species‐predictor relationships (Austin, 2007; Gillson et al., 2013).

Overall, we found that both GLM and GAM increased their predictive performance when gradually combining different sets of predictors. Moreover, it would be expected that GAM would yield better predictions than GLM because even though they are both parameterized to fit a linear parametric distribution, GAMs automatically select the polynomial transformation via “smoothers” applied to some predictors (Guisan et al., 2002). Nonetheless, for distribution models, we found that GAM had higher discriminatory power between areas of presence and absence (lowest AIC and highest AUC and TSS) (Figure 2; Tables S5 and S6), whereas GLM performed consistently better for abundance models, yielding higher correlations between observed and predicted values (Figure 3; Tables S7 and S8). Similar patterns were found in other studies, suggesting that GLMs could estimate abundance patterns more accurately than GAM (Waldock et al., 2022; Yu et al., 2013). However, Oppel et al. (2012) found opposite results, indicating better performance of GLM for distributional data and GAM for abundance data. We suggest that this difference might be due to the targeted species (migratory seabird *Puffinus mauretanicus*), ecosystem (Balearic archipelago in the western Mediterranean), and ecological predictors (dynamic oceanographic data) used by Oppel et al. (2012). Consequently, we can conclude that depending on the distributional patterns of the species records (e.g., occurrences and abundance locations), the optimal modeling method (structure and specification) might differ depending on the target species and ecological predictor selection (Yoon & Lee, 2021; Zarzo‐Arias et al., 2022).

We found that the selection process of uncorrelated predictors and identification of significant predictors while model fitting were important considerations to increase the predictive ability for both GLM and GAM. Nevertheless, studies have shown that high accuracy produced by the increase in the number of predictors might result in overfitting models, variable redundancy, and increased uncertainty (Beaumont et al., 2005; Synes & Osborne, 2011). These effects might be problematic when the main objective of the models is the extrapolation of distributions to novel environmental conditions. To overcome these issues, we recommend using other evaluation parameters besides the AUC (e.g., calculate omission and commission errors separately) (Synes & Osborne, 2011) or to evaluate model performance against independent data (e.g., occurrences and environmental conditions not included during model calibration) (Araújo et al., 2005). Although these suggestions may be included in future studies, herein, however, we attempted to make an exploratory analysis comparing the trade‐off between different model structures and specifications using the same current available occurrence and abundance dataset.

This study was able to find evidence to support our hypothesis that the incorporation of additional predictors besides commonly used temperature and precipitation would improve the prediction accuracy of the species realized niche within the limits of the calibration dataset. Nevertheless, complexity might decrease the model's predictive performance within novel environments, and thus affect the interpretability of distribution and abundance estimations. Therefore, the degree of complexity for distribution and abundance models must balance out both calibration accuracy and performance of predictions (interpretability; Venables & Dichmont, 2004). For distribution models, we suggest the incorporation of more relevant predictors when using GLM and GAM if both the amount and representativeness of sampling locations are appropriate. For abundance models, we suggest using GLM for inferences when dealing with scarcity of data and novel environments, since our results showed uniform estimations regardless of model structure and specifications. Future studies including a detailed assessment of regression models' performance and transferability to novel environments is also warranted, incorporating species and entire assemblages with high‐quality abundance estimates.

Finally, this work supports previous studies in that presence‐based distribution and abundance models should systematically guide sustainable management planning and conservation prioritization efforts (Cavalcante et al., 2022; Lourenço‐de‐Moraes et al., 2023; Pollock et al., 2020; Waldock et al., 2022), not only to protect crocodylian species but to prevent widespread loss of concurrent biodiversity and their habitats. Although our findings and datasets are directly related to both *Paleosuchus* species, the same principles should be relevant for other crocodylian species and even other vertebrates, assuming that the amount, quality, and representativeness of predictors are minimally optimal for these assessments. Having considered our research's strengths and limitations, it is important to emphasize that spatial biodiversity models constitute a critical tool to potentially overcome shortfalls that are yet to be explored (e.g., scarcity of data and sampling gaps across the taxon's geographic distribution extent).

## AUTHOR CONTRIBUTIONS

**Andrés L. Rodriguez‐Cordero:** Conceptualization (lead); data curation (lead); formal analysis (lead); investigation (lead); methodology (lead); visualization (lead); writing – original draft (lead); writing – review and editing (lead). **Sergio A. Balaguera‐Reina:** Conceptualization (equal); formal analysis (equal); investigation (equal); methodology (equal); supervision (equal); writing – review and editing (equal). **Brandon A. Gross:** Investigation (equal); validation (equal); writing – review and editing (equal). **Margaret Munn:** Data curation (equal); validation (equal). **Llewellyn D. Densmore III:** Funding acquisition (lead); investigation (equal); supervision (lead); validation (equal); writing – review and editing (equal).

## Supporting information


Appendix S1.

Appendix S2.

Appendix S3.

## Data Availability

The occurrences database, links for the ecological predictors, R script, and relevant information used in this article are available in Appendices S1, S2, and S3.

## References

[ece370235-bib-0001] Allouche, O. , Tsoar, A. , & Kadmon, R. (2006). Assessing the accuracy of species distribution models: prevalence, kappa and the true skill statistics (TSS). Journal of Applied Ecology, 43, 1223–1232. 10.1111/j.1365-2664.2006.01214.x

[ece370235-bib-0002] Amatulli, G. , Domisch, S. , Tuanmu, M.‐N. , Parmentier, B. , Ranipeta, A. , Malczyk, J. , & Jetz, W. (2018). A suite of global, cross‐scale topographic variables for environmental and biodiversity modeling. Scientific Data, 5(1), 180040. 10.1038/sdata.2018.40 29557978 PMC5859920

[ece370235-bib-0003] Anderson, R. P. , Araújo, M. , Guisan, A. , Lobo, J. M. , Martínez‐Meyer, E. , Peterson, T. , & Soberón, J. (2016). Are species occurrence data in global online repositories fit for modeling species distributions? The case of the Global Biodiversity Information Facility (GBIF). Final report of the task group on GBIF data fitness for use in distribution modelling.

[ece370235-bib-0004] Anderson, R. P. , & Gonzalez, I. (2011). Species‐specific tuning increases robustness to sampling bias in models of species distributions: an implementation with Maxent. Ecological Modelling, 222(15), 2796–2811. 10.1016/j.ecolmodel.2011.04.011

[ece370235-bib-0005] Araújo, M. B. , Pearson, R. G. , Thuiller, W. , & Erhard, M. (2005). Validation of species–climate impact models under climate change. Global Change Biology, 11(9), 1504–1513. 10.1111/j.1365-2486.2005.01000.x

[ece370235-bib-0006] Araújo, M. B. , & Williams, P. H. (2000). Selecting areas for species persistence using occurrence data. Biological Conservation, 96(3), 331–345. 10.1016/S0006-3207(00)00074-4

[ece370235-bib-0007] Araújo, M. B. , Williams, P. H. , & Fuller, R. J. (2002). Dynamics of extinction and the selection of nature reserves. Proceedings of the Royal Society of London. Series B: Biological Sciences, 269(1504), 1971–1980. 10.1098/rspb.2002.2121 PMC1691129 12396495

[ece370235-bib-0008] Austin, M. (2007). Species distribution models and ecological theory: a critical assessment and some possible new approaches. Ecological Modelling, 200(1–2), 1–19. 10.1016/j.ecolmodel.2006.07.005

[ece370235-bib-0009] Balaguera‐Reina, S. A. , Espinosa‐Blanco, A. S. , Morales‐Betancourt, M. A. , Seijas, A. E. , Antelo, R. , & Densmore, L. D., III . (2017). Conservation status and regional habitat priorities for the Orinoco crocodile: Past, present, and future. PLoS One, 12(2), e0172439. 10.1371/journal.pone.0172439 28234956 PMC5325271

[ece370235-bib-0010] Balaguera‐Reina, S. A. , & Velasco, A. (2019). Caiman crocodilus. The IUCN Red List of Threatened Species, 2019, e.T46584A3009688. 10.2305/IUCN.UK.2019-1.RLTS.T46584A3009688.en

[ece370235-bib-0011] Balaguera‐Reina, S. A. , Venegas‐Anaya, M. D. , Rivera‐Rivera, B. , Morales Ramírez, D. A. , & Densmore, L. D., III . (2018). How to estimate population size in crocodylians? Population ecology of American crocodiles in Coiba Island as study case. Ecosphere, 9(10), e02474. 10.1002/ecs2.2474

[ece370235-bib-0012] Barbet‐Massin, M. , Jiguet, F. , Albert, C. H. , & Thuiller, W. (2012). Selecting pseudo‐absences for species distribution models: how, where and how many? Methods in Ecology and Evolution, 3(2), 327–338. 10.1111/j.2041-210X.2011.00172.x

[ece370235-bib-0013] Barry, S. C. , & Welsh, A. H. (2002). Generalized additive modelling and zero inflated count data. Ecological Modelling, 157, 179–188. 10.1016/S0304-3800(02)00194-1

[ece370235-bib-0014] Bayliss, P. (1987). Survey methods and monitoring within Crocodile Management Programmes. In G. J. W. Webb , S. C. Manolis , & P. J. Whitehead (Eds.), Wildlife Management: Crocodiles and Alligators (pp. 157–175). Surrey Beatty & Sons. http://refhub.elsevier.com/S2351‐9894(20)30747‐2/sref4

[ece370235-bib-0015] Beaumont, L. J. , Hughes, L. , & Poulsen, M. (2005). Predicting species distributions: use of climatic parameters in BIOCLIM and its impact on predictions of species' current and future distributions. Ecological Modelling, 186(2), 250–269. 10.1016/j.ecolmodel.2005.01.030

[ece370235-bib-0016] Beck, J. , Böller, M. , Erhardt, A. , & Schwanghart, W. (2014). Spatial bias in the GBIF database and its effect on modelling species' geographic distributions. Ecological Informatics, 19, 10–15. 10.1016/j.ecoinf.2013.11.002

[ece370235-bib-0017] Boria, R. A. , Olson, L. E. , Goodman, S. M. , & Anderson, R. P. (2014). Spatial filtering to reduce sampling bias can improve the performance of ecological niche models. Ecological Modelling, 275, 73–77. 10.1016/j.ecolmodel.2013.12.012

[ece370235-bib-0018] Brooks, T. M. , Mittermeier, R. A. , da Fonseca, G. A. B. , Gerlach, J. , Hoffmann, M. , Lamoreux, J. F. , Mittermeier, C. G. , Pilgrim, J. D. , & Rodrigues, A. S. L. (2006). Global Biodiversity Conservation Priorities. Science, 313, 58–61. 10.1126/science.1127609 16825561

[ece370235-bib-0019] Brown, J. L. , Bennett, J. R. , & French, C. M. (2017). SDMtoolbox: the next generation python‐based GIS toolkit for landscape genetic, biogeographic and species distribution model analyses. PeerJ, 5, e4095. 10.7717/peerj.4095 29230356 PMC5721907

[ece370235-bib-0020] Burgman, M. A. , & Fox, J. C. (2003). Bias in species range estimates from minimum convex polygons: implications for conservation and options for improved planning. Animal Conservation, 6, 19–28. 10.1017/S1367943003003044

[ece370235-bib-0021] Campos, Z. , Llobet, A. , Magnusson, W. E. , & Piña, C. (2020). Caiman yacare. The IUCN Red List of Threatened Species, 2020, e.T46586A3009881. 10.2305/IUCN.UK.2020-3.RLTS.T46586A3009881.en

[ece370235-bib-0022] Campos, Z. , Magnusson, W. E. , & Muniz, F. (2019). Paleosuchus trigonatus. The IUCN Red List of Threatened Species, 2019, e.T46588A3010035. 10.2305/IUCN.UK.2019-1.RLTS.T46588A3010035.en

[ece370235-bib-0023] Canty, A. , & Ripley, B. (2021). *boot*: Bootstrap R (S‐Plus) Functions. R package version 1.3–28.

[ece370235-bib-0024] Carrascal, L. M. , Aragón, P. , Palomino, D. , & Lobo, J. M. (2015). Predicting regional densities from bird occurrence data: validation and effects of species traits in a Macaronesian Island. Diversity and Distributions, 21, 1284–1294. 10.1111/ddi.12368

[ece370235-bib-0025] Cavalcante, T. , Weber, M. M. , & Barnett, A. A. (2022). Combining geospatial abundance and ecological niche models to identify high‐priority areas for conservation: The neglected role of broadscale interspecific competition. Frontiers in Ecology and Evolution, 10, 915325. 10.3389/fevo.2022.915325

[ece370235-bib-0026] Center for International Earth Science Information Network ‐ CIESIN ‐ Columbia University . (2018). Gridded Population of the World, Version 4 (GPWv4): Population Density, Revision 11. NASA Socioeconomic Data and Applications Center (SEDAC). 10.7927/H49C6VHW

[ece370235-bib-0027] Chabreck, R. H. (1966). Methods of determining the size and composition of alligator populations in Louisiana. In *Proceedings of the Annual Conference of the Southeast Game and Fish Commission*, 20, 105–112. Retrieved from: https://seafwa.org/sites/default/files/journal‐articles/CHABRECK‐105.pdf

[ece370235-bib-0028] Chamberlain, S. , Barve, V. , Mcglinn, D. , Oldoni, D. , Desmet, P. , Geffert, L. , & Ram, K. (2023). rgbif: Interface to the Global Biodiversity Information Facility API. R package version 3.7.7. https://CRAN.R‐project.org/package=rgbif.

[ece370235-bib-0029] Chatterjee, S. , & Hadi, A. S. (2006). Regression analysis by example (236). John Wiley and Sons. 10.1002/0470055464

[ece370235-bib-0030] Cobos, M. E. , Peterson, A. T. , Barve, N. , & Osorio‐Olvera, L. (2019). kuenm: an R package for detailed development of ecological niche models using Maxent. PeerJ, 7, e6281. 10.7717/peerj.6281 30755826 PMC6368831

[ece370235-bib-0031] Da Silveira, R. , Magnusson, W. E. , & Thorbjarnarson, J. B. (2008). Factors affecting the number of caimans seen during spotlight surveys in the Mamirauá Reserve, Brazilian Amazonia. Copeia, 2, 425–430. 10.1643/CE-06-035

[ece370235-bib-0032] Dallas, T. A. , & Hastings, A. (2018). Habitat suitability estimated by niche models is largely unrelated to species abundance. Global Ecology and Biogeography, 27(12), 1448–1456. 10.1111/geb.12820

[ece370235-bib-0033] Davison, A. C. , & Hinkley, D. V. (1997). Bootstrap Methods and Their Applications. Cambridge University Press.

[ece370235-bib-0034] Dormann, C. F. , Elith, J. , Bacher, S. , Buchmann, C. , Carl, G. , Carré, G. G. , Marquéz, J. R. , Gruber, B. , Lafourcade, B. , Leitão, P. J. , Münkemüller, T. , McClean, C. , Osborne, P. E. , Reineking, B. , Schröder, B. , Skidmore, A. K. , Zurell, D. , & Lautenbach, S. (2013). Collinearity: a review of methods to deal with it and a simulation study evaluating their performance. Ecography, 36, 27–46. 10.1111/j.1600-0587.2012.07348.x

[ece370235-bib-0035] Efron, B. , & Tibshirani, R. J. (1998). An Introduction to the Boostrap. Chapman & Hall/CRC. Retrieved from: https://www.hms.harvard.edu/bss/neuro/bornlab/nb204/statistics/bootstrap.pdf

[ece370235-bib-0036] Elith, J. , Phillips, S. J. , Hastie, T. , Dudík, M. , Chee, Y. E. , & Yates, C. J. (2011). A statistical explanation of MaxEnt for ecologists. Diversity and Distributions, 17(1), 43–57. 10.1111/j.1472-4642.2010.00725.x

[ece370235-bib-0037] Elsey, R. , Woodward, A. , & Balaguera‐Reina, S. A. (2019). Alligator mississippiensis. The IUCN Red List of Threatened Species, 2019, e.T46583A3009637. 10.2305/IUCN.UK.2019-2.RLTS.T46583A3009637.en

[ece370235-bib-0038] Ergueta, P. , & Pacheco, L. F. (1990). Los Crocodilios (Orden *Crocodylia*) de Bolivia [The Crocodylians (Order Crocodylia) of Bolivia]. Ecología en Bolivia, 15, 69–81. Retrieved from http://ecologiaenbolivia.com/documents/ErguetaPacheco15.pdf

[ece370235-bib-0039] ESRI [Environmental Systems Research Institute] . (2021). ArcGIS Release 10.8.2. Redlands, CA.

[ece370235-bib-0040] Fielding, A. H. , & Bell, J. F. (1997). A review of methods for the assessment of prediction errors in conservation presence/absence models. Environmental Conservation, 24(1), 38–49. https://www.jstor.org/stable/44519240

[ece370235-bib-0041] Franklin, J. (2009). Mapping species distributions. Spatial inference and prediction. Cambridge University Press. 10.1017/CBO9780511810602

[ece370235-bib-0042] Fujisaki, I. , Mazzotti, F. J. , Dorazio, R. M. , Rice, K. G. , Cherkiss, M. , & Jeffery, B. (2011). Estimating trends in alligator populations from nightlight survey data. Wetlands, 31, 147–155. 10.1007/s13157-010-0120-0

[ece370235-bib-0043] Fukuda, Y. , Saalfeld, K. , Webb, G. , Manolis, C. , & Risk, R. (2013). Standardised method of spotlight surveys for crocodiles in the tidal rivers of the Northern Territory, Australia. Northern Territory Naturalist, 24, 14–32. 10.3316/informit.208393706566585

[ece370235-bib-0044] Gillson, L. , Dawson, T. P. , Jack, S. , & McGeoch, M. A. (2013). Accommodating climate change contingencies in conservation strategy. Trends in Ecology & Evolution, 28(3), 135–142. 10.1016/j.tree.2012.10.008 23146578

[ece370235-bib-0045] Google Earth Pro v7.3.6.9345 . (2022). South America. Image Landsat/Copernicus. Available at: https://www.google.com/intl/es_ALL/earth/about/versions/#earth‐pro

[ece370235-bib-0046] Grigg, G. C. , & Kirshner, D. (2015). Biology and Evolution of Crocodylians. Cornell University Press.

[ece370235-bib-0047] Guisan, A. , Edwards, T. C. , & Hastie, T. (2002). Generalized linear and generalized additive models in studies of species distributions: setting the scene. Ecological Modelling, 157(2–3), 89–100. 10.1016/S0304-3800(02)00204-1

[ece370235-bib-0048] Guisan, A. , & Theurillat, J. P. (2000). Equilibrium modeling of alpine plant distribution: how far can we go? Phytocoenologia, 30(3/4), 353–384. 10.1127/phyto/30/2000/353

[ece370235-bib-0049] Guisan, A. , Thuiller, W. , & Zimmermman, N. E. (2017). Habitat suitability and distributions models. With applications in R. Cambridge University Press. 10.1017/9781139028271

[ece370235-bib-0050] Guisan, A. , & Zimmermann, N. E. (2000). Predictive habitat distribution models in ecology. Ecological Modelling, 135(2–3), 147–186. 10.1016/S0304-3800(00)00354-9

[ece370235-bib-0051] Hastie, T. , Tibshirani, R. , & Friedman, J. (2017). The Elements of Statistical Learning. Data Mining, Inference, and Prediction (2nd ed.). Springer. Retrieved from: https://hastie.su.domains/ElemStatLearn/

[ece370235-bib-0052] Hidasi‐Neto, J. , Bini, L. M. , Siquiera, T. , & Cianciaruso, M. V. (2020). Ecological similarity explains species abundance distribution of small mammal communities. Acta Oecologica, 102, 103502. 10.1016/j.actao.2019.103502

[ece370235-bib-0053] Hobi, M. L. , Dubinin, M. , Graham, C. H. , Coops, N. C. , Clayton, M. K. , Pidgeon, A. M. , & Radeloff, V. C. (2017). A comparison of Dynamic Habitat Indices derived from different MODIS products as predictors of avian species richness. Remote Sensing of Environment, 195, 142–152. 10.1016/j.rse.2017.04.018

[ece370235-bib-0054] Hubert, M. , & Vandervieren, E. (2008). An adjusted boxplot for Skewed Distributions. Computational Statistics and Data Analysis, 52(12), 5186–5201. 10.1016/j.csda.2007.11.008

[ece370235-bib-0055] Hutchinson, G. E. (1957). Concluding remarks. Cold Spring Harbor Symposia on Quantitative Biology, 22, 415–427. 10.1101/SQB.1957.022.01.039

[ece370235-bib-0056] Johnston, A. , Fink, D. , Reynolds, M. D. , Hochachka, W. M. , Sullivan, B. L. , Bruns, N. E. , Hallstein, E. , Merrifield, M. S. , Matsumoto, S. , & Kelling, S. (2015). Abundance models improve spatial and temporal prioritization of conservation resources. Ecological Applications, 25(7), 1749–1756. 10.1890/14-1826.1 26591443

[ece370235-bib-0057] Jones, M. , Bertola, L. D. , & Razgour, O. (2016). Predicting the effect of interspecific competition on habitat suitability for the endangered African wild dog under future climate and land cover changes. Hystrix, the Italian Journal of Mammalogy, 27(1), 1‐8. 10.4404/hystrix-27.1-11678

[ece370235-bib-0058] Karger, D. N. , Conrad, O. , Böhner, J. , Kawohl, T. , Kreft, H. , Soria‐Auza, R. W. , Zimmermann, N. E. , Linder, H. P. , & Kessler, M. (2017). Climatologies at high resolution for the earth's land surface areas. Scientific Data, 4(1), 170122. 10.1038/sdata.2017.122 28872642 PMC5584396

[ece370235-bib-0059] Kosicki, J. Z. (2020). Generalised Additive Models and Random Forest Approach as effective methods for predictive species density and functional species richness. Environmental and Ecological Statistics, 27, 273–292. 10.1007/s10651-020-00445-5

[ece370235-bib-0060] Lehner, B. , Verdin, K. , & Jarvis, A. (2008). New global hydrography derived from spaceborne elevation data. Eos, Transactions of the American Geophysical Union, 89(10), 93–94. 10.1029/2008eo100001

[ece370235-bib-0061] Lembrechts, J. J. , van den Hoogen, J. , Aalto, J. , Ashcroft, M. B. , De Frenne, P. , Kemppinen, J. , Kopecký, M. , Luoto, M. , Maclean, I. M. D. , Crowther, T. W. , Bailey, J. J. , Haesen, S. , Klinges, D. H. , Niittynen, P. , Scheffers, B. R. , Van Meerbeek, K. , Aartsma, P. , Abdalaze, O. , Abedi, M. , … Lenoir, J. (2021). Global maps of soil temperature. Global Change Biology, 28(9), 3110–3144. 10.1111/gcb.16060 PMC9303923 34967074

[ece370235-bib-0062] Liu, X. , Feng, J. , & Wang, Y. (2019). Chlorophyll a predictability and relative importance of factors governing lake phytoplankton at different timescales. Science of the Total Environment, 648, 472–480. 10.1016/j.scitotenv.2018.08.146 30121046

[ece370235-bib-0063] Lourenço‐de‐Moraes, R. , Campos, F. S. , Cabral, P. , Silva‐Soares, T. , Nobrega, Y. C. , Covre, A. C. , & França, F. G. R. (2023). Global conservation prioritization areas in three dimensions of crocodilian diversity. Scientific Reports, 13(1), 2568. 10.1038/s41598-023-28413-6 36781891 PMC9925794

[ece370235-bib-0064] Mace, G. M. , Possingham, H. P. , & Leader‐Williams, N. (2007). Prioritizing choices in conservation. In D. MacDonald & Service, K (Eds.), Key Topics in Conservation Biology (pp. 17–34). Blackwell Publishing Ltd. Retrieved from: https://www.cpsg.org/sites/cbsg.org/files/documents/prioritizing%20choices%20in%20conservation1.pdf

[ece370235-bib-0065] Maechler, M. , Rousseeuw, P. , Croux, C. , Todorov, V. , Ruckstuhl, A. , Salibian‐Barrera, M. , Verbeke, T. , Koller, M. , Conceicao, E. L. , & di Palma, M. A. (2023). robustbase: Basic Robust Statistics R package version 0.95‐1. http://CRAN.R‐project.org/package=robustbase

[ece370235-bib-0066] Magnusson, W. E. (1982). Techniques of surveying for crocodilians. In *Proceedings of the 5* ^ *th* ^ *Working Meeting of the IUCN‐SSC Crocodile Specialist Group*. Gland, Switzerland (pp. 389‐403). http://www.iucncsg.org/365_docs/attachments/protarea/5th%20‐66845796.pdf.

[ece370235-bib-0067] Magnusson, W. E. , Campos, Z. , & Muniz, F. (2019). Paleosuchus palpebrosus. The IUCN Red List of Threatened Species, 2019, e.T46587A3009946. 10.2305/IUCN.UK.2019-1.RLTS.T46587A3009946.en

[ece370235-bib-0068] Marioni, B. , Araujo, D. D. , Villamarín, F. , & Da Silveira, R. (2013). Amazonian encounters with four crocodilian species in one single night. Crocodile Specialist Group Newsletter, 32(4), 10–13. Retrieved from: http://www.iucncsg.org/365_docs/attachments/protarea/32(4‐66316b58.pdf

[ece370235-bib-0069] Marioni, B. , Magnusson, W. E. , Vogt, R. C. , & Villamarín, F. (2022). Home range and movement patterns of male dwarf caimans (*Paleosuchus palpebrosus* and *Paleosuchus trigonatus*) living in sympatry in Amazonian floodplain streams. Neotropical Biodiversity, 8(1), 156–166. 10.1080/23766808.2022.2061292

[ece370235-bib-0070] Marra, G. , & Wood, S. N. (2011). Practical variable selection for generalized additive models. Computational Statistics and Data Analysis, 55(7), 2372–2387. 10.1016/j.csda.2011.02.004

[ece370235-bib-0071] Messel, H. , Vorlicek, G. C. , Wells, A. G. , & Green, W. J. (1981). Monograph 1. Surveys of tidal river systems in the Northern Territory of Australia and their crocodile populations. The Blyth‐Cadell rivers system study and the status of Crocodylus porosus in tidal waterways of northern Australia. Pergamon Press.

[ece370235-bib-0072] Mi, C. , Huettmann, F. , Sun, R. , & Guo, Y. (2017). Combining occurrence and abundance distribution models for the conservation of the Great Bustard. PeerJ, 5, e4160. 10.7717/peerj.4160 29255652 PMC5732545

[ece370235-bib-0073] Mu, H. , Li, X. , Wen, Y. , Huang, J. , Du, P. , Su, W. , Miao, S. , & Geng, M. (2022). A global record of annual terrestrial Human Footprint dataset from 2000 to 2018. Scientific Data, 9(1), 176. 10.1038/s41597-022-01284-8 35440581 PMC9018937

[ece370235-bib-0074] Naimi, B. , Hamm, N. A. S. , Groen, T. A. , Skidmore, A. K. , & Toxopeus, A. G. (2014). Where is positional uncertainty a problem for species distribution modelling? Ecography, 37(2), 191–203. 10.1111/j.1600-0587.2013.00205.x

[ece370235-bib-0075] Oppel, S. , Meirinho, A. , Ramírez, I. , Gardner, B. , O'Connell, A. F. , Miller, P. I. , & Louzao, M. (2012). Comparison of five modelling techniques to predict the spatial distribution and abundance of seabirds. Biological Conservation, 156, 94–104. 10.1016/j.biocon.2011.11.013

[ece370235-bib-0076] Osorio‐Olvera, L. , Soberón, J. , & Falconi, M. (2019). On population abundance and niche structure. Ecography, 42, 1415–1425. 10.1111/ecog.04442

[ece370235-bib-0077] Osorio‐Olvera, L. , Yañez‐Arenas, C. , Martínez‐Meyer, E. , & Peterson, A. T. (2020). Relationships between population densities and niche‐centroid distances in North American birds. Ecology Letters, 23(3), 555–564. 10.1111/ele.13453 31944513

[ece370235-bib-0078] Pacheco, L. F. (1996). Wariness of caiman populations and its effect on abundance estimates. Journal of Herpetology, 30(1), 123–126. 10.2307/1564725

[ece370235-bib-0079] Pacheco, L. F. , & King, W. (1995). Perspectivas de la conservación de caimanes en Bolivia [Perspectives on the conservation of alligators in Bolivia]. In A. Larriera & L. M. Verdade (Eds.), La Conservación y el Manejo de caimanes y cocodrilos de América Latina, Volumen 1 (pp. 123–134). Fundación Banco Bica.

[ece370235-bib-0080] Panter, C. T. , Clegg, R. L. , Moat, J. , Bachman, S. P. , Klitgård, B. B. , & White, R. L. (2020). To clean or not to clean: Cleaning open‐source data improves extinction risk assessments for threatened plant species. Conservation Science and Practice, 2, e311. 10.1111/csp2.311

[ece370235-bib-0081] Pekel, J. F. , Cottam, A. , Gorelick, N. , & Belward, A. S. (2016). High‐resolution mapping of global surface water and its long‐term changes. Nature, 540(7633), 418–422. 10.1038/nature20584 27926733

[ece370235-bib-0082] Peterson, A. T. , Soberon, J. , Pearson, R. G. , Anderson, R. P. , Martinez‐Meyer, E. , Nakamura, M. , & Araujo, M. B. (2011). Ecological Niches and Geographic Distributions. Princeton University Press.

[ece370235-bib-0083] Phillips, S. J. , Anderson, R. P. , Dudík, M. , Schapire, R. E. , & Blair, M. E. (2017). Opening the black box: an open‐source release of Maxent. Ecography, 40(7), 887–893. (Version 3.4.0). 10.1111/ecog.03049

[ece370235-bib-0084] Phillips, S. J. , Dudík, M. , Elith, J. , Graham, C. H. , Lehmann, A. , Leathwick, J. , & Ferrier, S. (2009). Sample selection bias and presence‐only distribution models: implications for background and pseudo‐absence data. Ecological Applications, 19(1), 181–197. 10.1890/07-2153.1 19323182

[ece370235-bib-0085] Piñeiro, G. , Perelman, S. , Guerschman, J. P. , & Paruelo, J. M. (2008). How to evaluate models: observed vs. predicted or predicted vs. observed? Ecological Modelling, 216(3–4), 316–322. 10.1016/j.ecolmodel.2008.05.006

[ece370235-bib-0086] Pollock, L. J. , O'Connor, L. M. J. , Mokany, K. , Rosauer, D. F. , Talluto, M. V. , & Thuiller, W. (2020). Protecting biodiversity (in all its complexity): new models and methods. Trends in Ecology & Evolution, 35(12), 1119–1128. 10.1016/j.tree.2020.08.015 32977981

[ece370235-bib-0087] Potts, J. M. , & Elith, J. (2006). Comparing species abundance models. Ecological Modelling, 199(2), 153–163. 10.1016/j.ecolmodel.2006.05.025

[ece370235-bib-0088] Pratt, C. J. , Denley, D. , & Metaxas, A. (2022). Selection of predictor variables for species distribution models: a case study with an invasive marine bryozoan. Oecologia, 198(2), 319–336. 10.1007/s00442-022-05110-1 35080649

[ece370235-bib-0089] Pruim, R. , Kaplan, D. T. , & Horton, N. J. (2017). The mosaic package: helping students to “think with data” using R. The R Journal, 9(1), 77–102. 10.32614/rj-2017-024

[ece370235-bib-0090] Qiao, H. , Soberón, J. , & Peterson, A. T. (2015). No silver bullets in correlative ecological niche modelling: Insights from testing among many potential algorithms for niche estimation. Methods in Ecology and Evolution, 6(10), 1126–1136. 10.1111/2041-210X.12397

[ece370235-bib-0091] R Core Team . (2022). R: A language and environment for statistical computing. R Foundation for Statistical Computing. https://www.R‐project.org/

[ece370235-bib-0092] Regos, A. , Gagne, L. , Alcaraz‐Segura, D. , Honrado, J. P. , & Domínguez, J. (2019). Effects of species traits and environmental predictors on performance and transferability of ecological niche models. Scientific Reports, 9(1), 4221. 10.1038/s41598-019-40766-5 30862919 PMC6414724

[ece370235-bib-0093] Rodriguez‐Cordero, A. L. , Balaguera‐Reina, S. A. , & Densmore, L. D., III . (2019). Regional conservation priorities for crocodylians in Bolivia. Journal for Nature Conservation, 52, 125753. 10.1016/j.jnc.2019.125753

[ece370235-bib-0094] Rodriguez‐Cordero, A. L. , Balaguera‐Reina, S. A. , Morales‐Franco, J. C. , Munn, M. , & Densmore, L. D., III . (2022). Predicting habitat suitability of *Caiman yacare* and assessing the role of protected areas under current and future climate and deforestation models. Climate Risk Management, 35, 100407. 10.1016/j.crm.2022.100407

[ece370235-bib-0095] Ruete, A. , & Leynaud, G. C. (2015). Goal‐oriented evaluation of species distribution models' accuracy and precision: True Skill Statistic profile and uncertainty maps. PeerJ PrePrints, 3, e1208v1. 10.7287/peerj.preprints.1208v1

[ece370235-bib-0096] Sagarin, R. D. , Gaines, S. D. , & Gaylord, B. (2006). Moving beyond assumptions to understand abundance distributions across the ranges of species. Trends in Ecology & Evolution, 21, 524–530. 10.1016/j.tree.2006.06.008 16815588

[ece370235-bib-0097] Sai, M. , Utete, B. , Chinoitezvi1, E. , Moyo, G. H. , & Gandiwa, E. (2016). A Survey of the abundance, population structure, and distribution of Nile Crocodiles (*Crocodylus niloticus*) using day ground surveys in Sengwa Wildlife Research Area, Zimbabwe. Herpetological Conservation and Biology, 11(42), 426–433. http://www.herpconbio.org/Volume_11/Issue_3/Sai_etal_2016.pdf

[ece370235-bib-0098] Seijas, A. E. , & Chávez, C. (2000). Population status of the Orinoco crocodile (*Crocodylus intermedius*) in the Cojedes river system, Venezuela. Biological Conservation, 94(3), 353–361. 10.1016/S0006-3207(99)00184-6

[ece370235-bib-0099] Seo, S. (2006). A Review and Comparison of Methods for Detecting Outliers in Univariate Data Sets. (Unpublished master's thesis). USA: University of Pittsburg. Retrieved from: http://d‐scholarship.pitt.edu/7948/1/Seo.pdf

[ece370235-bib-0100] Synes, N. W. , & Osborne, P. E. (2011). Choice of predictor variables as a source of uncertainty in continental‐scale species distribution modelling under climate change. Global Ecology and Biogeography, 20(6), 904–914. 10.1111/j.1466-8238.2010.00635.x

[ece370235-bib-0101] Thorbjarnarson, J. , Platt, S. G. , & Khaing, S. T. (2000). A population survey of the estuarine crocodile in the Ayeyarwady Delta, Myanmar. Oryx, 34(4), 317–324. 10.1046/j.1365-3008.2000.00135.x

[ece370235-bib-0102] Thuiller, W. , Georges, D. , Gueguen, M. , Engler, R. , Breiner, F. , Lafourcade, B. , Patin, R. , & Blancheteau, H. (2024). *biomod2*: Ensemble Platform for Species Distribution Modeling. R package version 4.2‐5‐2, https://biomodhub.github.io/biomod2/

[ece370235-bib-0103] Thuiller, W. , Münkemüller, T. , Schiffers, K. H. , Georges, D. , Dullinger, S. , Eckhart, V. M. , Edwards, T. C. , Gravel, D., Jr. , Kunstler, G. , Merow, C. , Moore, K. , Piedallu, C. , Vissault, S. , Zimmermann, N. E. , Zurell, D. , & Schurr, F. M. (2014). Does probability of occurrence relate to population dynamics? Ecography, 37(12), 1155–1166. 10.1111/ecog.00836 25722536 PMC4338510

[ece370235-bib-0104] UNEP‐WCMC & IUCN . (2024). Protected Planet: The World Database on Protected Areas (WDPA). Accessed Online, August 2024, Cambridge, UK: UNEP‐WCMC and IUCN.

[ece370235-bib-0105] van den Hoogen, J. , Lembrechts, J. , SoilTemp , Nijs, I. , & Lenoir, J. (2022). Global Soil Bioclimatic variables at 30 arc second resolution (Version 2) [Data set]. Zenodo. 10.5281/zenodo.7134169

[ece370235-bib-0106] VanDerWal, J. , Shoo, L. P. , Johnson, C. N. , & Williams, S. E. (2009). Abundance and the environmental niche: Environmental suitability estimated from niche models predicts the upper limit of local abundance. The American Naturalist, 174(2), 282–291. 10.1086/600087 19519279

[ece370235-bib-0107] Veloz, S. D. (2009). Spatially autocorrelated sampling falsely inflates measures of accuracy for presence‐only niche models. Journal of Biogeography, 36(12), 2290–2299. 10.1111/j.1365-2699.2009.02174.x

[ece370235-bib-0108] Venables, W. N. , & Dichmont, C. M. (2004). GLMs, GAMs and GLMMs: an overview of theory for applications in fisheries research. Fisheries Research, 70, 319–337. 10.1016/j.fishres.2004.08.011

[ece370235-bib-0109] Venables, W. N. , & Ripley, B. D. (2002). Modern Applied Statistics with S (4th ed.). Springer.

[ece370235-bib-0110] Vollering, J. , Halvorsen, R. , Auestad, I. , & Rydgren, K. (2019). Bunching up the background betters bias in species distribution models. Ecography, 42(10), 1717–1727. 10.1111/ecog.04503

[ece370235-bib-0111] Waddle, J. H. , Brandt, L. A. , Jeffery, B. M. , & Mazzotti, F. J. (2015). Dry years decrease abundance of American alligators in the Florida Everglades. Wetlands, 35, 865–875. 10.1007/s13157-015-0677-8

[ece370235-bib-0112] Waldock, C. , Stuart‐Smith, R. D. , Albouy, C. , Cheung, W. W. L. , Edgar, G. J. , Mouillot, D. , Tjiputra, J. , & Pellissier, L. (2022). A quantitative review of abundance‐based species distribution models. Ecography, 2022(1), e05694. 10.1111/ecog.05694

[ece370235-bib-0113] Weber, M. M. , Stevens, R. D. , Diniz‐Filho, J. A. F. , & Grelle, C. E. V. (2017). Is there a correlation between abundance and environmental suitability derived from ecological niche modelling? A meta‐analysis. Ecography, 40(7), 817–828. 10.1111/ecog.02125

[ece370235-bib-0114] Welsh, A. H. , Cunningham, R. B. , Donnelly, C. F. , & Lindenmayer, D. B. (1996). Modelling the abundance of rare species: statistical models for counts with extra zeros. Ecological Modelling, 88, 297–308. 10.1016/0304-3800(95)00113-1

[ece370235-bib-0115] Wildlife Conservation Society—WCS, and Center for International Earth Science Information Network—CIESIN—Columbia University . (2005). Last of the Wild Project, Version 2, 2005 (LWP‐2): Global Human Influence Index (HII) Dataset (Geographic). NASA Socioeconomic Data and Applications Center (SEDAC). 10.7927/H4BP00QC

[ece370235-bib-0116] Wood, J. M. , Woodward, A. R. , Humphrey, S. R. , & Hines, T. C. (1985). Night counts as an index of American Alligator population trends. Wildlife Society Bulletin, 13(3), 262–273. https://www.jstor.org/stable/3782490

[ece370235-bib-0117] Wood, S. N. (2017). Generalized Additive Models. An Introduction with R. Chapman and Hall/CRC. 10.1201/9781315370279

[ece370235-bib-0118] Yañez‐Arenas, C. , Guevara, R. , Martínez‐Meyer, E. , Mandujano, S. , & Lobo, J. M. (2014). Predicting species' abundances from occurrence data: Effects of sample size and bias. Ecological Modelling, 294, 36–41. 10.1016/j.ecolmodel.2014.09.014

[ece370235-bib-0119] Yoon, S. , & Lee, W. H. (2021). Methodological analysis of bioclimatic variable selection in species distribution modeling with application to agricultural pests (*Metcalfa pruinosa* and *Spodoptera litura*). Computers and Electronics in Agriculture, 190, 106430. 10.1016/j.compag.2021.106430

[ece370235-bib-0120] Yu, H. , Jiao, Y. , & Carstensen, L. W. (2013). Performance comparison between spatial interpolation and GLM/GAM in estimating relative abundance indices through a simulation study. Fisheries Research, 147, 186–195. 10.1016/j.fishres.2013.06.002

[ece370235-bib-0121] Zarzo‐Arias, A. , Penteriani, V. , Gábor, L. , Símová, P. , Grattarola, F. , & Moudrý, V. (2022). Importance of data selection and filtering in species distribution models: A case study on the Cantabrian brown bear. Ecosphere, 13(12), e4284. 10.1002/ecs2.4284

[ece370235-bib-0122] Zizka, A. , Antunes Carvalho, F. , Calvente, A. , Baez‐Lizarazo, M. R. , Cabral, A. , Ramos Coelho, J. F. , Colli‐Silva, M. , Fantinati, M. R. , Fernandes, M. F. , Ferreira‐Araújo, T. , Lambert Moreira, F. G. , Santos, N. M. C. , Borges Santos, T. A. , dos Santos‐Costa, R. C. , Serrano, F. C. , Alves da Silva, A. P. , de Souza, S. A. , Cavalcante de Souza, P. G. , Calisto Tomaz, E. , … Antonelli, A. (2020). No one‐size‐fits‐all solution to clean GBIF. PeerJ, 8, e9916. 10.7717/peerj.9916 33062422 PMC7528811

[ece370235-bib-0123] Zucoloto, R. B. , Bomfim, G. C. , de Campos Fernandes, F. M. , Schnadelbach, A. S. , Piña, C. I. , & Verdade, L. M. (2021). Effective population size of broad‐snouted caiman (*Caiman latirostris*) in Brazil: A historical and spatial perspective. Global Ecology and Conservation, 28, e01673. 10.1016/j.gecco.2021.e01673

[ece370235-bib-0124] Zurell, D. (2020). mecofun: useful functions for macroecology and species distribution modelling version 0.5.1. University of Potsdam. https://gitup.uni‐potsdam.de/macroecology/mecofun

[ece370235-bib-0125] Zurell, D. , König, C. , Malchow, A.‐K. , Kapitza, S. , Bocedi, G. , Travis, J. , & Fandos, G. (2022). Spatially explicit models for decision‐making in animal conservation and restoration. Ecography, (4), e057787. 10.1111/ecog.05787

[ece370235-bib-0126] Zuur, A.F. , Ieno, E.N. , Walker, N. , Saveliev, A.A. , & Smith, G.M. (2009). Mixed effects models and extensions in ecology with R. Springer. 10.1007/978-0-387-87458-6.

