# Supplementary material for: Assessing abundance–suitability models to prioritize conservation areas for the dwarf caimans in South America
Source: Ecol Evol. 2024 Aug 29;14(9):e70235. doi: 10.1002/ece3.70235 (PMC11362219; doi:10.1002/ece3.70235)
Supplement: Supplementary file 1 — Appendix S1. [file ECE3-14-e70235-s003.docx]

| **Table S1.** Scientific literature reviewed to collect occurrence data and locations with relative abundance (ind/km) samples for *Paleosuchus palpebrosus.* | | |
| --- | --- | --- |
| **No.** | **Author(s)** | **Title of research** |
| 1 | Muniz, et al. (2018) | Delimitation of evolutionary units in Cuvier’s dwarf caiman, *Paleosuchus palpebrosus* (Cuvier, 1807): insights from conservation of a broadly distributed species. |
| 2 | Campos, et al. (2012) | Parental care in the dwarf caiman, *Paleosuchus palpebrosus* Cuvier, 1807 (*Reptilia: Crocodilia: Alligatoridae*). |
| 3 | Campos, et al. (2020) | Responses of crocodilians to construction of a hydro-electric dam on the Madeira River in the Brazilian Amazon. |
| 4 | Campos, et al. (2018)* | Cuvier's Smooth-fronted Caiman *Paleosuchus palpebrosus.* |
| 5 | Rezende, et al. (2019) | A caecilian (*Siphonops* sp.) as prey of a dwarf caiman *Paleosuchus palpebrosus*: an observation from Central Brazil. |
| 6 | Muniz, et al. (2015) | New Records on Occurrence of Paleosuchus in the Branco River Basin, Roraima State, Brazil. |
| 7 | Parra-Torres, et al. (2020) | Evaluation of Crocodilian Populations Along the Bita River (Vichada, Colombia). |
| 8 | Magnusson (1992)* | *Paleosuchus palpebrosus* (Cuvier) Cuvier's Dwarf Caiman. |
| 9 | Paolillo & Gorzula (1985) | *Paleosuchus palpebrosus* (Dwarf caiman). Migration. |
| 10 | Campos & Sanaiotti (2006) | *Paleosuchus palpebrosus* (Dwarf caiman). Nesting. |
| 11 | Campos, et al. (2013)* | Growth Rates of *Paleosuchus palpebrosus* at the Southern Limit of its Range. |
| 12 | Botero-Arias (2007)* (T) | Padroes de movimiento, uso de microhabitat e dieta do jacare-pagua, *Paleosuchus palpebrosus* (*Crocodilia: Alligatoridae*), em uma floresta de paleovarzea ao sul do Rio Solimoes, Amazonia Central, Brasil. |
| 13 | Medem (1967) | El género "*Paleosuchus*" en Amazonía. |
| 14 | Rebelo & Lugli (2001) | Distribution and abundance of four caiman species (*Crocodylia: Alligatoridae*) in Jaú National Park, Amazonas, Brazil |
| 15 | King & Videz-Roca (1989) | The caimans of Bolivia: A preliminary report on a CITES and Centro de Desarrollo Forestal sponsored survey of species distribution and status. |
| 16 | Parra-Torres (2017) (T) | Evaluación de la abundancia y el hábitat de *Crocodylus intermedius* y otros crocodilianos en el rio Bita (Vichada, Colombia). |
| 17 | Pacheco-Alvarez (2009) (T) | Distribución, abundancia y estructura poblacional del Babo Morichalero (*Paleosuchus palpebrosus*) en los Llanos Orientales del Estado Anzoátegui. |
| 18 | Gorzula & Paolillo (1986) | La ecologia y el estado actual de los aligatoridos de la Guyana Venezolana. |
| 19 | Seijas (2006)* | Características distintivas y estado de conocimiento de las especies del género *Paleosuchus* (*Crocodylia; Alligatoridae*) en Venezuela. |
| 20 | Moldowan et al. (2016) | Diet, gastric parasitism, and injuries of Caimans (*Caiman*, *Melanosuchus*, and *Paleosuchus*) in the Peruvian Amazon. |
| 21 | Carvalho & Véras (2013) | Distribution and abundance of *Caiman latirostris* and *Paleosuchus palpebrosus* at Grande Sertao Veredas National Park, Central Brazil. |
| 22 | Campos et al. (2015) | Geographic Variation in Clutch Size and Reproductive Season of the Dwarf Caiman, *Paleosuchus palpebrosus*, in Brazil. |
| 23 | Marioni et al. (2022) | Home range and movement patterns of male dwarf caimans (*Paleosuchus palpebrosus* and *Paleosuchus trigonatus*) living in sympatry in Amazonian floodplain streams. |
| 24 | Seijas (2011) | Los Crocodylia de Venezuela: Ecología y Conservación. |
| 25 | Campos et al. (2010) | Maximum size of dwarf caiman, *Paleosuchus palpebrosus* (Cuvier, 1807), in the Amazon and habitats surrounding the Pantanal, Brazil. |
| 26 | Rebelo (2002) (T) | Quelonios, Jacarés e Ribeirinhos no Parque Nacional do Jau (AM) |
| 27 | Campos (2007) | Registro de Ocorrencia de Jacaré-pagua (*Paleosuchus palpebrosus*), na RPPN Engenheiro Eliezer Batista, Pantanal, Brasil. |
| 28 | Ali et al. (2016) | The discovery of Cuvier's Dwarf Caiman, *Paleosuchus palpebrosus* (Reptilia: Alligatoridae) in Trinidad. |
| 29 | Campos & Magnusson (2013) | Thermal relations of dwarf caiman, *Paleosuchus palpebrosus*, in a hillshade stream: Evidence for an unusual thermal niche among crocodilians. |
| 30 | Pacheco (1993) (T) | Abundance, distribution and habitat use of Crocodilians in Beni, Bolivia. |
| 31 | Medem (1983) | Los Crocodylia de Sur America Volumen II. |
| 32 | Muniz et al. (2019) | Detecting population structure *Paleosuchus trigonatus* (*Alligatoridae: Caimaninae*) through microsatellites markers developed by next generation sequencing. |
| 33 | Souza (2010) (T) | Ocorrencia, uso de habitats e distribuicao de jacares (*Alligatoridae*) na estacao ecologica de Maraca, Roraima, Amazonia Brasileira. |
| 34 | Scott et al. (1990) | Distribution, habitats, and conservation of the caimans (*Alligatoridae*) of Paraguay |
| 35 | Godshalk (2006) (T) | Phylogeography and conservation genetics of the yacare caiman (*Caiman yacare*) of South America. |
| 36 | Morais et al. (2010) | First record of *Amblyomma rotundatum* Koch, 1844 (Acari: Ixodidae) parasitizing *Paleosuchus palpebrosus* Cuvier, 1807 (Reptilia:Crocodylidae), in the western border of Pantanal, Mato Grosso do Sul, Brazil. |
| 37 | Bustillo (2009) (T) | Population density, distribution patterns and conservation of *Melanosuchus niger* and *Caiman crocodilus* (*Crocodylia, Alligatoridae*) in the lakes of Puerto Nariño, Amazonas, Colombia. |
| 38 | Campos et al. (2022) | Temperature variation in nests of *Paleosuchus palpebrosus* (*Crocodylia: Alligatoridae*) near the southern edge of the species´ range, Brazil. |
| 39 | Campos et al. (2022)* | Geographic variation in colour and spot patterns in Dwarf Caiman, *Paleosuchus palpebrosus* (Cuvier, 1807) in Brazil. |
| 40 | Campos & Magnusson (2016)* | Density and biomass estimates by removal for an amazonian crocodilian, *Paleosuchus palpebrosus.* |
| 41 | Villamarín et al. (2018) | Body size is more important than diet in determining stable-isotope estimates of trophic position in crocodilians. |
| 42 | Marioni et al. (2013) | Amazonian encounters with four Crocodilian species in one single night. |
| 43 | Magnusson et al. (2019) | *Paleosuchus palpebrosus*. The IUCN Red List of Threatened Species 2019. |

* Documents with distribution and/or abundance estimations information, but with high uncertainty or coordinates (latitude and longitude) not reported.

(T) Thesis

| **Table S2.** Scientific literature reviewed to collect occurrence data and locations with relative abundance (ind/km) samples for *Paleosuchus trigonatus.* | | |
| --- | --- | --- |
| **No.** | **Author(s)** | **Title of research** |
| 1 | Campos et al. (2017) | Extension of the geographical distribution of Schneider’s Dwarf Caiman, *Paleosuchus trigonatus* (Schneider, 1801) (*Crocodylia: Alligatoridae*), in the Amazon–Cerrado transition, Brazil. |
| 2 | Magnusson & Lima (1991)* | The Ecology of a Cryptic Predator, *Paleosuchus trigonatus*, in a Tropical Rainforest. |
| 3 | Tejado (2012) | Abundancia de *Melanosuchus niger* Spix, 1828, *Paleosuchus trigonatus* Schneider, 1801 y *Caiman crocodilus* L., 1758 en el cauce medio del río Mazán (Amazonas, Perú). |
| 4 | Campos et al. (2019) | Asynchronous reproduction in three species of crocodilians in south-eastern Amazonia. |
| 5 | Campos et al. (2020) | Responses of crocodilians to construction of a hydro-electric dam on the Madeira River in the Brazilian Amazon. |
| 6 | Souza (2010) (T) | Ocorrencia, uso de habitats e distribuicao de jacares (*Alligatoridae*) na estacao ecologica de Maraca, Roraima, Amazonia Brasileira. |
| 7 | Venegas‐Anaya et al. (2008) | Mitochondrial DNA Phylogeography of *Caiman crocodilus* in Mesoamerica and South America |
| 8 | Muniz et al. (2019) | Detecting population structure *Paleosuchus trigonatus* (*Alligatoridae: Caimaninae*) through microsatellites markers developed by next generation sequencing. |
| 9 | Ávila et al. (2011) | Herpetofauna of São João da Barra Hydroelectric Plant, state of Mato Grosso, Brazil. |
| 10 | Gorzula et al. (1989) | Applications of Limnology and Hydrology to Crocodilian Conservation and Management |
| 11 | Muniz et al. (2015) | New Records on Occurrence of Paleosuchus in the Branco River Basin, Roraima State, Brazil |
| 12 | Zilca et al. (2013)* | Avaliação do risco de extinção do jacaré-coroa *Paleosuchus trigonatus* (Schneider, 1801) no Brasil |
| 13 | Marquis et al. (2020) | Observations on breeding site, bioacoustics and biometry of hatchlings of *Paleosuchus trigonatus* (Schneider, 1801) from French Guiana (*Crocodylis_Alligatoridae*) |
| 14 | Bittencourt et al. (2019) | Evidence of cryptic lineages within a small South American crocodilian: the Schneider's dwarf caiman *Paleosuchus trigonatus* (*Alligatoridae: Caimaninae*). |
| 15 | de Assis & Dos Santos (2007) | *Paleosuchus trigonatus*. Predation. |
| 16 | Magnusson & Campos (2010)* | Schneider's Smooth-fronted Caiman *Paleosuchus trigonatus.* |
| 17 | Valeris et al. (2014) | Primer registro de anidación de *Paleosuchus trigonatu*s (*Crocodylia: Alligatoridae*) para Venezuela. |
| 18 | Valeris, C. (2016) (T) | Estado poblacional, distribución geoespacial, aspectos reproductivos y uso consuntivo de *Paleosuchus trigonatus* (*Crocodylia: Alligatoridae*) en la Cuenca del Río Erebato, Alto Caura, Venezuela. |
| 19 | Medem (1967) | El género "Paleosuchus" en Amazonía. |
| 20 | Rebelo & Lugli (2001) | Distribution and abundance of four caiman species (*Crocodylia: Alligatoridae*) in Jaú National Park, Amazonas, Brazil. |
| 21 | King & Godshalk (1996) | A Program for the Sustainable Utilization and Management of Caimans: A Report to the government of Bolivia on the final results of the 1995 and 1996 field seasons. |
| 22 | King & Videz-Roca (1989) | The caimans of Bolivia: A preliminary report on a CITES and Centro de Desarrollo Forestal sponsored survey of species distribution and status. |
| 23 | Gorzula & Paolillo (1986) | La Ecología y estado actual de los Alligatoridos de la Guyana Venezolana. |
| 24 | Moldowan et al. (2016) | Diet, gastric parasitism, and injuries of Caimans (*Caiman*, *Melanosuchus*, and *Paleosuchus*) in the Peruvian Amazon. |
| 25 | Marioni et al. (2022) | Home range and movement patterns of male dwarf caimans (*Paleosuchus palpebrosus* and *Paleosuchus trigonatus*) living in sympatry in Amazonian floodplain streams. |
| 26 | Seijas (2011) | Los Crocodylia de Venezuela: Ecología y Conservación. |
| 27 | Rebelo (2002) (T) | Quelonios, Jacarés e Ribeirinhos no Parque Nacional do Jau (AM). |
| 28 | Medem (1983) | Los Crocodylia de Sur America Volumen II. |
| 29 | Villamarín et al. (2018) | Body size is more important than diet in determining stable-isotope estimates of trophic position in crocodilians. |
| 30 | Marioni et al. (2013) | Amazonian encounters with four Crocodilian species in one single night. |
| 31 | Barao-Nóbrega et al. (2020) | *Paleosuchus trigonatus* (Schneider's smooth-fronted caiman). Hatchling dispersal. |
| 32 | Magnusson et al. (2022)* | *Paleosuchus trigonatus* (Schneider, 1801). |
| 33 | Lugo et al. (2013) | Biología y Conservación de los Crocodylia de Colombia. 11. *Paleosuchus trigonatus* (Schneider 1801). |
| 34 | Campos et al. (2019) | *Paleosuchus palpebrosus*. The IUCN Red List of Threatened Species 2019. |

* Documents with distribution and/or abundance estimations information, but with high uncertainty or coordinates (latitude and longitude) not reported.

(T) Thesis

| **Table S3.** Ecological predictors included in the model the structure to estimate the potential distribution and abundance of *Paleosuchus palpebrosus* and *P. trigonatus*. | | |
| --- | --- | --- |
| **Predictor^a^** | **Variant** | **Abbreviation^b^** |
| Soil Temperature (ST)**^c^** (*https://zenodo.org/record/7134169#.Y_1cn3bMJhE*) | Annual mean temperature (0 - 5 cm.) | st_a_bio_01 |
|  | Mean diurnal range (0 - 5 cm.) | st_a_bio_02 |
|  | Isothermality (0 - 5 cm.) | st_a_bio_03 |
|  | Temperature seasonality (0 - 5 cm.) | st_a_bio_04 |
|  | Maximum temperature of the warmest month (0 - 5 cm.) | st_a_bio_05 |
|  | Minimum temperature of the coldest month (0 - 5 cm.) | st_a_bio_06 |
|  | Temperature annual range (0 - 5 cm.) | st_a_bio_07 |
|  | Mean temperature of the wettest quarter (0 - 5 cm.) | st_a_bio_08 |
|  | Mean temperature of the driest quarter (0 - 5 cm.) | st_a_bio_09 |
|  | Mean temperature of the warmest quarter (0 - 5 cm.) | st_a_bio_10 |
|  | Mean temperature of the coldest quarter (0 - 5 cm.) | st_a_bio_11 |
|  | Annual mean temperature (5 - 15 cm.) | st_b_bio_01 |
|  | Mean diurnal range (5 - 15 cm.) | st_b_bio_02 |
|  | Isothermality (5 - 15 cm.) | st_b_bio_03 |
|  | Temperature seasonality (5 - 15 cm.) | st_b_bio_04 |
|  | Maximum temperature of the warmest month (5 - 15 cm.) | st_b_bio_05 |
|  | Minimum temperature of the coldest month (5 - 15 cm.) | st_b_bio_06 |
|  | Temperature annual range (5 - 15 cm.) | st_b_bio_07 |
|  | Mean temperature of the wettest quarter (5 - 15 cm.) | st_b_bio_08 |
|  | Mean temperature of the driest quarter (5 - 15 cm.) | st_b_bio_09 |
|  | Mean temperature of the warmest quarter (5 - 15 cm.) | st_b_bio_10 |
|  | Mean temperature of the coldest quarter (5 - 15 cm.) | st_b_bio_11 |
| Dynamic Habitat Indices (DHI) (*http://silvis.forest.wisc.edu/data/dhis/*) | Enhanced vegetation index (EVI) - cumulative | dhi_evi_cum |
|  | Enhanced vegetation Index (EVI) - minimum | dhi_evi_min |
|  | Enhanced vegetation Index (EVI) - seasonality | dhi_evi_sea |
|  | Fraction absorbed photosynthetically active radiation (FPAR) - cumulative | dhi_fpar_cum |
|  | Fraction absorbed photosynthetically active radiation (FPAR) - minimum | dhi_fpar_min |
|  | Fraction absorbed photosynthetically active radiation (FPAR) - seasonality | dhi_fpar_sea |
|  | Gross primary Productivity (GPP) - cumulative | dhi_gpp_cum |
|  | Gross primary Productivity (GPP) - minimum | dhi_gpp_min |
|  | Gross primary Productivity (GPP) - seasonality | dhi_gpp_sea |
|  | Leaf area Index (LAI) - cumulative | dhi_lai_cum |
|  | Leaf area Index (LAI)- minimum | dhi_lai_min |
|  | Leaf area Index (LAI) - seasonality | dhi_lai_sea |
|  | Normalized difference vegetation index (NDVI) - cumulative | dhi_ndvi_cum |
|  | Normalized difference vegetation index (NDVI) - minimum | dhi_ndvi_min |
|  | Normalized difference vegetation index (NDVI) - seasonality | dhi_ndvi_sea |
| Temperature (T) (*http://chelsa-climate.org*) | Mean annual air temperature | bio_01 |
|  | Mean diurnal air temperature range | bio_02 |
|  | Isothermality | bio_03 |
|  | Temperature seasonality | bio_04 |
|  | Mean daily maximum air temperature of the warmest month | bio_05 |
|  | Mean daily minimum air temperature of the coldest month | bio_06 |
|  | Annual range of air temperature | bio_07 |
|  | Mean daily mean air temperatures of the wettest quarter | bio_08 |
|  | Mean daily mean air temperatures of the driest quarter | bio_09 |
|  | Mean daily mean air temperatures of the warmest quarter | bio_10 |
|  | Mean daily mean air temperatures of the coldest quarter | bio_11 |
| Precipitation (P) (*http://chelsa-climate.org*) | Annual precipitation amount | bio_12 |
|  | Precipitation amount of the wettest month | bio_13 |
|  | Precipitation amount of the driest month | bio_14 |
|  | Precipitation seasonality | bio_15 |
|  | Mean monthly precipitation amount of the wettest quarter | bio_16 |
|  | Mean monthly precipitation amount of the driest quarter | bio_17 |
|  | Mean monthly precipitation amount of the warmest quarter | bio_18 |
|  | Mean monthly precipitation amount of the coldest quarter | bio_19 |
| Topography (To) (*https://www.hydrosheds.org/downloads*; *https://www.earthenv.org/topography*) | Elevation | t_dem |
|  | Flow accumulation | t_flow_acc |
|  | Slope | t_slope |
|  | Roughness | t_rough |
|  | Topographic position index (TPI) | t_tpi |
|  | Terrain ruggedness Index (TRI) | t_tri |
|  | Vector ruggedness measure (VRM) | t_vrm |
|  |  |  |
|  |  |  |
| **Table S3.** Continued | | |
| **Predictor (source)** | **Variant** | **Abbreviation** |
| Human (H) (*https://sedac.ciesin.columbia.edu/data/set/wildareas-v2-human-influence-index-geographic; https://sedac.ciesin.columbia.edu/data/set/gpw-v4-population-density-rev11; https://doi.org/10.6084/m9.figshare.16571064*) | Global human influence index (GHII) | h_hii |
|  | Population density (PD) | h_pd |
|  | Human footprint (HF) | h_hfp |
| Water (W) (*https://global-surface-water.appspot.com/download*) | Global surface water occurrence | w_occurr |
|  | Global surface water recurrence | w_recurr |
|  | Global surface water seasonality | w_season |

**^a^** Type of predictor included in the modeling analysis and estimations. Each type of predictor includes its online source.

**^b^** Abbreviation of ecological predictor variants used in this study.

**^c^** For each Soil Temperature variant, two depth intervals were included (0 – 5 cm. and 5 – 15 cm.)

| **Table S4.** Uncorrelated ecological predictor variants that were used to estimate the environmental heterogeneity within the study area extent of *Paleosuchus palpebrosus* and *Paleosuchus trigonatus.* | | | | |
| --- | --- | --- | --- | --- |
| **Species** | **Predictor** | **Variant** | **Abbreviation** | **VIF^*^** |
| *Paleosuchus palpebrosus* | Soil Temperature | Isothermality (0 - 5 cm.) | sa_bio_03 | 1.003 |
|  |  | Temperature seasonality (0 - 5 cm.) | sa_bio_04 | 3.01 |
|  |  | Mean temperature of the wettest quarter (0 - 5 cm.) | sa_bio_08 | 5.442 |
|  |  | Isothermality (5 - 15 cm.) | sb_bio_03 | 1.004 |
|  |  | Mean temperature of the driest quarter (5 - 15 cm.) | sb_bio_09 | 3.687 |
|  | Dynamic Habitat Indices | Enhanced vegetation Index (EVI) - seasonality | dhi_evi_sea | 2.29 |
|  |  | Gross primary Productivity (GPP) - seasonality | dhi_gpp_sea | 2.944 |
|  |  | Leaf area Index (LAI)- minimum | dhi_lai_min | 2.429 |
|  |  | Normalized difference vegetation index (NDVI) - minimum | dhi_ndvi_min | 2.184 |
|  | Temperature | Isothermality | bio_03 | 2.279 |
|  | Precipitation | Precipitation amount of the wettest month | bio_13 | 2.009 |
|  |  | Precipitation amount of the driest month | bio_14 | 4.098 |
|  |  | Mean monthly precipitation amount of the warmest quarter | bio_18 | 4.341 |
|  | Topography | Elevation | t_dem | 3.815 |
|  |  | Flow accumulation | t_flow_acc | 1.028 |
|  |  | Topographic position index (TPI) | t_tpi | 1.035 |
|  |  | Vector ruggedness measure (VRM) | t_vrm | 1.226 |
|  | Human | Population density (PD) | h_pd | 1.636 |
|  |  | Human footprint (HF) | h_hfp | 1.050 |
|  | Water | Global surface water recurrence | w_recurr | 1.129 |
| *Paleosuchus*  *trigonatus* | Soil Temperature | Mean diurnal range (0 - 5 cm.) | sa_bio_02 | 3.561 |
|  |  | Isothermality (0 - 5 cm.) | sa_bio_03 | 1.002 |
|  |  | Isothermality (5 - 15 cm.) | sb_bio_03 | 3.087 |
|  |  | Temperature seasonality (5 - 15 cm.) | sb_bio_04 | 2.666 |
|  |  | Minimum temperature of the coldest month (5 - 15 cm.) | sb_bio_06 | 4.825 |
|  |  | Temperature annual range (5 - 15 cm.) | sb_bio_07 | 3.659 |
|  |  | Mean temperature of the wettest quarter (5 - 15 cm.) | sb_bio_08 | 4.511 |
|  | Dynamic Habitat Indices | Enhanced vegetation Index (EVI)- minimum | dhi_evi_min | 3.059 |
|  |  | Enhanced vegetation Index (EVI) - seasonality | dhi_evi_sea | 2.004 |
|  |  | Gross primary Productivity (GPP) - minimum | dhi_gpp_min | 3.622 |
|  |  | Gross primary Productivity (GPP) - seasonality | dhi_gpp_sea | 2.477 |
|  |  | Leaf area Index (LAI)- minimum | dhi_lai_min | 2.708 |
|  |  | Leaf area Index (LAI) - seasonality | dhi_lai_sea | 2.728 |
|  | Temperature | Isothermality | bio_03 | 4.539 |
|  |  | Mean daily maximum air temperature of the warmest month | bio_05 | 10.295 |
|  | Precipitation | Precipitation amount of the wettest month | bio_13 | 2.111 |
|  |  | Precipitation amount of the driest month | bio_14 | 2.353 |
|  |  | Mean monthly precipitation amount of the coldest quarter | bio_19 | 3.213 |
|  | Topography | Flow accumulation | t_flow_acc | 1.032 |
|  |  | Topographic position index (TPI) | t_tpi | 1.038 |
|  |  | Vector ruggedness measure (VRM) | t_vrm | 1.438 |
|  | Human | Global human influence index (GHII) | h_hii | 1.682 |
|  |  | Population density (PD) | h_pd | 1.058 |
|  |  | Human footprint (HF) | h_hfp | 1.966 |
|  | Water | Global surface water seasonality | w_season | 1.275 |

**^*^** Variation Inflation Factor

| **Table S5.** Evaluation parameters for each of the model structures and specifications defined for *Paleosuchus palpebrosus*. We detailed the code to fit the model in Rstudio. | | | | | | | | | | | | | | | |
| --- | --- | --- | --- | --- | --- | --- | --- | --- | --- | --- | --- | --- | --- | --- | --- |
|  |  |  | **No. Predictors** | | |  |  |  | **Evaluation parameter** | | | | | | |
| **Set** | **Algo.** |  | **Init.** | **Unco.** | **Mod.** |  | **Code to fit model** |  | **AIC** | **AUC** | **TSS** | **Kappa** | **Sens.** | **Spec.** | **Occ.** |
| **S1** | GLM |  | 3 | 2 | 2 |  | glm(P_palpebrosus ~ w_recurr + w_season, family = "binomial", data = Pp_m8_BI_dis_10405_2_pred) |  | 3315.1 | 0.543 | 0.114 | 0.132 | 0.138 | 0.976 | 0.943 |
|  | GLMsel |  | 3 | 2 | 1 |  | glm(P_palpebrosus ~ w_recurr , family = "binomial", data = Pp_m8_sel_BI_dis_10405_2_pred) |  | 3314.9 | 0.551 | 0.114 | 0.132 | 0.138 | 0.976 | 0.943 |
|  | GAM |  | 3 | 2 | 2 |  | gam(P_palpebrosus ~ s(w_recurr,k=4) + s(w_season, k=4), family="binomial", data = Pp_m8_GAM_dis_10405_2_pred, method = "REML") |  | 3315.5 | 0.543 | 0.114 | 0.132 | 0.138 | 0.976 | 0.944 |
|  | GAMsel |  | 3 | 2 | 1 |  | gam(P_palpebrosus ~ s(w_recurr, k=4), family="binomial", data = Pp_m8_sel_GAM_dis_10405_2_pred, method = "REML", select = TRUE) |  | 3315 | 0.541 | 0.113 | 0.143 | 0.131 | 0.982 | 0.949 |
| **S2** | GLM |  | 3 | 2 | 2 |  | glm(P_palpebrosus ~ h_hii + h_pd, family = "binomial", data = Pp_m5_BI_dis_10405_2_pred) |  | 3376.4 | 0.585 | 0.083 | 0.007 | 0.968 | 0.115 | 0.148 |
|  | GLMsel |  | 3 | 2 | 2 |  | glm(P_palpebrosus ~ h_hii + h_pd, family="binomial", data = Pp_m5_sel_BI_dis_10405_2_pred) |  | 3376.4 | 0.585 | 0.083 | 0.007 | 0.968 | 0.115 | 0.148 |
|  | GAM |  | 3 | 2 | 2 |  | gam(P_palpebrosus ~ s(h_hii,k=4) + s(h_pd, k=4), family="binomial", data = Pp_m5_GAM_dis_10405_2_pred, method = "REML") |  | 3370.6 | 0.579 | 0.135 | 0.013 | 0.951 | 0.184 | 0.214 |
|  | GAMsel |  | 3 | 2 | 2 |  | gam(P_palpebrosus ~ s(h_hii,k=4) + s(h_pd, k=4), family="binomial", data = Pp_m5_sel_GAM_dis_10405_2_pred, method = "REML", select = TRUE) |  | 3376.5 | 0.585 | 0.083 | 0.007 | 0.968 | 0.115 | 0.148 |
| **S3** | GLM |  | 7 | 4 | 4 |  | glm(P_palpebrosus ~ t_dem + t_flow_acc + t_tpi + t_vrm, family="binomial", data = Pp_m7_BI_dis_10405_4_pred) |  | 3252.5 | 0.695 | 0.303 | 0.049 | 0.756 | 0.548 | 0.556 |
|  | GLMsel |  | 7 | 4 | 2 |  | glm(P_palpebrosus ~ t_dem + t_flow_acc, family="binomial", data = Pp_m7_sel_BI_dis_10405_4_pred) |  | 3250.6 | 0.695 | 0.312 | 0.049 | 0.768 | 0.544 | 0.552 |
|  | GAM |  | 7 | 4 | 4 |  | gam(P_palpebrosus ~ s(t_dem,k=4) + s(t_flow_acc, k=4) + s(t_tpi, k=4) + s(t_vrm, k=4), family="binomial", data = Pp_m7_GAM_dis_10405_4_pred, method = "REML") |  | 3230.7 | 0.695 | 0.293 | 0.04 | 0.832 | 0.461 | 0.475 |
|  | GAMsel |  | 7 | 4 | 2 |  | gam(P_palpebrosus ~ s(t_dem,k=4) + s(t_flow_acc, k=4), family="binomial", data = Pp_m7_sel_GAM_dis_10405_4_pred, method = "REML", select = TRUE) |  | 3226.2 | 0.696 | 0.301 | 0.041 | 0.844 | 0.456 | 0.471 |
| **S4** | GLM |  | 8 | 3 | 3 |  | glm(P_palpebrosus ~ bio_13 + bio_15 + bio_18 , family="binomial", data = Pp_m3_BI_dis_10405_3_pred) |  | 3385.4 | 0.58 | 0.137 | 0.105 | 0.195 | 0.942 | 0.913 |
|  | GLMsel |  | 8 | 3 | 3 |  | glm(P_palpebrosus ~ bio_13 + bio_15 + bio_18, family="binomial", data = Pp_m3_sel_BI_dis_10405_3_pred) |  | 3385.4 | 0.58 | 0.137 | 0.105 | 0.195 | 0.942 | 0.913 |
|  | GAM |  | 8 | 3 | 3 |  | gam(P_palpebrosus ~ s(bio_13,k=4) + s(bio_15, k=4) + s(bio_18, k=4), family="binomial", data = Pp_m3_GAM_dis_10405_3_pred, method = "REML") |  | 3308.2 | 0.649 | 0.197 | 0.032 | 0.642 | 0.555 | 0.559 |
|  | GAMsel |  | 8 | 3 | 3 |  | gam(P_palpebrosus ~ s(bio_13,k=4) + s(bio_15, k=4) + s(bio_18, k=4), family="binomial", data = Pp_m3_sel_GAM_dis_10405_3_pred, method = "REML", select = TRUE) |  | 3309.8 | 0.646 | 0.205 | 0.033 | 0.657 | 0.548 | 0.553 |
| **S5** | GLM |  | 11 | 5 | 5 |  | glm(P_palpebrosus ~ bio_03 + bio_04 + bio_05 + bio_08 + bio_09, family="binomial", data = Pp_m2_BI_dis_10405_5_pred) |  | 3218.3 | 0.725 | 0.353 | 0.099 | 0.588 | 0.766 | 0.759 |
|  | GLMsel |  | 11 | 5 | 5 |  | glm(P_palpebrosus ~ bio_03 + bio_04 + bio_05 + bio_08 + bio_09 , family="binomial", data = Pp_m2_sel_BI_dis_10405_5_pred) |  | 3218.3 | 0.725 | 0.353 | 0.099 | 0.588 | 0.766 | 0.759 |
|  | GAM |  | 11 | 5 | 5 |  | gam(P_palpebrosus ~ s(bio_03,k=4) + s(bio_04, k=4) + s(bio_05, k=4) + s(bio_08, k=4) + s(bio_09, k=4), family="binomial", data = Pp_m2_GAM_dis_10405_5_pred, method = "REML") |  | 3101.4 | 0.761 | 0.428 | 0.096 | 0.731 | 0.697 | 0.699 |
|  | GAMsel |  | 11 | 5 | 5 |  | gam(P_palpebrosus ~ s(bio_03,k=4) + s(bio_04, k=4) + s(bio_05, k=4) + s(bio_08, k=4) + s(bio_09, k=4), family="binomial", data = Pp_m2_sel_GAM_dis_10405_5_pred, method = "REML", select = TRUE) |  | 3103.5 | 0.761 | 0.431 | 0.095 | 0.741 | 0.69 | 0.692 |
| **S6** | GLM |  | 15 | 4 | 4 |  | glm(P_palpebrosus ~ dhi_evi_sea + dhi_gpp_sea + dhi_lai_min + dhi_ndvi_min, family="binomial", data = Pp_m4_BI_dis_10405_4_pred) |  | 3363.3 | 0.585 | 0.106 | 0.038 | 0.284 | 0.822 | 0.801 |
|  | GLMsel |  | 15 | 4 | 3 |  | glm(P_palpebrosus ~ dhi_gpp_sea + dhi_lai_min + dhi_ndvi_min, family="binomial", data = Pp_m4_sel_BI_dis_10405_4_pred) |  | 3362.4 | 0.589 | 0.111 | 0.04 | 0.289 | 0.823 | 0.802 |
|  | GAM |  | 15 | 4 | 4 |  | gam(P_palpebrosus ~ s(dhi_evi_sea,k=4) + s(dhi_gpp_sea, k=4) + s(dhi_lai_min, k=4) + s(dhi_ndvi_min, k=4), family="binomial", data = Pp_m4_GAM_dis_10405_4_pred, method = "REML") |  | 3288.8 | 0.624 | 0.165 | 0.032 | 0.533 | 0.631 | 0.627 |
|  | GAMsel |  | 15 | 4 | 4 |  | gam(P_palpebrosus ~ s(dhi_evi_sea, k=4) + s(dhi_gpp_sea, k=4) + s(dhi_lai_min, k=4) + s(dhi_ndvi_min, k=4),family="binomial", data = Pp_m4_sel_GAM_dis_10405_4_pred, method ="REML",select=TRUE) |  | 3305.3 | 0.617 | 0.163 | 0.059 | 0.341 | 0.822 | 0.804 |
| **S7** | GLM |  | 22 | 5 | 5 |  | glm(P_palpebrosus ~ sa_bio_03 + sb_bio_02 + sb_bio_03 + sb_bio_04 + sb_bio_09, family="binomial", data = Pp_m6_BI_dis_10405_5_pred) |  | 3387.6 | 0.608 | 0.205 | 0.042 | 0.551 | 0.655 | 0.651 |
|  | GLMsel |  | 22 | 5 | 5 |  | glm(P_palpebrosus ~ sa_bio_03 + sb_bio_02 + sb_bio_03 + sb_bio_04 + sb_bio_09, family="binomial", data = Pp_m6_sel_BI_dis_10405_5_pred) |  | 3387.6 | 0.608 | 0.205 | 0.042 | 0.551 | 0.655 | 0.651 |
|  | GAM |  | 22 | 5 | 5 |  | gam(P_palpebrosus ~ s(sa_bio_03,k=4) + s(sb_bio_02, k=4) + s(sb_bio_03, k=4) + s(sb_bio_04, k=4) + s(sb_bio_09, k=4), family="binomial", data = Pp_m6_GAM_dis_10405_5_pred, method = "REML") |  | 3247.4 | 0.701 | 0.316 | 0.058 | 0.701 | 0.615 | 0.618 |
|  | GAMsel |  | 22 | 5 | 5 |  | gam(P_palpebrosus~s(sa_bio_03,k=4)+ s(sb_bio_02, k=4)+s(sb_bio_03,k=4)+s(sb_bio_04,k=4) +s(sb_bio_09, k=4), family="binomial",data = Pp_m6_sel_GAM_dis_10405_5_pred, method="REML",select=TRUE) |  | 3248 | 0.695 | 0.312 | 0.057 | 0.701 | 0.61 | 0.614 |
|  |  |  |  |  |  |  |  |  |  |  |  |  |  |  |  |
|  |  |  |  |  |  |  |  |  |  |  |  |  |  |  |  |
|  |  |  |  |  |  |  |  |  |  |  |  |  |  |  |  |
|  |  |  |  |  |  |  |  |  |  |  |  |  |  |  |  |
| **Table S5. Continued.** | | | | | | | | | | | | | | | |
| **S8** | GLM |  | 22 | 9 | 9 |  | glm(P_palpebrosus ~ bio_03 + bio_04 + bio_05 + bio_08 + bio_09 + bio_13 + bio_14 + bio_18 + w_season, family="binomial", data = Pp_m9_BI_dis_10405_9_pred) |  | 3128 | 0.74 | 0.382 | 0.164 | 0.514 | 0.868 | 0.854 |
|  | GLMsel |  | 22 | 9 | 7 |  | glm(P_palpebrosus ~ bio_03 + bio_04 + bio_08 + bio_09 + bio_13 + bio_14 + w_season, family="binomial", data = Pp_m9_sel_BI_dis_10405_9_pred) |  | 3124.6 | 0.743 | 0.379 | 0.162 | 0.511 | 0.867 | 0.854 |
|  | GAM |  | 22 | 9 | 9 |  | gam(P_palpebrosus~s(bio_03,k=4)+s(bio_04,k=4)+s(bio_05,k=4)+s(bio_08, k=4) + s(bio_09, k=4) + s(bio_13,k=4) + s(bio_14,k=4) + s(bio_18,k=4) + s(w_season,k=4), family="binomial",data=Pp_m9_GAM_dis_10405_9_pred, method = "REML") |  | 2972.4 | 0.775 | 0.431 | 0.109 | 0.691 | 0.739 | 0.738 |
|  | GAMsel |  | 22 | 9 | 9 |  | gam(P_palpebrosus ~ s(bio_03,k=4) + s(bio_04, k=4) + s(bio_05, k=4) + s(bio_08, k=4) + s(bio_09, k=4) + s(bio_13, k=4) + s(bio_14, k=4) + s(bio_18, k=4) + s(w_season, k=4), family="binomial", data = Pp_m9_sel_GAM_dis_10405_9_pred, method = "REML", select = TRUE) |  | 2971.2 | 0.774 | 0.429 | 0.137 | 0.625 | 0.804 | 0.797 |
| **S9** | GLM |  | 25 | 11 | 11 |  | glm(P_palpebrosus ~ bio_03 + bio_04 + bio_05 + bio_08 + bio_09 + bio_13 + bio_14 + bio_18 + h_hii + h_pd + w_season, family = "binomial", data = Pp_m10_BI_dis_10405_11_pred) |  | 3058.9 | 0.755 | 0.401 | 0.094 | 0.689 | 0.713 | 0.712 |
|  | GLMsel |  | 25 | 11 | 9 |  | glm(P_palpebrosus ~ bio_03 + bio_04 + bio_08 + bio_09 + bio_13 + bio_14 + h_hii + h_pd + w_season, family = "binomial", data = Pp_m10_sel_BI_dis_10405_11_pred) |  | 3055.1 | 0.757 | 0.398 | 0.095 | 0.681 | 0.717 | 0.715 |
|  | GAM |  | 25 | 11 | 11 |  | gam(P_palpebrosus ~ s(bio_03,k=4) + s(bio_04, k=4) + s(bio_05, k=4) + s(bio_08, k=4) + s(bio_09, k=4) + s(bio_13, k=4) + s(bio_14, k=4) + s(bio_18, k=4) + s(h_hii, k=4) + s(h_pd, k=4) + s(w_season, k=4), family="binomial", data = Pp_m10_GAM_dis_10405_11_pred, method = "REML") |  | 2906 | 0.781 | 0.431 | 0.14 | 0.622 | 0.809 | 0.802 |
|  | GAMsel |  | 25 | 11 | 10 |  | gam(P_palpebrosus ~ s(bio_03,k=4) + s(bio_04, k=4) + s(bio_05, k=4) + s(bio_08, k=4) + s(bio_09, k=4) + s(bio_13, k=4) + s(bio_14, k=4) + s(bio_18, k=4) + s(h_hii, k=4) + s(w_season, k=4), family="binomial", data = Pp_m10_sel_GAM_dis_10405_11_pred, method = "REML", select = TRUE) |  | 2909.4 | 0.781 | 0.436 | 0.112 | 0.694 | 0.742 | 0.74 |
| **S10** | GLM |  | 32 | 15 | 15 |  | glm(P_palpebrosus ~ bio_03 + bio_04 + bio_05 + bio_08 + bio_09 + bio_13 + bio_14 + bio_18 + h_hii + h_pd + t_dem + t_flow_acc + t_tpi + t_vrm + w_season, family="binomial", data = Pp_m11_BI_dis_10405_15_pred) |  | 3025.3 | 0.766 | 0.43 | 0.097 | 0.731 | 0.699 | 0.7 |
|  | GLMsel |  | 32 | 15 | 11 |  | glm(P_palpebrosus ~ bio_03 + bio_04 + bio_05 + bio_09 + bio_13 + bio_14 + h_hii + h_pd + t_dem + t_vrm + w_season, family="binomial", data = Pp_m11_sel_BI_dis_10405_15_pred) |  | 3020.4 | 0.767 | 0.422 | 0.094 | 0.726 | 0.696 | 0.697 |
|  | GAM |  | 32 | 15 | 15 |  | gam(P_palpebrosus ~ s(bio_03,k=4)+s(bio_04, k=4)+s(bio_05, k=4)+s(bio_08,k=4) + s(bio_09, k=4) +s(bio_13, k=4)+s(bio_14, k=4)+s(bio_18, k=4)+s(h_hii,k=4)+s(h_pd, k=4) + s(t_dem, k=4) + s(t_flow_acc, k=4) + s(t_tpi, k=4)+s(t_vrm, k=4)+s(w_season, k=4), family="binomial", data = Pp_m11_GAM_dis_10405_15_pred, method = "REML") |  | 2856.7 | 0.801 | 0.487 | 0.153 | 0.684 | 0.803 | 0.798 |
|  | GAMsel |  | 32 | 15 | 9 |  | gam(P_palpebrosus ~ s(bio_03,k=4) + s(bio_04, k=4) + s(bio_05, k=4) + s(bio_09, k=4) + s(bio_13, k=4) + s(bio_14, k=4) + s(h_hii,k=4) + s(t_dem, k=4) + s(w_season, k=4), family="binomial", data = Pp_m11_sel_GAM_dis_10405_15_pred, method = "REML", select = TRUE) |  | 2853.7 | 0.806 | 0.51 | 0.159 | 0.709 | 0.802 | 0.798 |
| **S11** | GLM |  | 47 | 19 | 19 |  | glm(P_palpebrosus ~ bio_03 + bio_04 + bio_05 + bio_08 + bio_09 + bio_13 + bio_14 + bio_18 + dhi_evi_sea + dhi_gpp_sea + dhi_lai_min + dhi_ndvi_min + h_hii + h_pd + t_dem + t_flow_acc + t_tpi + t_vrm + w_recurr, family="binomial", data = Pp_m12_BI_dis_10405_19_pred) |  | 3016.4 | 0.768 | 0.436 | 0.101 | 0.726 | 0.71 | 0.71 |
|  | GLMsel |  | 47 | 19 | 13 |  | glm(P_palpebrosus ~ bio_03 + bio_04 + bio_05 + bio_09 + bio_13 + bio_14 + dhi_evi_sea + dhi_gpp_sea + h_hii + h_pd + t_dem + t_vrm + w_recurr, family="binomial", data = Pp_m12_sel_BI_dis_10405_19_pred) |  | 3008.5 | 0.771 | 0.439 | 0.101 | 0.733 | 0.706 | 0.707 |
|  | GAM |  | 47 | 19 | 19 |  | gam(P_palpebrosus ~ s(bio_03,k=4) + s(bio_04, k=4) + s(bio_05, k=4) + s(bio_08, k=4) + s(bio_09, k=4) + s(bio_13, k=4) + s(bio_14, k=4) + s(bio_18, k=4) + s(dhi_evi_sea,k=4) + s(dhi_gpp_sea, k=4) + s(dhi_lai_min, k=4) + s(dhi_ndvi_min, k=4) + s(h_hii,k=4) + s(h_pd, k=4) + s(t_dem, k=4) + s(t_flow_acc, k=4) + s(t_tpi, k=4) + s(t_vrm, k=4) + s(w_recurr, k=4), family="binomial", data = Pp_m12_GAM_dis_10405_19_pred, method = "REML") |  | 2831.1 | 0.803 | 0.494 | 0.127 | 0.748 | 0.746 | 0.746 |
|  | GAMsel |  | 47 | 19 | 11 |  | gam(P_palpebrosus ~ s(bio_03,k=4) + s(bio_05, k=4) + s(bio_09, k=4) + s(bio_13, k=4) + s(bio_14, k=4) + s(dhi_evi_sea,k=4) + s(dhi_gpp_sea, k=4) + s(h_hii,k=4) + s(t_dem, k=4) + s(t_flow_acc, k=4) + s(w_recurr, k=4), family="binomial", data = Pp_m12_sel_GAM_dis_10405_19_pred, method = "REML", select = TRUE) |  | 2830.1 | 0.807 | 0.503 | 0.127 | 0.763 | 0.74 | 0.741 |
| **S12** | GLM |  | 69 | 20 | 20 |  | glm(P_palpebrosus ~ bio_03 + bio_13 + bio_14 + bio_18 + dhi_evi_sea + dhi_gpp_sea + dhi_lai_min + dhi_ndvi_min + h_hfp + h_pd + sa_bio_03 + sa_bio_04 + sa_bio_08 + sb_bio_03 + sb_bio_09 + t_dem + t_flow_acc + t_tpi + t_vrm + w_recurr, family="binomial", data = Pp_1_10405_20_pred) |  | 2973.7 | 0.791 | 0.481 | 0.113 | 0.765 | 0.716 | 0.718 |
|  | GLMsel |  | 69 | 20 | 12 |  | glm(P_palpebrosus ~ bio_03 + bio_13 + bio_14 + bio_18 + dhi_evi_sea + dhi_gpp_sea + dhi_lai_min + dhi_ndvi_min + h_hfp + h_pd + sa_bio_03 + sa_bio_04 + sa_bio_08 + sb_bio_03 + sb_bio_09 + t_dem + t_flow_acc + t_tpi + t_vrm + w_recurr, family="binomial", data = Pp_m1_sel_BI_dis_10405_20_pred) |  | 2963.5 | 0.792 | 0.478 | 0.111 | 0.765 | 0.712 | 0.714 |
|  | GAM |  | 69 | 20 | 20 |  | gam(P_palpebrosus ~ s(bio_03,k=4) + s(bio_13, k=4) + s(bio_14, k=4) + s(bio_18, k=4) + s(dhi_evi_sea, k=4) + s(dhi_gpp_sea, k=4) + s(dhi_lai_min, k=4) + s(dhi_ndvi_min, k=4) + s(h_hfp, k=4) + s(h_pd, k=4) + s(sa_bio_03, k=4) + s(sa_bio_04, k=4) + s(sa_bio_08, k=4) + s(sb_bio_03, k=4) + s(sb_bio_09, k=4) + s(t_dem, k=4) + s(t_flow_acc, k=4) + s(t_tpi, k=4) + s(t_vrm, k=4) + s(w_recurr, k=4), family="binomial", data = Pp_m1_GAM_dis_10405_20_pred, method = "REML") |  | 2798.6 | 0.819 | 0.516 | 0.138 | 0.758 | 0.758 | 0.758 |
|  | GAMsel |  | 69 | 20 | 10 |  | gam(P_palpebrosus ~ s(bio_03,k=4) + s(bio_13, k=4) + s(bio_14, k=4) + s(dhi_evi_sea, k=4) + s(dhi_gpp_sea, k=4) + s(h_hfp, k=4) + s(sa_bio_04, k=4) + s(sb_bio_03, k=4) + s(t_dem, k=4) + s(w_recurr, k=4), family="binomial", data = Pp_m1_sel_GAM_dis_10405_20_pred, method = "REML", select = TRUE) |  | 2792.2 | 0.821 | 0.535 | 0.173 | 0.723 | 0.812 | 0.808 |
|  |  |  |  |  |  |  |  |  |  |  |  |  |  |  |  |
| **Table S5. Continued** | | | | | | | | | | | | | | | |
| **S13** | GLM |  | 69(25) | 22 | 22 |  | glm(P_palpebrosus ~ bio_03 + bio_05 + bio_08 + bio_13 + bio_15 + bio_18 + dhi_evi_sea + dhi_gpp_sea + dhi_lai_min + dhi_ndvi_min + h_hii + h_pd + sa_bio_03 + sb_bio_02 + sb_bio_03 + sb_bio_04 + sb_bio_09 + t_dem + t_flow_acc + t_tpi + t_vrm + w_recurr, family="binomial", data = Pp_m13_BI_dis_10405_22_pred) |  | 2989.2 | 0.783 | 0.456 | 0.104 | 0.751 | 0.705 | 0.707 |
|  | GLMsel |  | 69(25) | 22 | 13 |  | glm(P_palpebrosus ~ bio_03 + bio_05 + bio_13 + bio_15 + dhi_evi_sea + dhi_gpp_sea + h_hii + h_pd + sa_bio_03 + sb_bio_02 + sb_bio_04 + t_dem + w_recurr, family="binomial", data = Pp_m13_sel_BI_dis_10405_22_pred) |  | 2977.9 | 0.786 | 0.455 | 0.104 | 0.751 | 0.705 | 0.707 |
|  | GAM |  | 69(25) | 22 | 22 |  | gam(P_palpebrosus ~ s(bio_03,k=4) + s(bio_05, k=4) + s(bio_08, k=4) + s(bio_13, k=4) + s(bio_15, k=4) + s(bio_18,k=4) + s(dhi_evi_sea, k=4) + s(dhi_gpp_sea, k=4) + s(dhi_lai_min, k=4) + s(dhi_ndvi_min, k=4) + s(h_hii,k=4) + s(h_pd, k=4) + s(sa_bio_03, k=4) + s(sb_bio_02, k=4) + s(sb_bio_03, k=4) + s(sb_bio_04, k=4) + s(sb_bio_09, k=4) + s(t_dem, k=4) + s(t_flow_acc, k=4) + s(t_tpi, k=4) + s(t_vrm, k=4) + s(w_recurr, k=4), family="binomial", data = Pp_m13_GAM_dis_10405_22_pred, method = "REML") |  | 2710 | 0.830 | 0.548 | 0.155 | 0.773 | 0.776 | 0.775 |
|  | GAMsel |  | 69(25) | 22 | 15 |  | gam(P_palpebrosus ~ s(bio_03,k=4) + s(bio_05, k=4) + s(bio_13, k=4) + s(bio_15, k=4) + s(bio_18,k=4) + s(dhi_evi_sea, k=4) + s(dhi_gpp_sea, k=4) + s(h_hii,k=4) + s(sb_bio_02, k=4) + s(sb_bio_03, k=4) + s(sb_bio_04, k=4) + s(sb_bio_09, k=4) + s(t_dem, k=4) + s(t_flow_acc, k=4) + s(w_recurr, k=4), family="binomial", data = Pp_m13_sel_GAM_dis_10405_22_pred, method = "REML", select = TRUE) |  | 2697.9 | 0.828 | 0.536 | 0.174 | 0.723 | 0.813 | 0.809 |

**Set:** Set of predictors containing a different number of variants.

**Algo.:** Algorithm used to change model specification.

**Init.:** Number of initial predictor variants within the predictor set.

**Unco.:** Number of uncorrelated predictor variants selected based on Pearson correlation coefficient (>0.7) and Variation Inflation Factor (VIF).

**Mod.:** Number of predictor variants included in model calibration.

**AIC:** Akaike Information Criteria.

**AUC:** Area under the receiver operator characteristics curve.

**TSS:** True skill statistics.

**Sens.:** Sensitivity. Proportion of true presences classified correctly (as presence).

**Spec.:** Specificity. Proportion of true absences (pseudo-absences) classified correctly (as absence).

**Occ.:** Observation correctly classified. Proportion of both presences and absences (pseudo-absences) classified correctly.

| **Table S6.** Evaluation statistics distribution models’ structures and specifications defined for *Paleosuchus trigonatus*. We detailed the code to fit the model in Rstudio. | | | | | | | | | | | | | | | |
| --- | --- | --- | --- | --- | --- | --- | --- | --- | --- | --- | --- | --- | --- | --- | --- |
|  |  |  | **No. Predictors** | | |  |  |  | **Evaluation parameter** | | | | | | |
| **Set** | **Algo.** |  | **Init.** | **Unco.** | **Mod.** |  | **Code to fit model** |  | **AIC** | **AUC** | **TSS** | **Kappa** | **Sens.** | **Spec.** | **Occ.** |
| **S1** | GLM |  | 3 | 2 | 2 |  | glm(P_trigonatus ~ w_recurr + w_season, family="binomial", data = Pt_m8_BI_dis_10386_2_pred) |  | 3225.8 | 0.543 | 0.102 | 0.114 | 0.127 | 0.975 | 0.943 |
|  | GLMsel |  | 3 | 2 | 1 |  | glm(P_trigonatus ~ w_recurr, family="binomial", data = Pt_m8_sel_BI_dis_10386_2_pred) |  | 3223.9 | 0.543 | 0.102 | 0.114 | 0.127 | 0.975 | 0.943 |
|  | GAM |  | 3 | 2 | 2 |  | gam(P_trigonatus ~ s(w_recurr, k=4) + s(w_season, k=4), family="binomial", data = Pt_m8_GAM_dis_10386_2_pred, method = "REML") |  | 3225.8 | 0.543 | 0.102 | 0.114 | 0.127 | 0.975 | 0.943 |
|  | GAMsel |  | 3 | 2 | 1 |  | gam(P_trigonatus~s(w_recurr,k=4),family="binomial",data= Pt_m8_sel_GAM_dis_10386_2_pred, method = "REML", select = TRUE) |  | 3223.9 | 0.543 | 0.102 | 0.114 | 0.127 | 0.975 | 0.943 |
| **S2** | GLM |  | 3 | 3 | 3 |  | glm(P_trigonatus ~ h_hfp + h_hii + h_pd, family="binomial", data = Pt_m5_BI_dis_10386_3_pred) |  | 3068.5 | 0.742 | 0.381 | 0.075 | 0.72 | 0.661 | 0.663 |
|  | GLMsel |  | 3 | 3 | 2 |  | glm(P_trigonatus ~ h_hfp + h_hii, family="binomial", data = Pt_m5_sel_BI_dis_10386_3_pred) |  | 3067 | 0.745 | 0.386 | 0.075 | 0.728 | 0.658 | 0.661 |
|  | GAM |  | 3 | 3 | 3 |  | gam(P_trigonatus ~ s(h_hfp, k=4) + s(h_hii, k=4) + s(h_pd, k=4), family="binomial", data = Pt_m5_GAM_dis_10386_3_pred, method = "REML") |  | 3033.4 | 0.739 | 0.364 | 0.074 | 0.692 | 0.673 | 0.673 |
|  | GAMsel |  | 3 | 3 | 2 |  | gam(P_trigonatus ~ s(h_hfp, k=4) + s(h_hii, k=4), family="binomial", data = Pt_m5_sel_GAM_dis_10386_3_pred, method = "REML", select = TRUE) |  | 3032.4 | 0.744 | 0.369 | 0.076 | 0.692 | 0.677 | 0.678 |
| **S3** | GLM |  | 7 | 4 | 4 |  | glm(P_trigonatus~t_dem+t_flow_acc+t_tpi+t_vrm, family= "binomial", data=Pt_m7_BI_dis_10386_4_pred) |  | 3293.9 | 0.554 | 0.071 | 0.041 | 0.163 | 0.908 | 0.88 |
|  | GLMsel |  | 7 | 4 | 2 |  | glm(P_trigonatus ~ t_flow_acc + t_tpi, family = "binomial", data = Pt_m7_sel_BI_dis_10386_4_pred) |  | 3290.5 | 0.566 | 0.058 | 0.038 | 0.132 | 0.926 | 0.896 |
|  | GAM |  | 7 | 4 | 4 |  | gam(P_trigonatus ~ s(t_dem, k=4) + s(t_flow_acc, k=4) + s(t_tpi, k=4) + s(t_vrm, k=4), family="binomial", data = Pt_m7_GAM_dis_10386_4_pred, method = "REML") |  | 3280.8 | 0.569 | 0.083 | 0.053 | 0.161 | 0.923 | 0.894 |
|  | GAMsel |  | 7 | 4 | 2 |  | gam(P_trigonatus ~ s(t_flow_acc, k=4) + s(t_tpi, k=4), family="binomial", data = Pt_m7_sel_GAM_dis_10386_4_pred, method = "REML", select = TRUE) |  | 3277.3 | 0.584 | 0.06 | 0.048 | 0.114 | 0.946 | 0.915 |
| **S4** | GLM |  | 8 | 3 | 3 |  | glm(P_trigonatus ~ bio_13 + bio_14 + bio_19, family="binomial", data = Pt_m3_BI_dis_10386_3_pred) |  | 3285.2 | 0.558 | 0.07 | 0.015 | 0.386 | 0.684 | 0.672 |
|  | GLMsel |  | 8 | 3 | 3 |  | glm(P_trigonatus ~ bio_13 + bio_14 + bio_19, family="binomial", data = Pt_m3_sel_BI_dis_10386_3_pred) |  | 3285.2 | 0.558 | 0.07 | 0.015 | 0.386 | 0.684 | 0.672 |
|  | GAM |  | 8 | 3 | 3 |  | gam(P_trigonatus ~ s(bio_13,k=4) + s(bio_14, k=4) + s(bio_19, k=4), family="binomial", data = Pt_m3_GAM_dis_10386_3_pred, method = "REML") |  | 3278.5 | 0.552 | 0.077 | 0.051 | 0.15 | 0.927 | 0.898 |
|  | GAMsel |  | 8 | 3 | 3 |  | gam(P_trigonatus ~ s(bio_13,k=4) + s(bio_14, k=4) + s(bio_19, k=4), family="binomial", data = Pt_m3_sel_GAM_dis_10386_3_pred, method = "REML", select = TRUE) |  | 3281.6 | 0.552 | 0.062 | 0.048 | 0.119 | 0.943 | 0.912 |
| **S5** | GLM |  | 11 | 5 | 5 |  | glm(P_trigonatus ~ bio_02 + bio_04 + bio_05 + bio_08 + bio_09, family="binomial", data = Pt_m2_BI_dis_10386_5_pred) |  | 3201.5 | 0.649 | 0.212 | 0.026 | 0.782 | 0.429 | 0.443 |
|  | GLMsel |  | 11 | 5 | 5 |  | glm(P_trigonatus ~ bio_02 + bio_04 + bio_05 + bio_08 + bio_09, family="binomial", data = Pt_m2_sel_BI_dis_10386_5_pred) |  | 3201.5 | 0.649 | 0.212 | 0.026 | 0.782 | 0.429 | 0.443 |
|  | GAM |  | 11 | 5 | 5 |  | gam(P_trigonatus ~ s(bio_02,k=4) + s(bio_04, k=4) + s(bio_05, k=4) + s(bio_08, k=4) + s(bio_09, k=4), family="binomial", data = Pt_m2_GAM_dis_10386_5_pred, method = "REML") |  | 3197.1 | 0.654 | 0.236 | 0.03 | 0.798 | 0.439 | 0.452 |
|  | GAMsel |  | 11 | 5 | 4 |  | gam(P_trigonatus ~ s(bio_02,k=4) + s(bio_05, k=4) + s(bio_08, k=4) + s(bio_09, k=4), family="binomial", data = Pt_m2_sel_GAM_dis_10386_5_pred, method = "REML", select = TRUE) |  | 3194.9 | 0.654 | 0.215 | 0.027 | 0.785 | 0.43 | 0.443 |
| **S6** | GLM |  | 15 | 6 | 6 |  | glm(P_trigonatus ~ dhi_evi_min + dhi_evi_sea + dhi_gpp_min + dhi_gpp_sea + dhi_lai_min + dhi_lai_sea, family="binomial", data = Pt_m4_BI_dis_10386_6_pred) |  | 3255.5 | 0.577 | 0.115 | 0.028 | 0.389 | 0.726 | 0.714 |
|  | GLMsel |  | 15 | 6 | 3 |  | glm(P_trigonatus ~ dhi_evi_min + dhi_gpp_sea + dhi_lai_min, family="binomial", data = Pt_m4_sel_BI_dis_10386_6_pred) |  | 3250 | 0.581 | 0.113 | 0.056 | 0.225 | 0.887 | 0.863 |
|  | GAM |  | 15 | 6 | 6 |  | gam(P_trigonatus~s(dhi_evi_min,k=4)+s(dhi_evi_sea,k=4)+s(dhi_gpp_min,k=4)+s(dhi_gpp_sea,k=4)+ s(dhi_lai_min, k=4)+s(dhi_lai_sea, k=4),family="binomial", data = Pt_m4_GAM_dis_10386_6_pred, method = "REML") |  | 3232.7 | 0.605 | 0.145 | 0.035 | 0.417 | 0.728 | 0.716 |
|  | GAMsel |  | 15 | 6 | 4 |  | gam(P_trigonatus ~ s(dhi_evi_min,k=4) + s(dhi_gpp_min, k=4) + s(dhi_gpp_sea,k=4) + s(dhi_lai_min, k=4), family="binomial", data = Pt_m4_sel_GAM_dis_10386_6_pred, method = "REML", select = TRUE) |  | 3237.3 | 0.579 | 0.109 | 0.033 | 0.321 | 0.788 | 0.771 |
| **S7** | GLM |  | 22 | 7 | 7 |  | glm(P_trigonatus ~ sa_bio_02 + sa_bio_03 + sb_bio_03 + sb_bio_04 + sb_bio_06 + sb_bio_07 + sb_bio_08, family="binomial", data = Pt_m6_BI_dis_10386_7_pred) |  | 3214.6 | 0.63 | 0.186 | 0.06 | 0.378 | 0.808 | 0.792 |
|  | GLMsel |  | 22 | 7 | 5 |  | glm(P_trigonatus ~ sb_bio_03 + sb_bio_04 + sb_bio_06 + sb_bio_07 + sb_bio_08, family="binomial", data = Pt_m6_sel_BI_dis_10386_7_pred) |  | 3211 | 0.632 | 0.194 | 0.063 | 0.386 | 0.808 | 0.792 |
|  | GAM |  | 22 | 7 | 7 |  | gam(P_trigonatus~s(sa_bio_02,k=4)+s(sa_bio_03,k=4)+s(sb_bio_03,k=4)+s(sb_bio_04, k=4)+ s(sb_bio_06, k=4)+s(sb_bio_07,k=4)+s(sb_bio_08,k=4),family="binomial",data = Pt_m6_GAM_dis_10386_7_pred, method = "REML") |  | 3105.8 | 0.692 | 0.272 | 0.077 | 0.495 | 0.777 | 0.766 |
|  | GAMsel |  | 22 | 7 | 6 |  | gam(P_trigonatus ~ s(sa_bio_02,k=4) + s(sb_bio_03, k=4) + s(sb_bio_04, k=4) + s(sb_bio_06, k=4) +s(sb_bio_07, k=4) + s(sb_bio_08, k=4), family="binomial", data = Pt_m6_sel_GAM_dis_10386_7_pred, method = "REML", select = TRUE) |  | 3100.3 | 0.691 | 0.268 | 0.075 | 0.492 | 0.775 | 0.765 |
|  |  |  |  |  |  |  |  |  |  |  |  |  |  |  |  |
|  |  |  |  |  |  |  |  |  |  |  |  |  |  |  |  |
|  |  |  |  |  |  |  |  |  |  |  |  |  |  |  |  |
|  |  |  |  |  |  |  |  |  |  |  |  |  |  |  |  |
|  |  |  |  |  |  |  |  |  |  |  |  |  |  |  |  |
|  |  |  |  |  |  |  |  |  |  |  |  |  |  |  |  |
|  | | | | | | | | | | | | | | | |
| **Table S6. Continued** | | | | | | | | | | | | | | | |
| **S8** | GLM |  | 22 | 9 | 9 |  | glm(P_trigonatus ~ bio_02 + bio_04 + bio_05 + bio_08 + bio_09 + bio_13 + bio_14 + bio_19 + w_season, family="binomial", data = Pt_m9_BI_dis_10386_9_pred) |  | 3135.8 | 0.668 | 0.243 | 0.063 | 0.495 | 0.749 | 0.739 |
|  | GLMsel |  | 22 | 9 | 9 |  | glm(P_trigonatus ~ bio_02 + bio_04 + bio_05 + bio_08 + bio_09 + bio_13 + bio_14 + bio_19 + w_season, family="binomial", data = Pt_m9_sel_BI_dis_10386_9_pred) |  | 3135.8 | 0.668 | 0.243 | 0.063 | 0.495 | 0.749 | 0.739 |
|  | GAM |  | 22 | 9 | 9 |  | gam(P_trigonatus ~ s(bio_02,k=4) + s(bio_04, k=4) + s(bio_05, k=4) + s(bio_08, k=4) + s(bio_09, k=4) + s(bio_13, k=4) + s(bio_14, k=4) + s(bio_19, k=4) + s(w_season, k=4), family="binomial", data = Pt_m9_GAM_dis_10386_9_pred, method = "REML") |  | 3120.1 | 0.657 | 0.224 | 0.116 | 0.324 | 0.9 | 0.878 |
|  | GAMsel |  | 22 | 9 | 8 |  | gam(P_trigonatus~s(bio_02,k=4)+s(bio_04,k=4)+s(bio_05,k=4)+s(bio_09,k=4)+s(bio_13,k=4)+s(bio_14 ,k=4)+s(bio_19,k=4)+s(w_season,k=4), family = "binomial", data = Pt_m9_sel_GAM_dis_10386_9_pred, method="REML",select=TRUE) |  | 3122.5 | 0.656 | 0.215 | 0.087 | 0.358 | 0.857 | 0.839 |
| **S9** | GLM |  | 25 | 12 | 12 |  | glm(P_trigonatus ~ bio_02 + bio_04 + bio_05 + bio_08 + bio_09 + bio_13 + bio_14 + bio_19 + h_hfp + h_hii + h_pd + w_season, family="binomial", data = Pt_m10_BI_dis_10386_12_pred) |  | 2929 | 0.775 | 0.412 | 0.079 | 0.762 | 0.65 | 0.655 |
|  | GLMsel |  | 25 | 12 | 10 |  | glm(P_trigonatus ~ bio_02 + bio_04 + bio_05 + bio_09 + bio_13 + bio_14 + bio_19 + h_hfp + h_hii + w_season, family="binomial", data = Pt_m10_sel_BI_dis_10386_12_pred) |  | 2925.6 | 0.778 | 0.418 | 0.08 | 0.769 | 0.649 | 0.653 |
|  | GAM |  | 25 | 12 | 12 |  | gam(P_trigonatus ~ s(bio_02,k=4) + s(bio_04, k=4) + s(bio_05, k=4) + s(bio_08, k=4) + s(bio_09, k=4) + s(bio_13, k=4) + s(bio_14, k=4) + s(bio_19, k=4) + s(h_hfp,k=4) + s(h_hii,k=4) + s(h_pd, k=4) + s(w_season, k=4), family="binomial", data = Pt_m10_GAM_dis_10386_12_pred, method = "REML") |  | 2845.2 | 0.787 | 0.452 | 0.129 | 0.666 | 0.786 | 0.782 |
|  | GAMsel |  | 25 | 12 | 11 |  | gam(P_trigonatus ~ s(bio_02,k=4) + s(bio_04, k=4) + s(bio_05, k=4) + s(bio_08, k=4) + s(bio_09, k=4) + s(bio_13, k=4) + s(bio_14, k=4) + s(bio_19, k=4) + s(h_hfp,k=4) + s(h_hii,k=4) + s(w_season, k=4), family="binomial", data = Pt_m10_sel_GAM_dis_10386_12_pred, method = "REML", select = TRUE) |  | 2844.5 | 0.793 | 0.455 | 0.132 | 0.663 | 0.792 | 0.787 |
| **S10** | GLM |  | 32 | 15 | 15 |  | glm(P_trigonatus ~ bio_02 + bio_04 + bio_05 + bio_08 + bio_09 + bio_13 + bio_14 + bio_19 + h_hfp + h_hii + h_pd + t_flow_acc + t_tpi + t_vrm + w_season, family="binomial", data = Pt_m11_BI_dis_10386_15_pred) |  | 2930.1 | 0.772 | 0.417 | 0.08 | 0.764 | 0.653 | 0.657 |
|  | GLMsel |  | 32 | 15 | 11 |  | glm(P_trigonatus ~ bio_02 + bio_04 + bio_05 + bio_09 + bio_13 + bio_14 + bio_19 + h_hfp + h_hii + t_tpi + w_season, family="binomial", data = Pt_m11_sel_BI_dis_10386_15_pred) |  | 2923.9 | 0.778 | 0.417 | 0.08 | 0.767 | 0.65 | 0.655 |
|  | GAM |  | 32 | 15 | 15 |  | gam(P_trigonatus ~ s(bio_02,k=4)+s(bio_04, k=4)+s(bio_05, k=4)+s(bio_08, k=4)+s(bio_09, k=4) + s(bio_13, k=4)+s(bio_14, k=4)+s(bio_19, k=4)+s(h_hfp,k=4)+s(h_hii,k=4)+s(h_pd, k=4)+s(t_flow_acc,k=4) + s(t_tpi, k=4) + s(t_vrm, k=4) + s(w_season, k=4), family="binomial", data = Pt_m11_GAM_dis_10386_15_pred, method = "REML") |  | 2842.7 | 7.88E-01 | 0.456 | 0.132 | 0.666 | 0.79 | 0.785 |
|  | GAMsel |  | 32 | 15 | 13 |  | gam(P_trigonatus~s(bio_02,k=4)+s(bio_04,k=4)+s(bio_05,k=4)+s(bio_08,k=4)+s(bio_09, k=4) + s(bio_13, k=4) + s(bio_14, k=4) + s(bio_19, k=4) + s(h_hfp,k=4) + s(h_hii,k=4) + s(t_flow_acc, k=4) + s(t_tpi, k=4) + s(w_season, k=4), family="binomial", data = Pt_m11_sel_GAM_dis_10386_15_pred, method = "REML", select = TRUE) |  | 2839.9 | 7.94E-01 | 0.457 | 0.097 | 0.764 | 0.692 | 0.695 |
| **S11** | GLM |  | 47 | 20 | 20 |  | glm(P_trigonatus ~ bio_02 + bio_04 + bio_09 + bio_13 + bio_14 + bio_19 + dhi_evi_min + dhi_evi_sea + dhi_gpp_min + dhi_gpp_sea + dhi_lai_min + dhi_lai_sea + h_hfp + h_hii + h_pd + t_dem + t_flow_acc + t_tpi + t_vrm + w_recurr, family="binomial", data = Pt_m12_BI_dis_10386_20_pred) |  | 2916.6 | 0.761 | 0.405 | 0.131 | 0.588 | 0.817 | 0.808 |
|  | GLMsel |  | 47 | 20 | 13 |  | glm(P_trigonatus~bio_02+bio_04+bio_09+bio_13+bio_14 + bio_19 + dhi_evi_min + dhi_gpp_min + dhi_lai_sea + h_hfp + h_hii + t_tpi + w_recurr, family="binomial", data = Pt_m12_sel_BI_dis_10386_20_pred) |  | 2906.1 | 0.764 | 0.413 | 0.134 | 0.596 | 0.817 | 0.809 |
|  | GAM |  | 47 | 20 | 20 |  | gam(P_trigonatus ~ s(bio_02,k=4)+s(bio_04, k=4)+s(bio_09, k=4)+s(bio_13, k=4)+s(bio_14, k=4) + s(bio_19, k=4)+s(dhi_evi_min, k=4)+s(dhi_evi_sea,k=4)+s(dhi_gpp_min, k=4)+s(dhi_gpp_sea, k=4)+s(dhi_lai_min,k=4) + s(dhi_lai_sea, k=4) + s(h_hfp,k=4) + s(h_hii,k=4) + s(h_pd, k=4) + s(t_dem, k=4) + s(t_flow_acc, k=4) + s(t_tpi, k=4) + s(t_vrm, k=4) + s(w_recurr, k=4), family="binomial", data = Pt_m12_GAM_dis_10386_20_pred, method = "REML") |  | 2843.6 | 7.85E-01 | 0.461 | 0.103 | 0.751 | 0.71 | 0.712 |
|  | GAMsel |  | 47 | 20 | 13 |  | gam(P_trigonatus~s(bio_02,k=4)+s(bio_04,k=4)+s(bio_09,k=4)+s(bio_13, k=4)+s(bio_14, k=4) + s(bio_19,k=4)+s(dhi_evi_min,k=4)+s(dhi_gpp_min,k=4)+s(dhi_gpp_sea,k=4)+s(h_hfp,k=4)+s(h_hii,k=4)+s(t_flow_acc, k=4) +s(w_recurr, k=4),family="binomial",data = Pt_m12_sel_GAM_dis_10386_20_pred,method="REML",select=TRUE) |  | 2838.5 | 7.89E-01 | 0.448 | 0.099 | 0.741 | 0.707 | 0.708 |
| **S12** | GLM |  | 69 | 25 | 25 |  | glm(P_trigonatus~bio_03+bio_05+bio_13+bio_14+bio_19+dhi_evi_min+dhi_evi_sea+dhi_gpp_min + dhi_gpp_sea+dhi_lai_min+dhi_lai_sea+h_hfp+h_hii+h_pd+sa_bio_02+sa_bio_03+sb_bio_03+sb_bio_04+sb_bio_06+sb_bio_07 + sb_bio_08 + t_flow_acc + t_tpi + t_vrm + w_season, family="binomial", data = Pt_m1_BI_dis_10386_25_pred) |  | 2921.3 | 0.756 | 0.401 | 0.117 | 0.611 | 0.79 | 0.783 |
|  | GLMsel |  | 69 | 25 | 15 |  | glm(P_trigonatus~bio_03+bio_05+bio_13+bio_14+bio_19+dhi_evi_min+dhi_gpp_min+dhi_lai_sea+h_hfp +h_hii+sb_bio_03+sb_bio_06+sb_bio_08+t_tpi+w_season, family="binomial", data = Pt_m1_sel_BI_dis_10386_25_pred) |  | 2908 | 0.767 | 0.412 | 0.12 | 0.622 | 0.79 | 0.784 |
|  | GAM |  | 69 | 25 | 25 |  | gam(P_trigonatus ~ s(bio_03,k=4) + s(bio_05, k=4) + s(bio_13, k=4) + s(bio_14, k=4) + s(bio_19,k=4) + s(dhi_evi_min, k=4) + s(dhi_evi_sea, k=4) + s(dhi_gpp_min, k=4) + s(dhi_gpp_sea, k=4) + s(dhi_lai_min, k=4) + s(dhi_lai_sea, k=4) + s(h_hfp, k=4) + s(h_hii,k=4) + s(h_pd, k=4) + s(sa_bio_02, k=4) +s(sa_bio_03, k=4) + s(sb_bio_03, k=4)+s(sb_bio_04,k=4)+s(sb_bio_06,k=4)+s(sb_bio_07,k=4)+s(sb_bio_08, k=4)+s(t_flow_acc, k=4) + s(t_tpi, k=4)+s(t_vrm, k=4)+s(w_season, k=4), family="binomial", data = Pt_m1_GAM_dis_10386_25_pred, method = "REML") |  | 2770.3 | 0.805 | 0.479 | 0.107 | 0.767 | 0.712 | 0.714 |
|  | GAMsel |  | 69 | 25 | 19 |  | gam(P_trigonatus ~ s(bio_03,k=4) + s(bio_05, k=4) + s(bio_13, k=4) + s(bio_14, k=4) + s(bio_19,k=4) + s(dhi_evi_min, k=4) + s(dhi_gpp_sea, k=4) + s(dhi_lai_min, k=4) + s(h_hfp, k=4) + s(h_hii,k=4) + s(sa_bio_02, k=4) + s(sb_bio_03,k=4)+s(sb_bio_04,k=4)+s(sb_bio_06, k=4) + s(sb_bio_07, k=4) + s(sb_bio_08, k=4) + s(t_flow_acc, k=4) + s(t_tpi, k=4) + s(w_season, k=4), family="binomial", data = Pt_m1_sel_GAM_dis_10386_25_pred, method = "REML", select = TRUE) |  | 2765.4 | 0.808 | 0.49 | 0.143 | 0.697 | 0.794 | 0.79 |
|  |  |  |  |  |  |  |  |  |  |  |  |  |  |  |  |
| **Table S6. Continued** | | | | | | | | | | | | | | | |
| **S13** | GLM |  | 69 (30) | 24 | 24 |  | glm(P_trigonatus ~ bio_05 + bio_13 + bio_14 + bio_19 + dhi_evi_min + dhi_evi_sea + dhi_gpp_min + dhi_gpp_sea + dhi_lai_min + dhi_lai_sea + h_hfp + h_hii + h_pd + sa_bio_02 + sa_bio_03 + sb_bio_03 + sb_bio_04 + sb_bio_06 + sb_bio_07 + sb_bio_08 + t_flow_acc + t_tpi + t_vrm + w_season, family="binomial", data = Pt_m13_BI_dis_10386_24_pred) |  | 2934.6 | 0.744 | 0.371 | 0.108 | 0.583 | 0.788 | 0.78 |
|  | GLMsel |  | 69 (30) | 24 | 15 |  | glm(P_trigonatus ~ bio_13 + bio_14 + bio_19 + dhi_evi_min + dhi_gpp_min + dhi_lai_min + dhi_lai_sea + h_hfp + h_hii + sa_bio_02 + sb_bio_03 + sb_bio_06 + sb_bio_08 + t_tpi + w_season, family="binomial", data = Pt_m13_sel_BI_dis_10386_24_pred) |  | 2918.7 | 0.755 | 0.377 | 0.109 | 0.591 | 0.786 | 0.779 |
|  | GAM |  | 69 (30) | 24 | 24 |  | gam(P_trigonatus ~ s(bio_05,k=4) + s(bio_13, k=4) + s(bio_14, k=4) + s(bio_19, k=4) + s(dhi_evi_min, k=4) + s(dhi_evi_sea,k=4) + s(dhi_gpp_min, k=4) + s(dhi_gpp_sea, k=4) + s(dhi_lai_min, k=4) + s(dhi_lai_sea, k=4) + s(h_hfp,k=4) + s(h_hii,k=4) + s(h_pd, k=4) + s(sa_bio_02, k=4) + s(sa_bio_03, k=4) + s(sb_bio_03, k=4) + s(sb_bio_04, k=4) + s(sb_bio_06, k=4) + s(sb_bio_07, k=4) + s(sb_bio_08, k=4) + s(t_flow_acc, k=4) + s(t_tpi, k=4) + s(t_vrm, k=4) + s(w_season, k=4), family="binomial", data = Pt_m13_GAM_dis_10386_24_pred, method = "REML") |  | 2810.8 | 0.8 | 0.444 | 0.099 | 0.736 | 0.708 | 0.709 |
|  | GAMsel |  | 69 (30) | 24 | 17 |  | gam(P_trigonatus ~ s(bio_13, k=4) + s(bio_14, k=4) + s(bio_19, k=4) + s(dhi_evi_min, k=4) + s(dhi_gpp_sea, k=4) + s(dhi_lai_min, k=4) + s(h_hfp,k=4) + s(h_hii,k=4) + s(sa_bio_02, k=4) + s(sb_bio_03, k=4) + s(sb_bio_04, k=4) + s(sb_bio_06, k=4) + s(sb_bio_07, k=4) + s(sb_bio_08, k=4) + s(t_flow_acc, k=4) + s(t_tpi, k=4) + s(w_season, k=4), family="binomial", data = Pt_m13_sel_GAM_dis_10386_24_pred, method = "REML", select = TRUE) |  | 2801.7 | 0.802 | 0.455 | 0.098 | 0.756 | 0.698 | 0.7 |

**Set:** Set of predictors containing different number of variants.

**Algo.:** Algorithm used to define models’ specification to estimate potential distributions.

**Init.:** Number of initial predictor variants within the predictor set.

**Unco.:** Number of uncorrelated predictor variants selected based on Pearson correlation coefficient (>0.7) and Variation Inflation Factor (VIF).

**Mod.:** Number of predictor variants included in model calibration.

**AIC:** Akaike Information Criteria.

**AUC:** Area under the receiver operator characteristics curve.

**TSS:** True skill statistics.

**Sens.:** Sensitivity. Proportion of true presences classified correctly (as presence).

**Spec.:** Specificity. Proportion of true absences (pseudo-absences) classified correctly (as absence).

**Occ.:** Observation correctly classified. Proportion of both presences and absences (pseudo-absences) classified correctly.

| **Table S7.** Evaluation statistics for abundance models’ structures and specifications defined for *Paleosuchus palpebrosus* | | | | | | | | | | | | | | | | | | |
| --- | --- | --- | --- | --- | --- | --- | --- | --- | --- | --- | --- | --- | --- | --- | --- | --- | --- | --- |
|  |  | **No. of Predictors** | | |  |  | **Observed and predicted abundance agreement** | | | | | | | | | | | |
| **Set** | **Algo.** | **Init.** | **Unco.** | **Mod.** |  | **AIC** | | **E.D.** |  | ***r*** | ***p*** | ***b*** | ***m*** | **RMSE** | **MSE** | **MAE** | **R^2^** | **Adj-R^2^** |
| S5 | GLM Po | 11 | 5 | 5 |  | 2428.3 | | 75.3 |  | 0.93 | 0.32 | -0.53 | 1.18 | 2.67 | 7.13 | 1.57 | 0.86 | 0.86 |
|  | GLM NB | 11 | 5 | 5 |  | 2348.8 | | 68.4 |  | 0.9 | 0.43 | -0.74 | 1.3 | 3.22 | 10.35 | 1.65 | 0.81 | 0.81 |
|  | GLM Ga | 11 | 5 | 5 |  | 2157.8 | | 49.5 |  | 0.85 | 0.23 | -0.87 | 1.22 | 3.67 | 13.43 | 1.84 | 0.72 | 0.72 |
|  | GLMsel Po | 11 | 5 | 5 |  | 2428.3 | | 75.3 |  | 0.93 | 0.32 | -0.53 | 1.18 | 2.67 | 7.13 | 1.57 | 0.86 | 0.86 |
|  | GLMsel NB | 11 | 5 | 4 |  | 2346.9 | | 68.5 |  | 0.9 | 0.43 | -0.75 | 1.3 | 3.18 | 10.08 | 1.64 | 0.82 | 0.82 |
|  | GLMsel Ga | 11 | 5 | 5 |  | 2157.8 | | 49.5 |  | 0.85 | 0.23 | -0.87 | 1.22 | 3.67 | 13.43 | 1.84 | 0.72 | 0.72 |
|  | GAM Po | 11 | 5 | 5 |  | 2215 | | 82.8 |  | 0.94 | 0.52 | -0.48 | 1.17 | 2.43 | 5.91 | 1.23 | 0.89 | 0.89 |
|  | GAM NB | 11 | 5 | 5 |  | 2198.6 | | 80.3 |  | 0.94 | 0.52 | -0.52 | 1.21 | 2.55 | 6.51 | 1.24 | 0.88 | 0.88 |
|  | GAM Ga | 11 | 5 | 5 |  | 1976.4 | | 61.6 |  | 0.93 | 0.46 | -0.75 | 1.2 | 2.7 | 7.29 | 1.34 | 0.86 | 0.86 |
|  | GAMsel Po | 11 | 5 | 4 |  | 2221.5 | | 82.3 |  | 0.94 | 0.51 | -0.57 | 1.21 | 2.51 | 6.31 | 1.27 | 0.89 | 0.89 |
|  | GAMsel NB | 11 | 5 | 4 |  | 2197.6 | | 80 |  | 0.94 | 0.51 | -0.62 | 1.25 | 2.62 | 6.88 | 1.28 | 0.88 | 0.88 |
|  | GAMsel Ga | 11 | 5 | 5 |  | 1983 | | 61 |  | 0.93 | 0.45 | -0.92 | 1.27 | 2.79 | 7.8 | 1.38 | 0.86 | 0.86 |
| S7 | GAM Po | 22 | 5 | 5 |  | 2291 | | 80 |  | 0.92 | 0.45 | -0.62 | 1.05 | 2.69 | 7.26 | 1.67 | 0.84 | 0.84 |
|  | GAM NB | 22 | 5 | 5 |  | 2248.9 | | 76.2 |  | 0.92 | 0.45 | -0.61 | 1.21 | 2.87 | 8.22 | 1.47 | 0.84 | 0.84 |
|  | GAM Ga | 22 | 5 | 5 |  | 2007.8 | | 59.7 |  | 0.86 | 0.43 | -0.76 | 1.25 | 3.58 | 12.84 | 1.59 | 0.74 | 0.74 |
| S8 | GLM Po | 22 | 9 | 9 |  | 2315.8 | | 79.2 |  | 0.94 | 0.42 | -0.38 | 1.13 | 2.41 | 5.79 | 1.41 | 0.88 | 0.88 |
|  | GLM NB | 22 | 9 | 9 |  | 2279.8 | | 74.8 |  | 0.93 | 0.42 | -0.64 | 1.26 | 2.71 | 7.34 | 1.47 | 0.87 | 0.87 |
|  | GLM Ga | 22 | 9 | 9 |  | 2095.9 | | 54.1 |  | 0.88 | 0.3 | -0.88 | 1.22 | 3.4 | 11.58 | 1.75 | 0.77 | 0.77 |
|  | GLMsel Po | 22 | 9 | 8 |  | 2314 | | 79.2 |  | 0.94 | 0.42 | -0.38 | 1.13 | 2.41 | 5.79 | 1.41 | 0.88 | 0.88 |
|  | GLMsel NB | 22 | 9 | 7 |  | 2277.2 | | 74.6 |  | 0.93 | 0.42 | -0.66 | 1.27 | 2.75 | 7.58 | 1.48 | 0.87 | 0.87 |
|  | GLMsel Ga | 22 | 9 | 7 |  | 2092 | | 54.1 |  | 0.88 | 0.29 | -0.88 | 1.22 | 3.4 | 11.54 | 1.75 | 0.77 | 0.77 |
|  | GAM Po | 22 | 9 | 9 |  | 2159.6 | | 85.1 |  | -0.01 | 0.22 | 3.05 | 0 | 27.85 | 775.57 | 11.22 | 0 | 0 |
|  | GAM NB | 22 | 9 | 9 |  | 2150.1 | | 83.5 |  | -0.02 | 0.22 | 3.08 | 0 | 31.38 | 984.43 | 12.45 | 0 | 0 |
|  | GAM Ga | 22 | 9 | 9 |  | 1883.9 | | 67 |  | 0.36 | 0.28 | 1.53 | 0.37 | 7.54 | 56.78 | 3.42 | 0.13 | 0.13 |
|  | GAMsel Po | 22 | 9 | 7 |  | 2151.2 | | 84.9 |  | 0.14 | 0.25 | 2.5 | 0.1 | 11.46 | 131.34 | 5.33 | 0.02 | 0.02 |
|  | GAMsel NB | 22 | 9 | 7 |  | 2146.4 | | 83.3 |  | 0.15 | 0.25 | 2.47 | 0.1 | 11.11 | 123.36 | 5.2 | 0.02 | 0.02 |
|  | GAMsel Ga | 22 | 9 | 7 |  | 1881.3 | | 66.7 |  | 0.11 | 0.24 | 2.71 | 0.06 | 14.2 | 201.67 | 5.15 | 0.01 | 0.01 |
| S9 | GLM Po | 25 | 11 | 11 |  | 2289.1 | | 80.2 |  | 0.94 | 0.43 | -0.34 | 1.11 | 2.33 | 5.42 | 1.35 | 0.89 | 0.89 |
|  | GLM NB | 25 | 11 | 11 |  | 2262.5 | | 76.5 |  | 0.94 | 0.43 | -0.55 | 1.22 | 2.53 | 6.38 | 1.39 | 0.89 | 0.89 |
|  | GLM Ga | 25 | 11 | 11 |  | 2060.6 | | 56.6 |  | 0.91 | 0.34 | -1.11 | 1.32 | 3.19 | 10.17 | 1.67 | 0.82 | 0.82 |
|  | GLMsel Po | 25 | 11 | 9 |  | 2289.1 | | 80.2 |  | 0.94 | 0.42 | 0.02 | 0.89 | 2.41 | 5.79 | 1.38 | 0.89 | 0.89 |
|  | GLMsel NB | 25 | 11 | 9 |  | 2259 | | 76.4 |  | 0.94 | 0.43 | -0.55 | 1.22 | 2.53 | 6.39 | 1.39 | 0.89 | 0.89 |
|  | GLMsel Ga | 25 | 11 | 9 |  | 2057.3 | | 56.5 |  | 0.9 | 0.34 | -1.11 | 1.32 | 3.23 | 10.42 | 1.68 | 0.81 | 0.81 |
|  | GAM Po | 25 | 11 | 11 |  | 2150.8 | | 85.4 |  | -0.02 | 0.27 | 3.04 | -5E-10 | 3.54E+8 | 1.25E+17 | 5.22E+7 | 0 | 0 |
|  | GAM NB | 25 | 11 | 11 |  | 2142.4 | | 84 |  | -0.02 | 0.27 | 3.04 | -8E-10 | 2.06E+8 | 4.26E+16 | 3.07E+7 | 0 | 0 |
|  | GAM Ga | 25 | 11 | 11 |  | 1848.5 | | 69 |  | -0.02 | 0.26 | 3.04 | -7E-10 | 2.36E+8 | 5.61E+16 | 3.46E+7 | 0 | 0 |
|  | GAMsel Po | 25 | 11 | 8 |  | 2148.6 | | 85 |  | 0.02 | 0.23 | 2.95 | 0.01 | 21.16 | 447.59 | 8.99 | 0 | 0 |
|  | GAMsel NB | 25 | 11 | 7 |  | 2152.3 | | 82.7 |  | -0.02 | 0.22 | 3.08 | -0.01 | 28.78 | 828.57 | 11.5 | 0 | 0 |
|  | GAMsel Ga | 25 | 11 | 9 |  | 1852.7 | | 68.2 |  | 0.02 | 0.22 | 2.97 | 0.01 | 25.45 | 647.54 | 8.85 | 0 | 0 |
| S10 | GLM Po | 32 | 15 | 15 |  | 2230.6 | | 82.4 |  | 0.94 | 0.5 | -0.18 | 1.06 | 2.36 | 5.56 | 1.23 | 0.88 | 0.88 |
|  | GLM NB | 32 | 15 | 15 |  | 2209 | | 80 |  | 0.93 | 0.5 | -0.18 | 1.08 | 2.49 | 6.19 | 1.23 | 0.87 | 0.87 |
|  | GLM Ga | 32 | 15 | 15 |  | 1979.8 | | 61.7 |  | 0.92 | 0.43 | -0.55 | 1.13 | 2.78 | 7.72 | 1.39 | 0.84 | 0.84 |
|  | GLMsel Po | 32 | 15 | 9 |  | 2220.9 | | 82.3 |  | 0.94 | 0.49 | -0.19 | 1.06 | 2.34 | 5.48 | 1.23 | 0.88 | 0.88 |
|  | GLMsel NB | 32 | 15 | 9 |  | 2199.7 | | 79.9 |  | 0.93 | 0.5 | -0.2 | 1.08 | 2.47 | 6.08 | 1.24 | 0.87 | 0.87 |
|  | GLMsel Ga | 32 | 15 | 11 |  | 1974.5 | | 61.5 |  | 0.92 | 0.43 | -0.57 | 1.14 | 2.78 | 7.71 | 1.4 | 0.84 | 0.84 |
|  | GAM Po | 32 | 15 | 15 |  | 2157.5 | | 85.4 |  | 0.93 | 0.56 | 0.59 | 0.65 | 4.17 | 17.35 | 1.53 | 0.87 | 0.87 |
|  | GAM NB | 32 | 15 | 15 |  | 2149.7 | | 84 |  | 0.92 | 0.56 | 0.87 | 0.53 | 6.16 | 37.9 | 1.83 | 0.84 | 0.84 |
|  | GAM Ga | 32 | 15 | 15 |  | 1854.8 | | 69.1 |  | 0.77 | 0.46 | 2.03 | 0.05 | 92.19 | 8498.19 | 16.07 | 0.59 | 0.59 |
|  | GAMsel Po | 32 | 15 | 7 |  | 2150.2 | | 84.9 |  | 0.94 | 0.53 | -0.7 | 1.3 | 2.65 | 7.04 | 1.29 | 0.89 | 0.89 |
|  | GAMsel NB | 32 | 15 | 7 |  | 2142.1 | | 83.3 |  | 0.94 | 0.55 | -0.81 | 1.37 | 2.8 | 7.82 | 1.28 | 0.89 | 0.89 |
|  | GAMsel Ga | 32 | 15 | 10 |  | 1844.1 | | 68.7 |  | 0.16 | 0.29 | 2.81 | 0.02 | 55.56 | 3087.17 | 9.6 | 0.02 | 0.02 |
| S11 | GLM Po | 47 | 19 | 19 |  | 2210.9 | | 83.3 |  | 0.94 | 0.49 | -0.15 | 1.05 | 2.26 | 5.1 | 1.2 | 0.89 | 0.89 |
|  | GLM NB | 47 | 19 | 19 |  | 2197.2 | | 81.4 |  | 0.94 | 0.5 | -0.14 | 1.06 | 2.38 | 5.66 | 1.2 | 0.88 | 0.88 |
|  | GLM Ga | 47 | 19 | 19 |  | 1975 | | 62.4 |  | 0.92 | 0.44 | -0.57 | 1.14 | 2.75 | 7.58 | 1.37 | 0.84 | 0.84 |
|  | GLMsel Po | 47 | 19 | 9 |  | 2196.7 | | 83.1 |  | 0.94 | 0.48 | -0.15 | 1.05 | 2.26 | 5.11 | 1.21 | 0.89 | 0.89 |
|  | GLMsel NB | 47 | 19 | 9 |  | 2182.6 | | 81.2 |  | 0.94 | 0.48 | -0.14 | 1.06 | 2.36 | 5.55 | 1.21 | 0.88 | 0.88 |
|  | GLMsel Ga | 47 | 19 | 14 |  | 1968.7 | | 62.2 |  | 0.92 | 0.43 | -0.62 | 1.16 | 2.75 | 7.59 | 1.38 | 0.85 | 0.85 |
|  |  |  |  |  |  |  | |  |  |  |  |  |  |  |  |  |  |  |
|  |  |  |  |  |  |  | |  |  |  |  |  |  |  |  |  |  |  |
| **Table S7. Continued.** | | | | | | | | | | | | | | | | | | |
| S11 | GAM Po | 47 | 19 | 19 |  | 2147.9 | | 86.3 |  | 0.93 | 0.55 | 0.71 | 0.61 | 4.65 | 21.58 | 1.6 | 0.87 | 0.87 |
|  | GAM NB | 47 | 19 | 19 |  | 2144.6 | | 85.1 |  | 0.93 | 0.53 | 0.74 | 0.61 | 4.77 | 22.75 | 1.63 | 0.86 | 0.86 |
|  | GAM Ga | 47 | 19 | 19 |  | 1837 | | 70.5 |  | 0.85 | 0.52 | 1.55 | 0.15 | 33.18 | 1100.89 | 7.35 | 0.73 | 0.73 |
|  | GAMsel Po | 47 | 19 | 10 |  | 2129.1 | | 86 |  | 0.86 | 0.42 | -1.14 | 1.42 | 3.84 | 14.78 | 1.86 | 0.73 | 0.73 |
|  | GAMsel NB | 47 | 19 | 7 |  | 2137.6 | | 83.6 |  | 0.93 | 0.57 | -0.09 | 1 | 2.38 | 5.68 | 1.17 | 0.87 | 0.87 |
|  | GAMsel Ga | 47 | 19 | 12 |  | 1823.9 | | 69.9 |  | 0.39 | 0.31 | 2.06 | 0.19 | 12.98 | 168.6 | 3.59 | 0.15 | 0.15 |
| S12 | GLM Po | 69 | 20 | 20 |  | 2352.3 | | 78.7 |  | 0.95 | 0.4 | -0.29 | 1.1 | 2.21 | 4.89 | 1.33 | 0.9 | 0.9 |
|  | GLM NB | 69 | 20 | 20 |  | 2331.6 | | 73.5 |  | 0.94 | 0.4 | -0.57 | 1.24 | 2.5 | 6.25 | 1.39 | 0.89 | 0.89 |
|  | GLM Ga | 69 | 20 | 20 |  | 2112 | | 54.5 |  | 0.87 | 0.35 | -1.28 | 1.4 | 3.65 | 13.35 | 1.76 | 0.76 | 0.76 |
|  | GLMsel Po | 69 | 20 | 14 |  | 2342.7 | | 78.7 |  | 0.95 | 0.4 | -0.31 | 1.11 | 2.2 | 4.83 | 1.33 | 0.9 | 0.9 |
|  | GLMsel NB | 69 | 20 | 12 |  | 2320.5 | | 72.9 |  | 0.94 | 0.39 | -0.67 | 1.27 | 2.6 | 6.75 | 1.43 | 0.89 | 0.89 |
|  | GLMsel Ga | 69 | 20 | 11 |  | 2098.8 | | 54.2 |  | 0.89 | 0.34 | -1.33 | 1.42 | 3.52 | 12.38 | 1.73 | 0.79 | 0.79 |
|  | GAM Po | 69 | 20 | 20 |  | 2144.4 | | 86.7 |  | 0.25 | 0.33 | 1.24 | 0.64 | 6.54 | 42.81 | 2.84 | 0.06 | 0.06 |
|  | GAM NB | 69 | 20 | 20 |  | 2147.7 | | 85.8 |  | 0.13 | 0.26 | 2.27 | 0.24 | 7.24 | 52.36 | 3.32 | 0.02 | 0.02 |
|  | GAM Ga | 69 | 20 | 20 |  | 1882.1 | | 68.9 |  | 0.74 | 0.55 | -2.61 | 2.03 | 5.14 | 26.45 | 2.03 | 0.55 | 0.55 |
|  | GAMsel Po | 69 | 20 | 10 |  | 2125.8 | | 86.3 |  | -0.07 | 0.17 | 3.22 | -0.02 | 34.48 | 1188.57 | 13.73 | 0.01 | 0 |
|  | GAMsel NB | 69 | 20 | 9 |  | 2135.1 | | 84.8 |  | -0.07 | 0.19 | 3.21 | -0.02 | 27.44 | 753.06 | 11.52 | 0 | 0 |
|  | GAMsel Ga | 69 | 20 | 12 |  | 1877.2 | | 67.6 |  | 0.94 | 0.52 | -1.45 | 1.52 | 3.14 | 9.86 | 1.44 | 0.88 | 0.88 |
| S13 | GLM Po | 25 | 22 | 22 |  | 2235.4 | | 82.7 |  | 0.94 | 0.53 | -0.27 | 1.1 | 2.34 | 5.47 | 1.2 | 0.88 | 0.88 |
|  | GLM NB | 25 | 22 | 22 |  | 2214.1 | | 80.4 |  | 0.94 | 0.53 | -0.35 | 1.15 | 2.47 | 6.12 | 1.23 | 0.88 | 0.88 |
|  | GLM Ga | 25 | 22 | 22 |  | 1961.6 | | 63.4 |  | 0.92 | 0.49 | -0.92 | 1.28 | 2.93 | 8.61 | 1.44 | 0.85 | 0.85 |
|  | GLMsel Po | 25 | 22 | 15 |  | 2225.5 | | 82.6 |  | 0.94 | 0.53 | -0.27 | 1.1 | 2.34 | 5.47 | 1.21 | 0.88 | 0.88 |
|  | GLMsel NB | 25 | 22 | 12 |  | 2201.1 | | 80 |  | 0.94 | 0.54 | -0.4 | 1.16 | 2.46 | 6.07 | 1.24 | 0.88 | 0.88 |
|  | GLMsel Ga | 25 | 22 | 13 |  | 1950.5 | | 63 |  | 0.92 | 0.49 | -0.92 | 1.27 | 2.94 | 8.63 | 1.46 | 0.85 | 0.84 |
|  | GAM Po | 25 | 22 | 22 |  | 2145.9 | | 86.5 |  | 0.75 | 0.36 | 0.77 | 0.58 | 5.78 | 33.4 | 2.28 | 0.56 | 0.56 |
|  | GAM NB | 25 | 22 | 22 |  | 2143 | | 85.3 |  | 0.9 | 0.49 | 0.7 | 0.63 | 4.67 | 21.78 | 1.78 | 0.81 | 0.81 |
|  | GAM Ga | 25 | 22 | 22 |  | 1837.2 | | 70.8 |  | 0.53 | 0.39 | 1.74 | 0.28 | 10.8 | 116.63 | 2.85 | 0.28 | 0.28 |
|  | GAMsel Po | 25 | 22 | 10 |  | 2124.3 | | 86.1 |  | -0.03 | 0.23 | 3.07 | 0 | 57.41 | 3296.38 | 15.98 | 0 | 0 |
|  | GAMsel NB | 25 | 22 | 9 |  | 2121 | | 85 |  | 0 | 0.24 | 3.03 | 0 | 28.59 | 817.52 | 10.1 | 0 | 0 |
|  | GAMsel Ga | 25 | 22 | 11 |  | 1816 | | 70.1 |  | -0.03 | 0.22 | 3.06 | 0 | 101.28 | 10257.86 | 20.65 | 0 | 0 |

**Algo.:** Algorithm used to define the models’ specifications and estimate relative abundances.

**Init.:** Number of initial predictor variants within the predictor set.

**Unco.:** Number of uncorrelated predictor variants selected based on Pearson correlation coefficient (>0.7) and Variation Inflation Factor (VIF).

**Mod.:** Number of predictor variants included in model calibration.

**AIC:** Akaike Information Criteria.

**E.D.:** Explained Deviance. Indicates the amount of variation in relative abundance explained by predictors.

***r*:** Pearson’s correlation coefficient. Indicates the agreement between observed and predicted values.

***ρ*:** Rho Spearman’s rank correlation coefficient.

***b:*** Intercept of the fitted line between “observed” (y-axis) and “predicted” (x-axis) relative abundances, following the recommendation of Piñero et al., (2008).

***m:*** Slope of the fitted line between “observed” and “predicted” (x-axis) relative abundances.

**RMSE:** Root Mean Squared Error.

**MSE:** Mean Squared Error.

**MAE:** Mean Absolute Error.

**R^2^:** R Squared.

**Adj-R^2^:** Adjusted R Squared.

| **Table S8.** Evaluation statistics for abundance models’ structures and specifications defined for *Paleosuchus trigonatus.* | | | | | | | | | | | | | | | | | | |
| --- | --- | --- | --- | --- | --- | --- | --- | --- | --- | --- | --- | --- | --- | --- | --- | --- | --- | --- |
|  |  | **No. of Predictors** | | |  |  | **Observed and predicted abundance agreement** | | | | | | | | | | | |
| **Set** | **Algo.** | **Init.** | **Unco.** | **Mod.** |  | **AIC** | | **E.D.** |  | ***r*** | ***ρ*** | ***b*** | ***m*** | **RMSE** | **MSE** | **MAE** | **R^2^** | **Adj-R^2^** |
| S2 | GLM Po | 3 | 3 | 3 |  | 4226 | | 62.7 |  | 0.74 | 0.44 | 0.12 | 0.96 | 2.69 | 7.23 | 1.81 | 0.54 | 0.54 |
|  | GLM NB | 3 | 3 | 3 |  | 3979 | | 54.4 |  | 0.71 | 0.42 | -0.12 | 1 | 2.82 | 7.92 | 1.91 | 0.5 | 0.5 |
|  | GLM Ga | 3 | 3 | 3 |  | 4073.6 | | 30.2 |  | 0.53 | 0.35 | -0.23 | 0.85 | 3.5 | 12.22 | 2.57 | 0.28 | 0.28 |
|  | GLMsel Po | 3 | 3 | 3 |  | 4226 | | 62.7 |  | 0.74 | 0.44 | 0.12 | 0.96 | 2.69 | 7.23 | 1.81 | 0.54 | 0.54 |
|  | GLMsel NB | 3 | 3 | 3 |  | 3979 | | 54.4 |  | 0.71 | 0.42 | -0.12 | 1 | 2.82 | 7.93 | 1.91 | 0.5 | 0.5 |
|  | GLMsel Ga | 3 | 3 | 3 |  | 4073.6 | | 30.2 |  | 0.53 | 0.35 | -0.23 | 0.85 | 3.5 | 12.22 | 2.57 | 0.28 | 0.28 |
|  | GAM Po | 3 | 3 | 3 |  | 4003.9 | | 67.8 |  | 0.8 | 0.44 | 0.27 | 0.89 | 2.42 | 5.84 | 1.64 | 0.64 | 0.64 |
|  | GAM NB | 3 | 3 | 3 |  | 3874 | | 61.1 |  | 0.79 | 0.43 | 0.08 | 0.92 | 2.48 | 6.13 | 1.71 | 0.62 | 0.62 |
|  | GAM Ga | 3 | 3 | 3 |  | 3954.5 | | 37.6 |  | 0.69 | 0.36 | -0.33 | 0.87 | 3.02 | 9.1 | 2.21 | 0.48 | 0.48 |
|  | GAMsel Po | 3 | 3 | 3 |  | 4003.2 | | 67.8 |  | 0.8 | 0.44 | 0.26 | 0.89 | 2.42 | 5.84 | 1.64 | 0.64 | 0.64 |
|  | GAMsel NB | 3 | 3 | 3 |  | 3873 | | 61.1 |  | 0.79 | 0.43 | 0.07 | 0.92 | 2.48 | 6.13 | 1.71 | 0.62 | 0.62 |
|  | GAMsel Ga | 3 | 3 | 3 |  | 3953 | | 37.6 |  | 0.69 | 0.36 | -0.34 | 0.87 | 3.03 | 9.15 | 2.21 | 0.48 | 0.48 |
| S9 | GLM Po | 25 | 12 | 12 |  | 3897.1 | | 70.3 |  | 0.8 | 0.44 | 0.15 | 0.95 | 2.41 | 5.79 | 1.6 | 0.64 | 0.63 |
|  | GLM NB | 25 | 12 | 12 |  | 3776 | | 66 |  | 0.78 | 0.43 | 0.04 | 0.97 | 2.47 | 6.11 | 1.67 | 0.61 | 0.61 |
|  | GLM Ga | 25 | 12 | 12 |  | 3676.5 | | 52.9 |  | 0.74 | 0.38 | -0.3 | 0.93 | 2.76 | 7.61 | 1.98 | 0.54 | 0.54 |
|  | GLMsel Po | 25 | 12 | 9 |  | 3892.2 | | 70.3 |  | 0.8 | 0.44 | 0.16 | 0.95 | 2.41 | 5.83 | 1.61 | 0.63 | 0.63 |
|  | GLMsel NB | 25 | 12 | 8 |  | 3770.7 | | 65.9 |  | 0.78 | 0.43 | 0.05 | 0.96 | 2.48 | 6.17 | 1.68 | 0.61 | 0.61 |
|  | GLMsel Ga | 25 | 12 | 9 |  | 3667.1 | | 53.1 |  | 0.73 | 0.38 | -0.28 | 0.92 | 2.79 | 7.76 | 2 | 0.53 | 0.53 |
|  | GAM Po | 25 | 12 | 12 |  | 3647 | | 76.3 |  | -0.22 | -0.23 | 4.32 | -0.38 | 5.05 | 25.53 | 3.44 | 0.05 | 0.05 |
|  | GAM NB | 25 | 12 | 12 |  | 3628.1 | | 74.1 |  | -0.19 | -0.19 | 4.18 | -0.34 | 4.86 | 23.61 | 3.25 | 0.03 | 0.03 |
|  | GAM Ga | 25 | 12 | 12 |  | 3580.7 | | 58 |  | 0.03 | -0.02 | 2.99 | 0.05 | 4.46 | 19.93 | 3.05 | 0 | 0 |
|  | GAMsel Po | 25 | 12 | 7 |  | 3749.8 | | 73.5 |  | 0.79 | 0.31 | 1.9 | 0.02 | 141.06 | 19898 | 52.71 | 0.63 | 0.63 |
|  | GAMsel NB | 25 | 12 | 6 |  | 3685.8 | | 70.4 |  | 0.77 | 0.31 | 1.94 | 0.05 | 56.94 | 3241.68 | 20.92 | 0.59 | 0.59 |
|  | GAMsel Ga | 25 | 12 | 5 |  | 3617.3 | | 55.2 |  | 0.72 | 0.39 | 1.8 | 0.17 | 14.75 | 217.65 | 5.55 | 0.52 | 0.52 |
| S10 | GLM Po | 32 | 15 | 15 |  | 3895.2 | | 70.5 |  | 0.8 | 0.44 | 0.17 | 0.95 | 2.4 | 5.76 | 1.6 | 0.64 | 0.64 |
|  | GLM NB | 32 | 15 | 15 |  | 3777.6 | | 66.2 |  | 0.79 | 0.43 | 0.06 | 0.96 | 2.46 | 6.07 | 1.67 | 0.62 | 0.62 |
|  | GLM Ga | 32 | 15 | 15 |  | 3676.7 | | 53.2 |  | 0.74 | 0.37 | -0.26 | 0.92 | 2.75 | 7.57 | 1.98 | 0.55 | 0.55 |
|  | GLMsel Po | 32 | 15 | 9 |  | 3886.3 | | 70.4 |  | 0.8 | 0.44 | 0.18 | 0.95 | 2.41 | 5.79 | 1.61 | 0.64 | 0.64 |
|  | GLMsel NB | 32 | 15 | 9 |  | 3768.7 | | 66.1 |  | 0.79 | 0.43 | 0.08 | 0.95 | 2.47 | 6.1 | 1.67 | 0.62 | 0.62 |
|  | GLMsel Ga | 32 | 15 | 10 |  | 3665.1 | | 53.3 |  | 0.73 | 0.37 | -0.24 | 0.91 | 2.78 | 7.74 | 2 | 0.54 | 0.54 |
|  | GAM Po | 32 | 15 | 15 |  | 3647.5 | | 76.5 |  | -0.28 | -0.28 | 4.56 | -0.46 | 5.22 | 27.24 | 3.51 | 0.08 | 0.08 |
|  | GAM NB | 32 | 15 | 15 |  | 3653.7 | | 72.8 |  | -0.18 | -0.16 | 4.16 | -0.36 | 4.75 | 22.59 | 3.09 | 0.03 | 0.03 |
|  | GAM Ga | 32 | 15 | 15 |  | 3586.3 | | 57.4 |  | 0.33 | 0.16 | 1.02 | 0.66 | 3.81 | 14.53 | 2.56 | 0.11 | 0.11 |
|  | GAMsel Po | 32 | 15 | 9 |  | 3731.8 | | 73.9 |  | 0.85 | 0.49 | 1.77 | 0.01 | 16.08 | 258.59 | 6.65 | 0.69 | 0.69 |
|  | GAMsel NB | 32 | 15 | 8 |  | 3644.2 | | 72.6 |  | 0.58 | 0.28 | -0.31 | 1.33 | 3.34 | 11.16 | 2.11 | 0.33 | 0.33 |
|  | GAMsel Ga | 32 | 15 | 6 |  | 3602.6 | | 56 |  | 0.63 | 0.25 | 0.42 | 0.75 | 3.22 | 10.4 | 2.3 | 0.4 | 0.4 |
| S11 | GLM Po | 47 | 20 | 20 |  | 3836.8 | | 72 |  | 0.82 | 0.45 | 0.18 | 0.95 | 2.29 | 5.22 | 1.53 | 0.67 | 0.67 |
|  | GLM NB | 47 | 20 | 20 |  | 3758.6 | | 68 |  | 0.81 | 0.44 | 0.06 | 0.97 | 2.35 | 5.51 | 1.59 | 0.65 | 0.65 |
|  | GLM Ga | 47 | 20 | 20 |  | 3677.4 | | 53.6 |  | 0.75 | 0.37 | -0.33 | 0.94 | 2.67 | 7.15 | 1.93 | 0.57 | 0.57 |
|  | GLMsel Po | 47 | 20 | 12 |  | 3825.8 | | 71.9 |  | 0.82 | 0.45 | 0.19 | 0.94 | 2.29 | 5.24 | 1.53 | 0.67 | 0.67 |
|  | GLMsel NB | 47 | 20 | 12 |  | 3826.8 | | 67.8 |  | 0.81 | 0.44 | 0.08 | 0.96 | 2.36 | 5.55 | 1.59 | 0.65 | 0.65 |
|  | GAM Po | 47 | 20 | 20 |  | 3674 | | 76.3 |  | -0.3 | -0.31 | 4.4 | -0.29 | 6.7 | 44.85 | 4.68 | 0.09 | 0.09 |
|  | GAM NB | 47 | 20 | 20 |  | 3657.8 | | 73.2 |  | -0.21 | -0.19 | 4.17 | -0.3 | 5.36 | 28.7 | 3.58 | 0.05 | 0.04 |
|  | GAM Ga | 47 | 20 | 20 |  | 3593.2 | | 57.7 |  | -0.11 | -0.1 | 3.77 | -0.16 | 5.04 | 25.4 | 3.38 | 0.01 | 0.01 |
|  | GAMsel Po | 47 | 20 | 10 |  | 3712.4 | | 74.6 |  | 0.38 | 0.2 | 1.57 | 0.55 | 3.89 | 15.11 | 2.36 | 0.15 | 0.15 |
|  | GAMsel NB | 47 | 20 | 8 |  | 3677.5 | | 71.1 |  | 0.7 | 0.49 | 2.21 | 0.03 | 107.61 | 11580.6 | 34.28 | 0.49 | 0.49 |
|  | GAMsel Ga | 47 | 20 | 7 |  | 3602.6 | | 56 |  | 0.01 | 0.01 | 3.08 | 0.03 | 4.48 | 20.06 | 2.87 | 0 | 0 |
| S12 | GLM Po | 69 | 25 | 25 |  | 3828.1 | | 72.4 |  | 0.81 | 0.46 | 0.35 | 0.89 | 2.34 | 5.47 | 1.56 | 0.66 | 0.66 |
|  | GLM NB | 69 | 25 | 25 |  | 3744 | | 68.9 |  | 0.8 | 0.46 | 0.4 | 0.85 | 2.47 | 6.1 | 1.62 | 0.63 | 0.63 |
|  | GLM Ga | 69 | 25 | 25 |  | 3657.1 | | 55 |  | 0.75 | 0.4 | 0.03 | 0.83 | 2.76 | 7.6 | 1.94 | 0.57 | 0.57 |
|  | GLMsel Po | 69 | 25 | 13 |  | 3810.7 | | 72.2 |  | 0.81 | 0.45 | 0.35 | 0.89 | 2.36 | 5.57 | 1.57 | 0.66 | 0.66 |
|  | GLMsel NB | 69 | 25 | 12 |  | 3723.5 | | 68.7 |  | 0.79 | 0.45 | 0.39 | 0.85 | 2.49 | 6.22 | 1.65 | 0.63 | 0.63 |
|  | GLMsel Ga | 69 | 25 | 13 |  | 3639.2 | | 54.7 |  | 0.74 | 0.4 | 0.06 | 0.82 | 2.81 | 7.91 | 1.98 | 0.55 | 0.55 |
|  | GAM Po | 69 | 25 | 25 |  | 3601.8 | | 78.2 |  | 0.69 | 0.52 | 2.42 | 0 | 615047.9 | 3.78E+11 | 163511.2 | 0.47 | 0.47 |
|  | GAM NB | 69 | 25 | 25 |  | 3600.7 | | 76.7 |  | 0.69 | 0.52 | 2.42 | 0 | 362627 | 1.31E+11 | 96401.84 | 0.47 | 0.47 |
|  | GAM Ga | 69 | 25 | 25 |  | 3576.2 | | 59.3 |  | 0.68 | 0.5 | 2.39 | 0 | 1755.58 | 3.08E+06 | 482.34 | 0.47 | 0.47 |
|  | GAMsel Po | 69 | 25 | 13 |  | 3603.5 | | 77.3 |  | 0.69 | 0.52 | 2.4 | 0 | 298239.8 | 8.89E+10 | 81488.6 | 0.47 | 0.47 |
|  | GAMsel NB | 69 | 25 | 12 |  | 3593.1 | | 75.5 |  | 0.68 | 0.51 | 2.37 | 0 | 5136.1 | 2.63E+07 | 1454.01 | 0.47 | 0.46 |
|  | GAMsel Ga | 69 | 25 | 9 |  | 3569.4 | | 57.8 |  | 0.7 | 0.46 | 2.26 | 0.03 | 92.53 | 8561.2 | 28.01 | 0.48 | 0.48 |
|  |  |  |  |  |  |  | |  |  |  |  |  |  |  |  |  |  |  |
|  |  |  |  |  |  |  | |  |  |  |  |  |  |  |  |  |  |  |
| **Table S8. Continued.** | | | | | | | | | | | | | | | | | | |
| S13 | GLM Po | 30 | 24 | 24 |  | 3828.7 | | 72.3 |  | 0.81 | 0.46 | 0.34 | 0.9 | 2.34 | 5.46 | 1.56 | 0.66 | 0.66 |
|  | GLM NB | 30 | 24 | 24 |  | 3744.5 | | 68.8 |  | 0.8 | 0.46 | 0.39 | 0.85 | 2.47 | 6.09 | 1.63 | 0.63 | 0.63 |
|  | GLM Ga | 30 | 24 | 24 |  | 3655.1 | | 55 |  | 0.75 | 0.4 | 0.03 | 0.83 | 2.76 | 7.6 | 1.94 | 0.57 | 0.57 |
|  | GLMsel Po | 30 | 24 | 13 |  | 3810.9 | | 72.2 |  | 0.81 | 0.46 | 0.33 | 0.9 | 2.35 | 5.51 | 1.58 | 0.66 | 0.66 |
|  | GLMsel NB | 30 | 24 | 12 |  | 3723.5 | | 68.7 |  | 0.79 | 0.45 | 0.39 | 0.85 | 2.49 | 6.22 | 1.65 | 0.63 | 0.63 |
|  | GLMsel Ga | 30 | 24 | 13 |  | 3639.2 | | 54.7 |  | 0.74 | 0.4 | 0.06 | 0.82 | 2.81 | 7.91 | 1.98 | 0.55 | 0.55 |
|  | GAM Po | 30 | 24 | 24 |  | 3603.3 | | 78.1 |  | 0.69 | 0.51 | 2.4 | 0 | 24558.29 | 6.03E+08 | 6622.53 | 0.47 | 0.47 |
|  | GAM NB | 30 | 24 | 24 |  | 3601.6 | | 76.6 |  | 0.69 | 0.51 | 2.4 | 0 | 13269.02 | 1.76E+08 | 3593.08 | 0.48 | 0.48 |
|  | GAM Ga | 30 | 24 | 24 |  | 3577.2 | | 59.1 |  | 0.73 | 0.47 | 2.23 | 0.03 | 113.33 | 12842.73 | 33.9 | 0.53 | 0.53 |
|  | GAMsel Po | 30 | 24 | 12 |  | 3601.5 | | 77.2 |  | 0.69 | 0.51 | 2.34 | 0 | 2552.74 | 6.51E+06 | 737.53 | 0.48 | 0.48 |
|  | GAMsel NB | 30 | 24 | 10 |  | 3627.8 | | 73.6 |  | 0.65 | 0.43 | 1.95 | 0.27 | 2.31 | 5.31 | 1.51 | 7.10E-01 | 0.71 |
|  | GAMsel Ga | 30 | 24 | 8 |  | 3576.7 | | 57.4 |  | 0.73 | 0.36 | -0.61 | 1.09 | 2.74 | 7.5 | 1.96 | 0.53 | 0.53 |

**Algo.:** Algorithm used to define the models’ specifications and estimate relative abundances.

**Init.:** Number of initial predictor variants within the predictor set.

**Unco.:** Number of uncorrelated predictor variants selected based on Pearson correlation coefficient (>0.7) and Variation Inflation Factor (VIF).

**Mod.:** Number of predictor variants included in model calibration.

**AIC:** Akaike Information Criteria.

**E.D.:** Explained Deviance. Indicates the amount of variation in relative abundance explained by predictors.

***r*:** Pearson’s correlation coefficient. Indicates the agreement between observed and predicted values.

***ρ*:** Rho Spearman’s rank correlation coefficient.

***b:*** Intercept of the fitted line between “observed” (y-axis) and “predicted” (x-axis) relative abundances, following the recommendation of Piñero et al., (2008).

***m:*** Slope of the fitted line between “observed” and “predicted” (x-axis) relative abundances.

**RMSE:** Root Mean Squared Error.

**MSE:** Mean Squared Error.

**MAE:** Mean Absolute Error.

**R^2^:** R Squared.

**Adj-R^2^:** Adjusted R Squared.

* **Note:** Model “S11 - GLMsel Gamma” was not able to be run on Rstudio. Thus, no evaluation statistics are shown for this model.


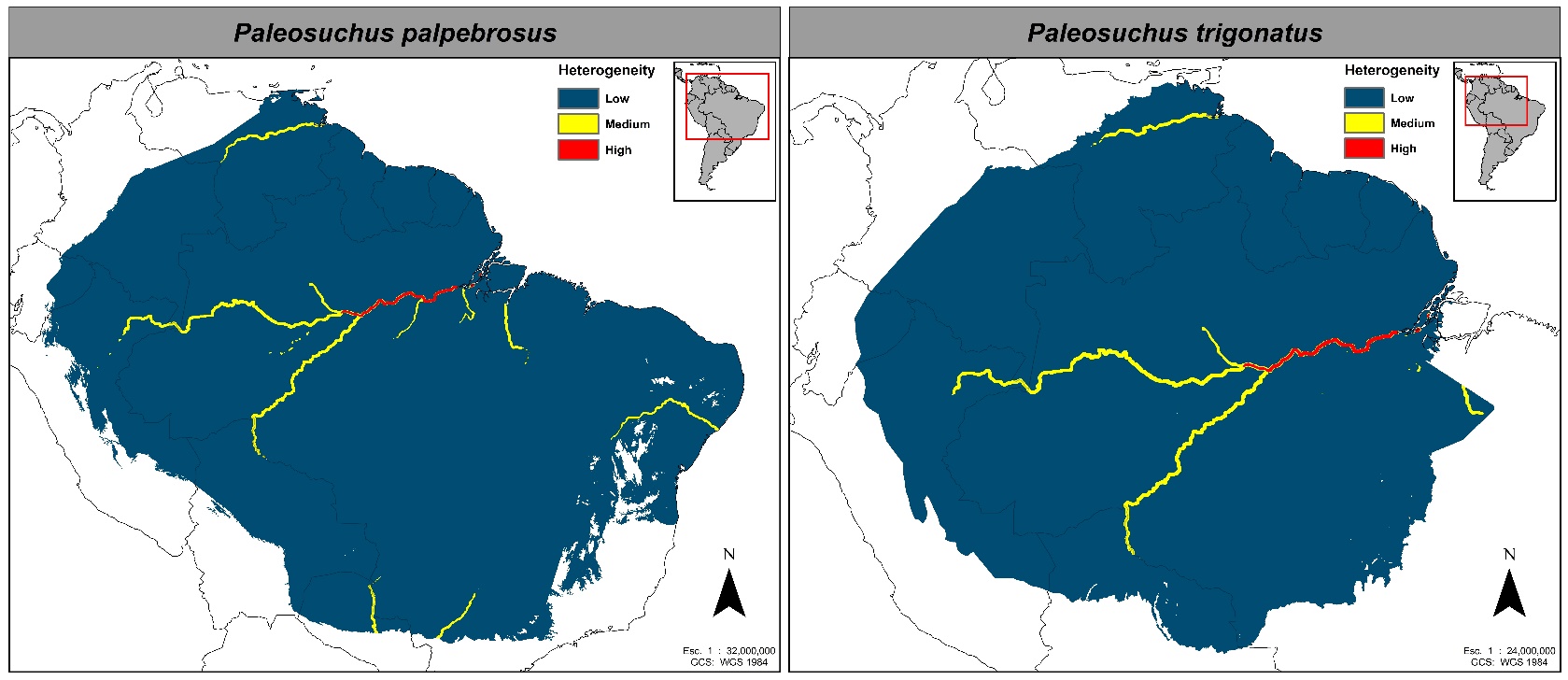


**Figure S1.** Environmental heterogeneity within the study area extent for *Paleosuchus palpebrosus* and *Paleosuchus trigonatus.*


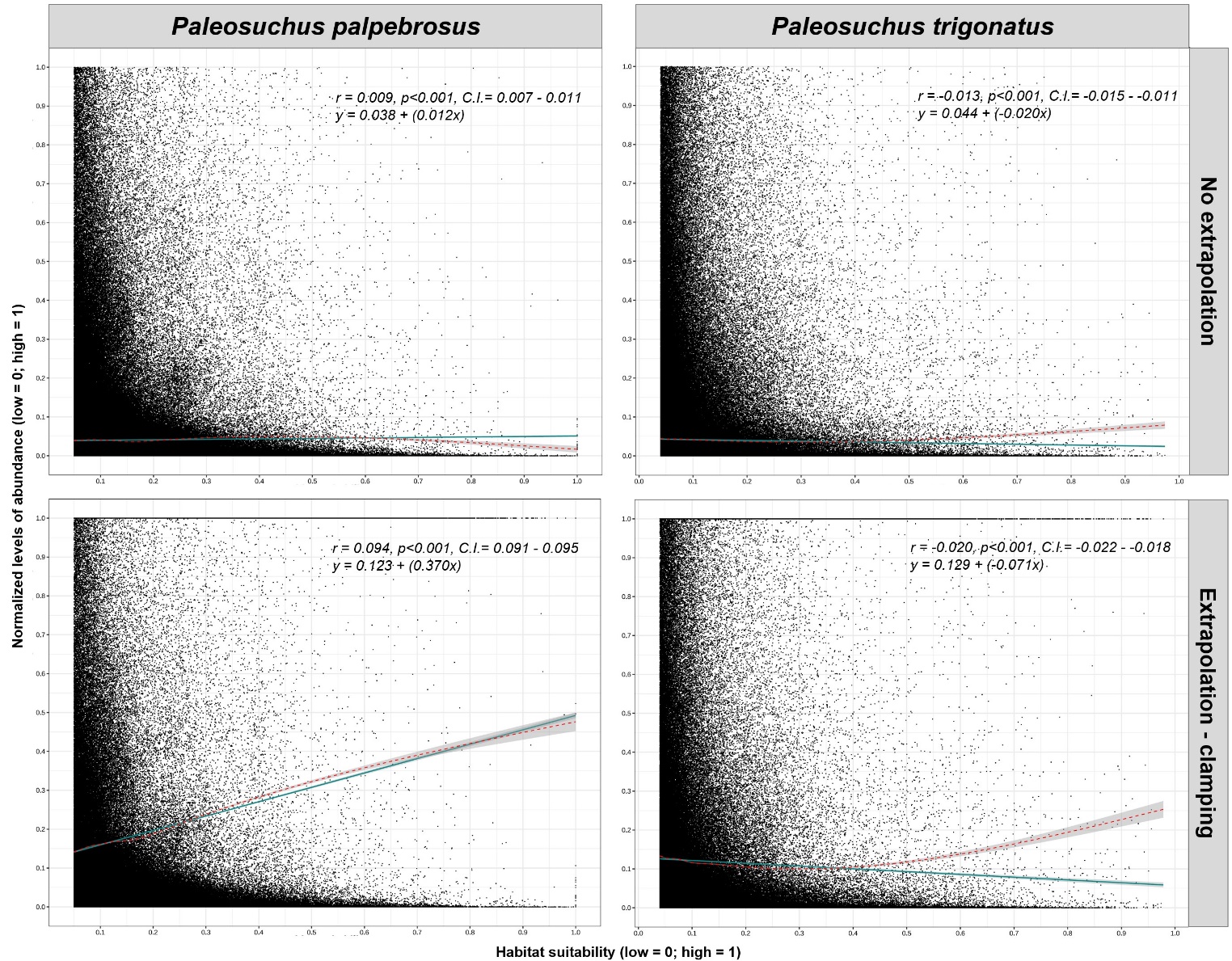


**Figure S2.** Analysis of the relationship between suitability and abundance derived from the best models for *Paleosuchus palpebrosus* (GAM-S13 and Poisson GLM_sel_-S12, respectively) and *P. trigonatus* (GAM_sel_-S12 and Poisson GAM_sel_-S10, respectively). Top facets correspond to abundance projections via the “no extrapolation method, whereas bottom facets correspond to projections via the “extrapolation and clamping” method. The linear regression model is depicted with a green continuous line, whereas a smooth curve using the “loess” method to fit the dataset is depicted with a dashed red line. Pearson’s correlation coefficient, the formula of the linear regression model, and confidence intervals (95%) are shown in each inset.


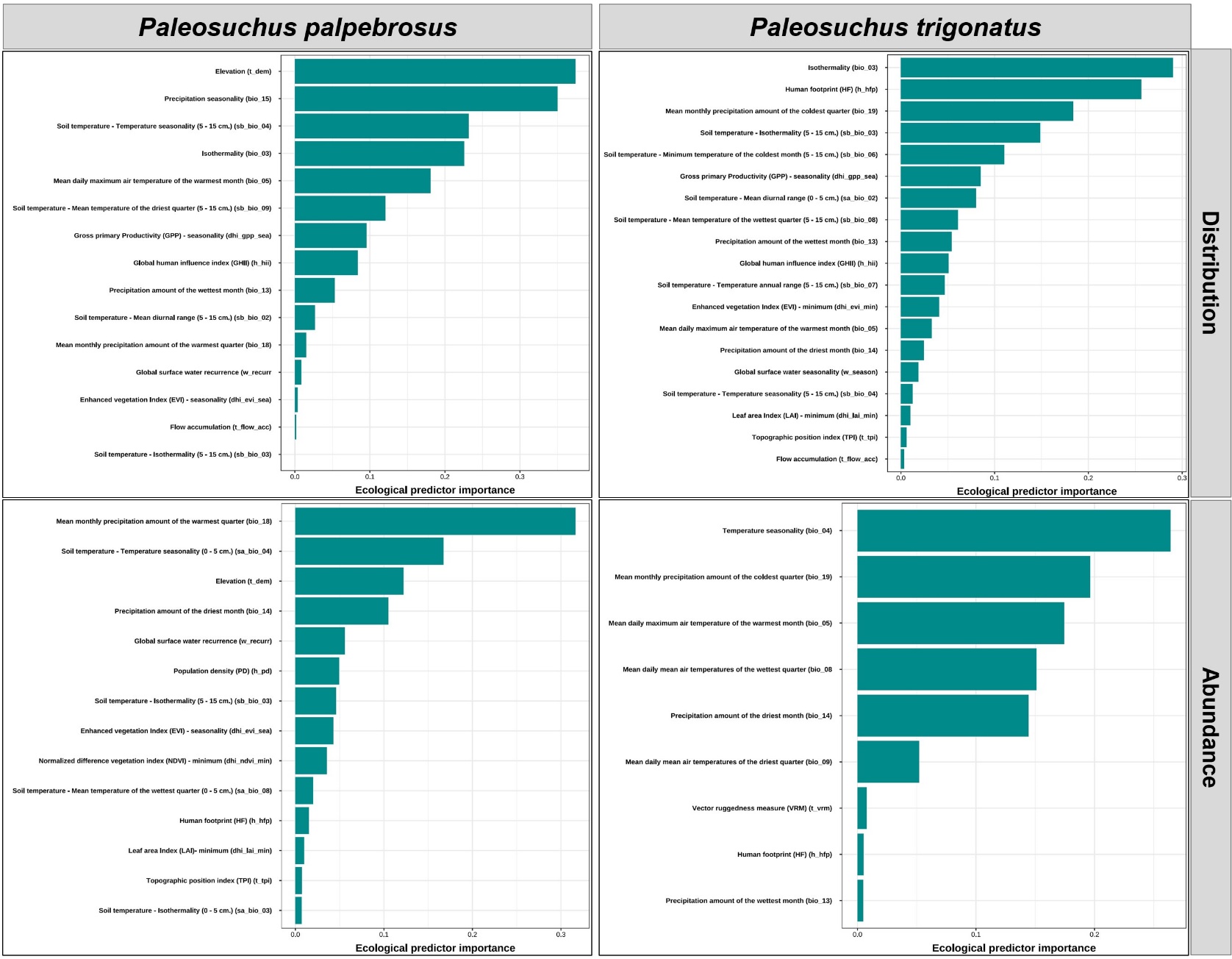


**Figure S3.** Variable importance of ecological predictors driving the occurrence and abundance of *Paleosuchus palpebrosus* (left insets) and *Paleosuchus trigonatus* (right insets). For distributional models, we used permutation-based variable-importance evaluation by applying the Pearson correlation between the original predictions and predictions where one variable has been 100 times randomly permutated. For abundance models, variable importance was obtained via the loss of predictive power excluding each ecological predictor at a time and calculating the mean explained deviance reduction.


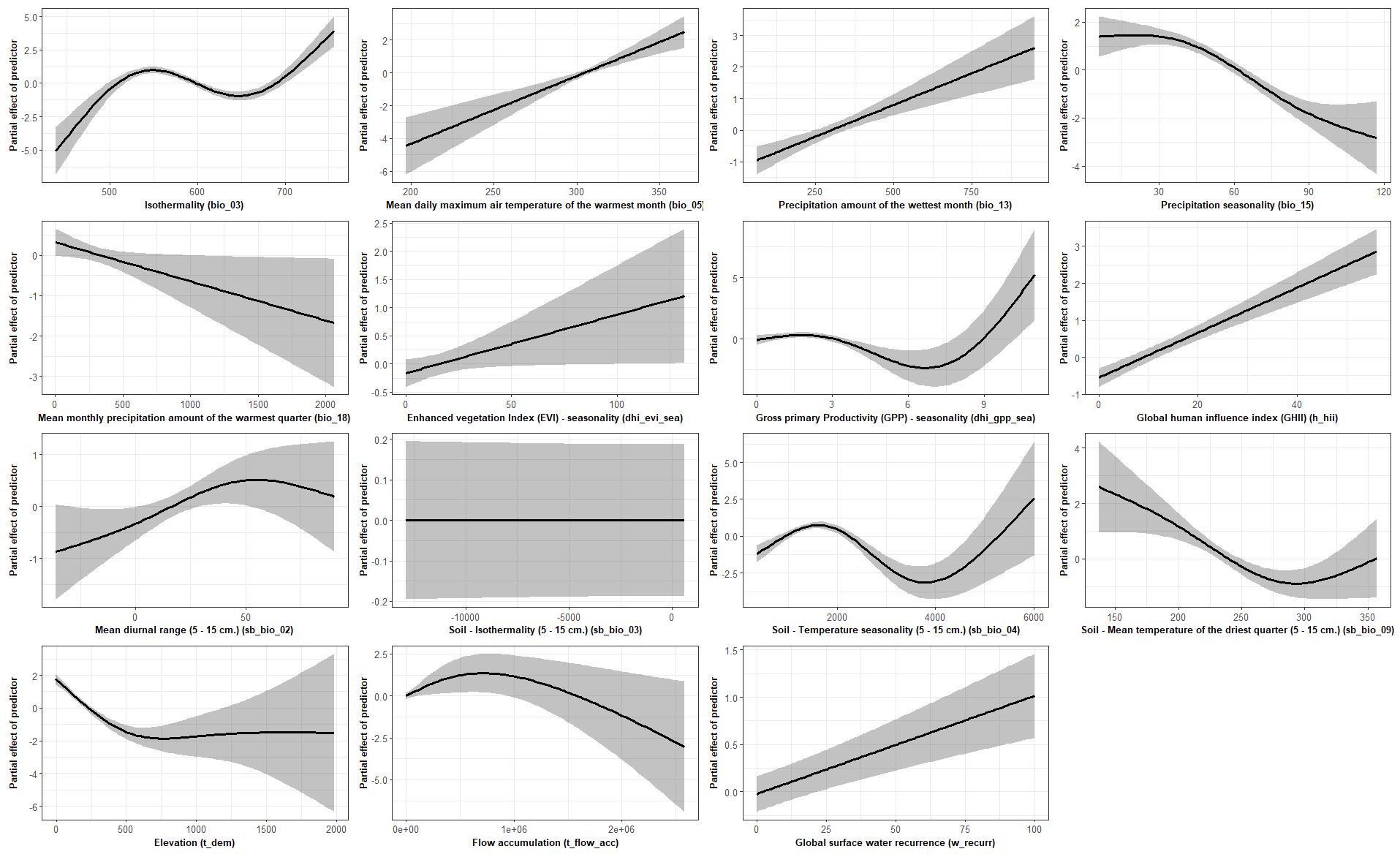


**Figure S4.** Partial response curves illustrating the relationship between species occurrence and the set of ecological predictors corresponding to the best predictive performance distributional model for *Paleosuchus palpebrosus* (GAM-S13).


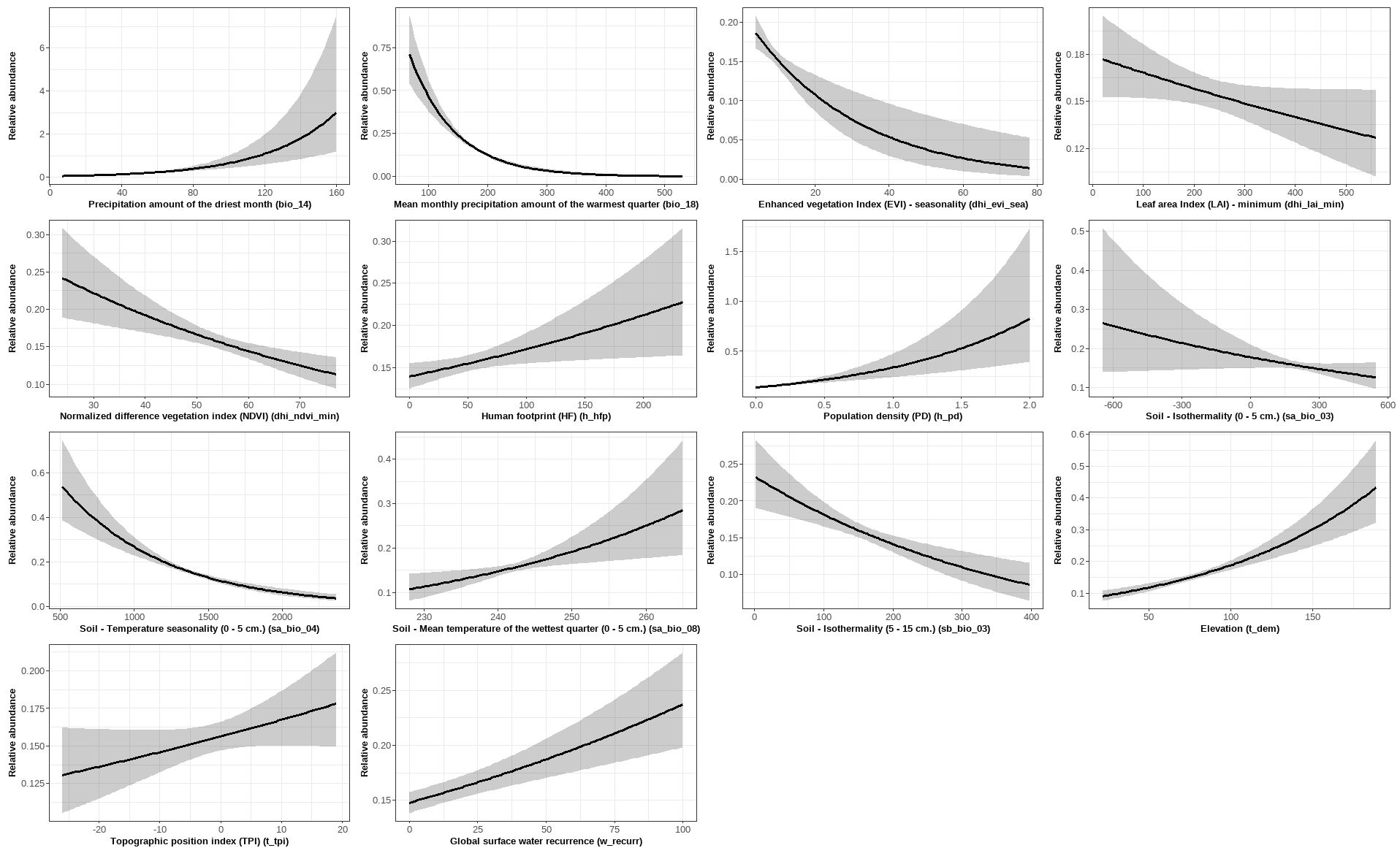


**Figure S5.** Partial response curves illustrating the relationship between species abundance and the set of ecological predictors corresponding to the best predictive performance abundance model for *Paleosuchus palpebrosus* (Poisson GLMsel-S12).


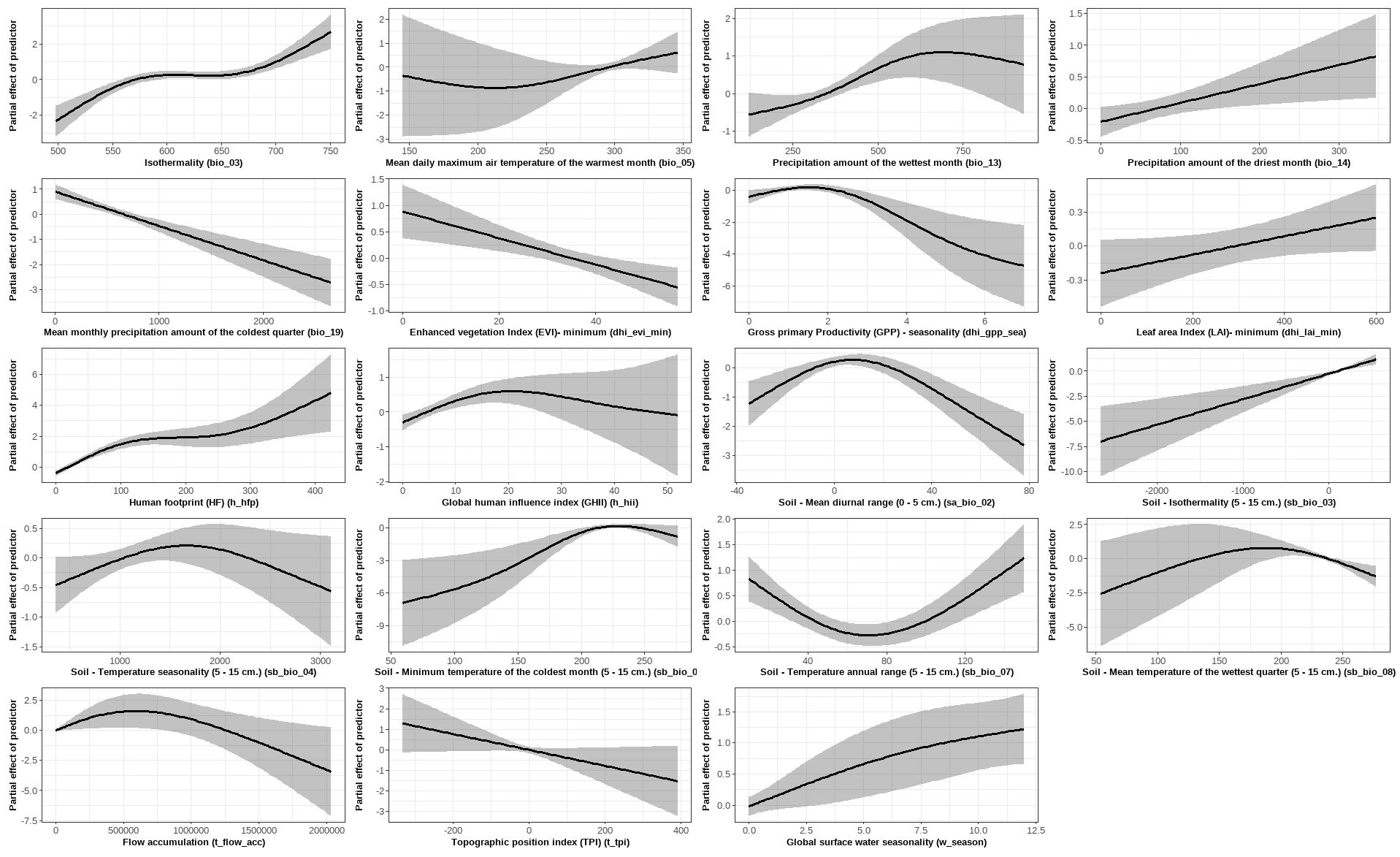


**Figure S6.** Partial response curves illustrating the relationship between species occurrence and the set of ecological predictors corresponding to the best predictive performance distributional model for *Paleosuchus trigonatus* (GAMsel-S12).


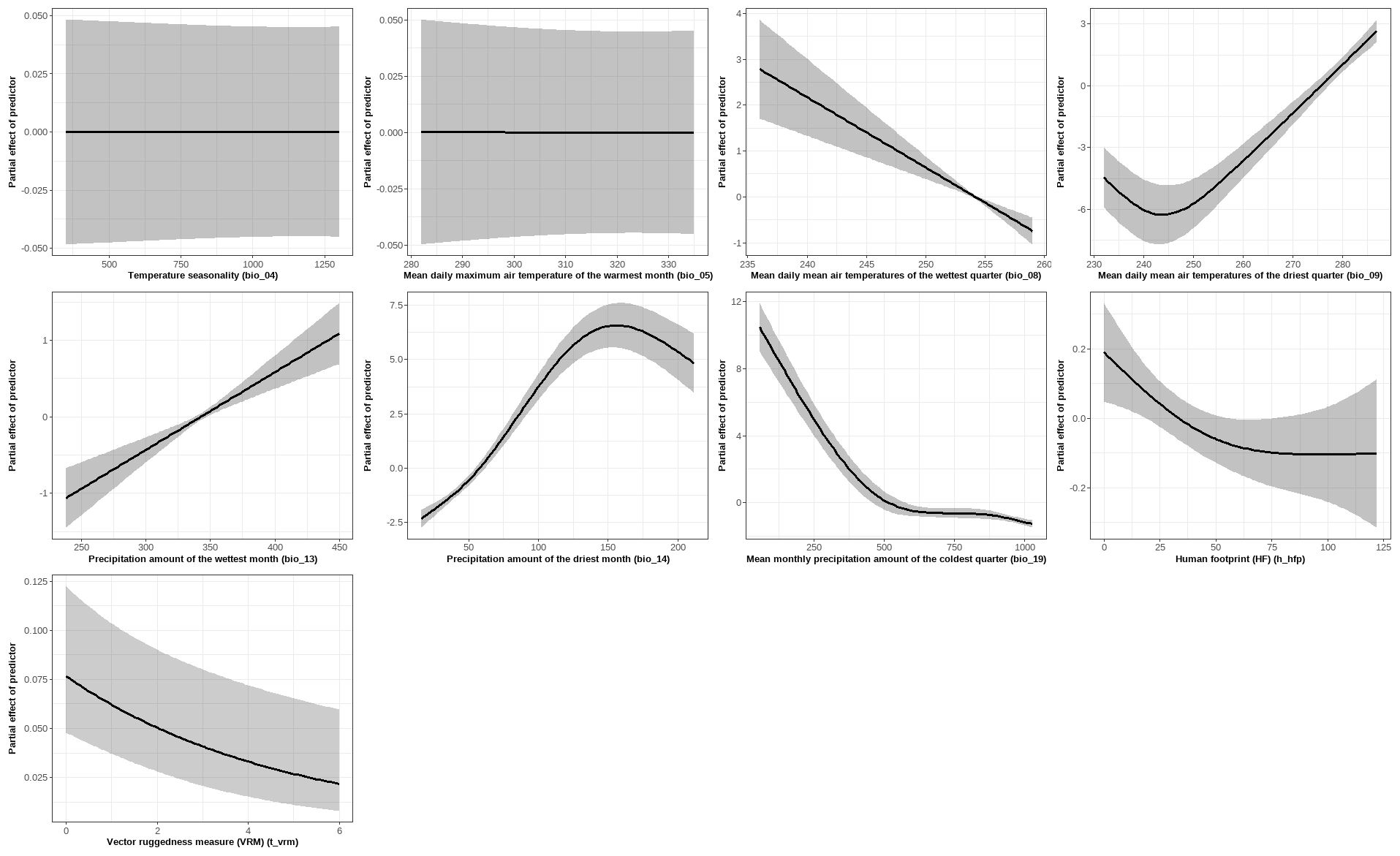


**Figure S7.** Partial response curves illustrating the relationship between species abundance and the set of ecological predictors corresponding to the best predictive performance abundance model for *Paleosuchus trigonatus* (Poisson GAMsel-S10).
